# Supplementary figures and images for: Nanopublication-based semantic publishing and reviewing: a field study with formalization papers
Source: PeerJ Comput Sci. 2023 Feb 21;9:e1159. doi: 10.7717/peerj-cs.1159 (PMC10280262; doi:10.7717/peerj-cs.1159)

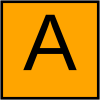

A

Supplement: Supplemental Information 1 — This repository contains data and code (SPARQL queries and scripting) to extract the published formalization articles (in the form of nanopublications) from the nanopublication decentralized network. It also contains the scripts made for the automatic visualization of these nanopublications in the form of a graph and a script to create the index for these nanopublications.These formalization articles will be published in the IOS Press journal Data Science, in a special issue with formalization articles in late March 2022. See https://github.com/LaraHack/fpsi_analytics [file peerj-cs-09-1159-s001.zip › fpsi_analytics-main/icons/cd-responses.pdf]

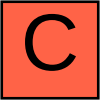

C

Supplement: Supplemental Information 1 — This repository contains data and code (SPARQL queries and scripting) to extract the published formalization articles (in the form of nanopublications) from the nanopublication decentralized network. It also contains the scripts made for the automatic visualization of these nanopublications in the form of a graph and a script to create the index for these nanopublications.These formalization articles will be published in the IOS Press journal Data Science, in a special issue with formalization articles in late March 2022. See https://github.com/LaraHack/fpsi_analytics [file peerj-cs-09-1159-s001.zip › fpsi_analytics-main/icons/classdefs.pdf]

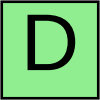

D

Supplement: Supplemental Information 1 — This repository contains data and code (SPARQL queries and scripting) to extract the published formalization articles (in the form of nanopublications) from the nanopublication decentralized network. It also contains the scripts made for the automatic visualization of these nanopublications in the form of a graph and a script to create the index for these nanopublications.These formalization articles will be published in the IOS Press journal Data Science, in a special issue with formalization articles in late March 2022. See https://github.com/LaraHack/fpsi_analytics [file peerj-cs-09-1159-s001.zip › fpsi_analytics-main/icons/decision.pdf]

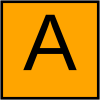

A

Supplement: Supplemental Information 1 — This repository contains data and code (SPARQL queries and scripting) to extract the published formalization articles (in the form of nanopublications) from the nanopublication decentralized network. It also contains the scripts made for the automatic visualization of these nanopublications in the form of a graph and a script to create the index for these nanopublications.These formalization articles will be published in the IOS Press journal Data Science, in a special issue with formalization articles in late March 2022. See https://github.com/LaraHack/fpsi_analytics [file peerj-cs-09-1159-s001.zip › fpsi_analytics-main/icons/sp-responses.pdf]

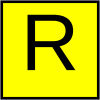

**R**

Supplement: Supplemental Information 1 — This repository contains data and code (SPARQL queries and scripting) to extract the published formalization articles (in the form of nanopublications) from the nanopublication decentralized network. It also contains the scripts made for the automatic visualization of these nanopublications in the form of a graph and a script to create the index for these nanopublications.These formalization articles will be published in the IOS Press journal Data Science, in a special issue with formalization articles in late March 2022. See https://github.com/LaraHack/fpsi_analytics [file peerj-cs-09-1159-s001.zip › fpsi_analytics-main/icons/sp-reviews.pdf]

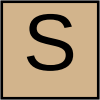

S

Supplement: Supplemental Information 1 — This repository contains data and code (SPARQL queries and scripting) to extract the published formalization articles (in the form of nanopublications) from the nanopublication decentralized network. It also contains the scripts made for the automatic visualization of these nanopublications in the form of a graph and a script to create the index for these nanopublications.These formalization articles will be published in the IOS Press journal Data Science, in a special issue with formalization articles in late March 2022. See https://github.com/LaraHack/fpsi_analytics [file peerj-cs-09-1159-s001.zip › fpsi_analytics-main/icons/submissions.pdf]

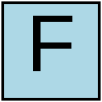

**F**

Supplement: Supplemental Information 1 — This repository contains data and code (SPARQL queries and scripting) to extract the published formalization articles (in the form of nanopublications) from the nanopublication decentralized network. It also contains the scripts made for the automatic visualization of these nanopublications in the form of a graph and a script to create the index for these nanopublications.These formalization articles will be published in the IOS Press journal Data Science, in a special issue with formalization articles in late March 2022. See https://github.com/LaraHack/fpsi_analytics [file peerj-cs-09-1159-s001.zip › fpsi_analytics-main/icons/superpatterns.pdf]

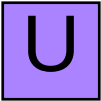

Supplement: Supplemental Information 1 — This repository contains data and code (SPARQL queries and scripting) to extract the published formalization articles (in the form of nanopublications) from the nanopublication decentralized network. It also contains the scripts made for the automatic visualization of these nanopublications in the form of a graph and a script to create the index for these nanopublications.These formalization articles will be published in the IOS Press journal Data Science, in a special issue with formalization articles in late March 2022. See https://github.com/LaraHack/fpsi_analytics [file peerj-cs-09-1159-s001.zip › fpsi_analytics-main/icons/updated.pdf]

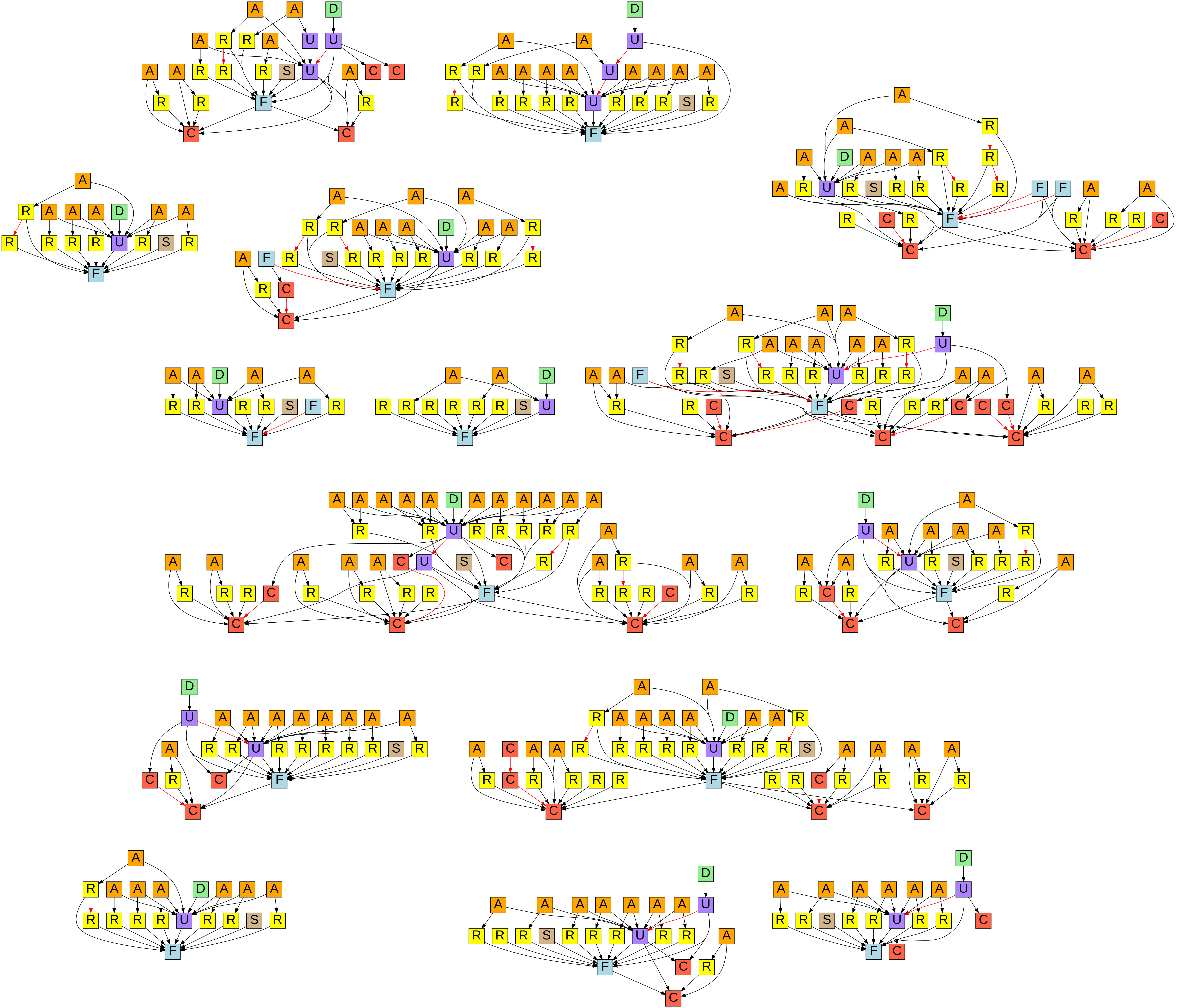

Supplement: Supplemental Information 1 — This repository contains data and code (SPARQL queries and scripting) to extract the published formalization articles (in the form of nanopublications) from the nanopublication decentralized network. It also contains the scripts made for the automatic visualization of these nanopublications in the form of a graph and a script to create the index for these nanopublications.These formalization articles will be published in the IOS Press journal Data Science, in a special issue with formalization articles in late March 2022. See https://github.com/LaraHack/fpsi_analytics [file peerj-cs-09-1159-s001.zip › fpsi_analytics-main/np-graph.pdf]
