# Supplementary material for: Nanopublication-based semantic publishing and reviewing: a field study with formalization papers
Source: PeerJ Comput Sci. 2023 Feb 21;9:e1159. doi: 10.7717/peerj-cs.1159 (PMC10280262; doi:10.7717/peerj-cs.1159)
Supplement: Supplemental Information 2 [file peerj-cs-09-1159-s002.zip › formalization_papers_supplemental-main/accepted_submissions/s10_Michel_Dumontier.docx]

**Title:** A formalization of one of the main claims of “The FAIR Guiding Principles for scientific data management and stewardship” by Wilkinson et al. 2016

**Authors:** Michel Dumontier, ORCID: 0000-0003-4727-9435

**Affiliations:** Maastricht University, The Netherlands. E-mail: [michel.dumontier@maastrichtuniversity.nl](mailto:michel.dumontier@maastrichtuniversity.nl) , [michel.dumontier@gmail.com](mailto:michel.dumontier@gmail.com)

**Keywords:** “data set”, “adherence to the FAIR guiding principles”, “automated discovery”

**Article Type:** Formalization Paper

**As RDF/nanopublication:** <http://purl.org/np/RA22JAQihYeiJkNIjvwnxLPmjuG74yPcRXpPyVX8DV6fA>

**Editor:** Cristina-Iulia Bucur, ORCID: 0000-0002-7114-6459

**Review comments from:**

- Tobias Kuhn, ORCID: 0000-0002-1267-0234
- Ricardo Usbeck, ORCID: 0000-0002-0191-7211
- Margherita Martorana, ORCID: 0000-0001-8004-0464
- Cristina-Iulia Bucur, ORCID: 0000-0002-7114-6459

**Received:** 2021-06-25

**Accepted:** 2021-11-17

**Abstract:**

Wilkinson et al. claimed in previous work that Adherence of a dataset to the FAIR Guiding Principles enables its automated discovery. We present here a formalization of that claim, stating that all things of class “adherence to the FAIR Guiding principles” that are in the context of a thing of class “data set” can generally have a relation of type “enables” to a thing of class “automated discovery” in the same context.

1. **Introduction**

Wilkinson et al. state that “the FAIR Principles put specific emphasis on enhancing the ability of machines to automatically find and use the data”. We present here a formalization of the main scientific claim from this quote by using a semantic template called the super-pattern [2].

1. **Formalization**

Our formalization looks as follows:

| CONTEXT-CLASS (“in the context of all ..."): | [data set](https://www.wikidata.org/wiki/Q1172284) |
| --- | --- |
| SUBJECT-CLASS (“things of type ..."): | [adherence to the FAIR guiding principles](http://purl.org/np/RAodU4AmRjfzyjwtJK3luO0iyRJJPUBjkijKWdlMHvack#adherenceToTheFAIRGuidingPrinciples) |
| QUALIFIER: | [can generally](https://w3id.org/linkflows/superpattern/terms/canGenerallyQualifier) |
| RELATION-TYPE (“have a relation of type...”): | [enables](https://w3id.org/linkflows/superpattern/terms/enables) |
| OBJECT-CLASS (“to things of type...”): | [automated discovery](http://purl.org/np/RAFQovt9yQD7nZ2tdZ9_Uhpb7CsfT3k64pK7dh63xd-50#automatedDiscovery) |

In the context class we use the “data set” (Q1172284) class from Wikidata. In subject class, we use a new minted class “adherence to the FAIR guiding principles” that is a subclass of “adherence” (Q85315455) and is related to the class “FAIR data principles” (Q29032644) from Wikidata. In the object class we minted a new class “automated discovery” that is a subclass of “discovery” (Q12772819) and is related to the class “automation” (Q184199) from Wikidata.

1. **RDF Code**

This is our formalization as a nanopublication in TriG format:

@prefix this: <http://purl.org/np/RA22JAQihYeiJkNIjvwnxLPmjuG74yPcRXpPyVX8DV6fA> .

@prefix sub: <http://purl.org/np/RA22JAQihYeiJkNIjvwnxLPmjuG74yPcRXpPyVX8DV6fA#> .

@prefix np: <http://www.nanopub.org/nschema#> .

@prefix dct: <http://purl.org/dc/terms/> .

@prefix nt: <https://w3id.org/np/o/ntemplate/> .

@prefix npx: <http://purl.org/nanopub/x/> .

@prefix xsd: <http://www.w3.org/2001/XMLSchema#> .

@prefix rdfs: <http://www.w3.org/2000/01/rdf-schema#> .

@prefix orcid: <https://orcid.org/> .

@prefix prov: <http://www.w3.org/ns/prov#> .

@prefix sp: <https://w3id.org/linkflows/superpattern/terms/> .

sub:Head {

this: np:hasAssertion sub:assertion ;

np:hasProvenance sub:provenance ;

np:hasPublicationInfo sub:pubinfo ;

a np:Nanopublication .

}

sub:assertion {

sub:spi a <https://w3id.org/linkflows/superpattern/terms/SuperPatternInstance> ;

rdfs:label "Adherence of a dataset to the FAIR Guiding Principles enables its automated discovery." ;

sp:hasContextClass <http://www.wikidata.org/entity/Q1172284> ;

sp:hasSubjectClass <http://purl.org/np/RAodU4AmRjfzyjwtJK3luO0iyRJJPUBjkijKWdlMHvack#adherenceToTheFAIRGuidingPrinciples> ;

sp:hasQualifier sp:canGenerallyQualifier ;

sp:hasRelation sp:enables ;

sp:hasObjectClass <http://purl.org/np/RAFQovt9yQD7nZ2tdZ9_Uhpb7CsfT3k64pK7dh63xd-50#automatedDiscovery> .

}

sub:provenance {

sub:activity a sp:FormalizationActivity ;

prov:used sub:quote , <https://doi.org/10.1038/sdata.2016.18> ;

prov:wasAssociatedWith orcid:0000-0003-4727-9435 .

sub:assertion prov:wasGeneratedBy sub:activity .

sub:quote prov:value "the FAIR Principles put specific emphasis on enhancing the ability of machines to automatically find and use the data" ;

prov:wasQuotedFrom <https://doi.org/10.1038/sdata.2016.18> .

}

sub:pubinfo {

sub:sig npx:hasAlgorithm "RSA" ;

npx:hasPublicKey "MIGfMA0GCSqGSIb3DQEBAQUAA4GNADCBiQKBgQCTQs+mANCSHWhIW/YPio468UdGNHsPvADpjfaW8um/v2L4AoDIANginfoU65VNbPT5D0ADt1y0uFNne3VEMr9Y+I2HFaz6IKj+LdYMJk6VUf5WJoImRHIX6BZQwcUc22CbTBFYxvqvp3UmmHrCrhLIZjDSyutExK3tOTRoMDjGowIDAQAB" ;

npx:hasSignature "hHeN9qAHbRXgslk6ztdFWPThPTPYrIjclGL+nH6YX7A88Qqj70dJFXZYBFGcv7OpOiEmEVsAlNs2Xn7oefCmpsfBTIivwVPLf8SWzXrpnDU2p9naIr6YMlyrNJ3wLg61pXWaOH82njsVr1GMtL7y0VGw8cCmhdvzASigRCfLAQs=" ;

npx:hasSignatureTarget this: .

this: dct:created "2021-11-08T09:09:11.999+01:00"^^xsd:dateTime ;

dct:creator orcid:0000-0003-4727-9435 ;

npx:introduces sub:spi ;

<https://w3id.org/linkflows/reviews/isUpdateOf> <http://purl.org/np/RAPLWOEA5t6_Kx6vVHtibWm92pg1XTN6FIZdWuaTyoy9g> ;

nt:wasCreatedFromProvenanceTemplate <http://purl.org/np/RAE1wniOy0yO39PlK9QkQ-wqbC3q-R2nXraP5huu8W39k> ;

nt:wasCreatedFromPubinfoTemplate <http://purl.org/np/RAA2MfqdBCzmz9yVWjKLXNbyfBNcwsMmOqcNUxkk1maIM> , <http://purl.org/np/RAOGu9Lh0BD4tbIRB9RG6RGRA_ObDh75NTbIqaWgxxs8M> ;

nt:wasCreatedFromTemplate <http://purl.org/np/RAv68imZrEjfcp2rnEg1hzoBqEVc0cQMtp9_1Za0BxNM4> .

}

The following nanopublications introduce the newly minted classes in TriG format.

This is the class definition of “adherence to the FAIR guiding principles”:

@prefix this: <http://purl.org/np/RAodU4AmRjfzyjwtJK3luO0iyRJJPUBjkijKWdlMHvack> .

@prefix sub: <http://purl.org/np/RAodU4AmRjfzyjwtJK3luO0iyRJJPUBjkijKWdlMHvack#> .

@prefix np: <http://www.nanopub.org/nschema#> .

@prefix dct: <http://purl.org/dc/terms/> .

@prefix nt: <https://w3id.org/np/o/ntemplate/> .

@prefix npx: <http://purl.org/nanopub/x/> .

@prefix xsd: <http://www.w3.org/2001/XMLSchema#> .

@prefix rdfs: <http://www.w3.org/2000/01/rdf-schema#> .

@prefix orcid: <https://orcid.org/> .

@prefix prov: <http://www.w3.org/ns/prov#> .

@prefix skos: <http://www.w3.org/2004/02/skos/core#> .

sub:Head {

this: np:hasAssertion sub:assertion ;

np:hasProvenance sub:provenance ;

np:hasPublicationInfo sub:pubinfo ;

a np:Nanopublication .

}

sub:assertion {

sub:adherenceToTheFAIRGuidingPrinciples a <http://www.w3.org/2002/07/owl#Class> ;

rdfs:label "adherence to the FAIR Guiding Principles" ;

rdfs:subClassOf <http://www.wikidata.org/entity/Q85315455> ;

skos:definition "everything that adheres to the FAIR Guiding Principles" ;

skos:relatedMatch <http://www.wikidata.org/entity/Q29032644> .

}

sub:provenance {

sub:assertion prov:wasAttributedTo orcid:0000-0003-4727-9435 .

}

sub:pubinfo {

sub:sig npx:hasAlgorithm "RSA" ;

npx:hasPublicKey "MIGfMA0GCSqGSIb3DQEBAQUAA4GNADCBiQKBgQCTQs+mANCSHWhIW/YPio468UdGNHsPvADpjfaW8um/v2L4AoDIANginfoU65VNbPT5D0ADt1y0uFNne3VEMr9Y+I2HFaz6IKj+LdYMJk6VUf5WJoImRHIX6BZQwcUc22CbTBFYxvqvp3UmmHrCrhLIZjDSyutExK3tOTRoMDjGowIDAQAB" ;

npx:hasSignature "FU2AFDaNFi5T1Fg9rVBkFRUGzO1XkE+kB6avmaGd2vxwc+1qHRQ39yVXfLs4lEZll9QURYWVcAT9ogv+rs/ZMkyboGgEHPSFzogtUPykLqimfEh+XK2TMvcPP71W3GRJqjmo/ZJ/7CGocR022MkmYf0IeyzPWRTrAdD41IXDvfs=" ;

npx:hasSignatureTarget this: .

this: dct:created "2021-06-25T16:31:54.801+02:00"^^xsd:dateTime ;

dct:creator orcid:0000-0003-4727-9435 ;

npx:introduces sub:adherenceToTheFAIRGuidingPrinciples ;

nt:wasCreatedFromProvenanceTemplate <http://purl.org/np/RANwQa4ICWS5SOjw7gp99nBpXBasapwtZF1fIM3H2gYTM> ;

nt:wasCreatedFromPubinfoTemplate <http://purl.org/np/RAA2MfqdBCzmz9yVWjKLXNbyfBNcwsMmOqcNUxkk1maIM> ;

nt:wasCreatedFromTemplate <http://purl.org/np/RAdpgRpigXtt8iPV9uOPf3wIT3qzOI8Sg2Q72CNV8g-Yo> .

}

This is the class definition of “automated discovery”:

@prefix this: <http://purl.org/np/RAFQovt9yQD7nZ2tdZ9_Uhpb7CsfT3k64pK7dh63xd-50> .

@prefix sub: <http://purl.org/np/RAFQovt9yQD7nZ2tdZ9_Uhpb7CsfT3k64pK7dh63xd-50#> .

@prefix np: <http://www.nanopub.org/nschema#> .

@prefix dct: <http://purl.org/dc/terms/> .

@prefix nt: <https://w3id.org/np/o/ntemplate/> .

@prefix npx: <http://purl.org/nanopub/x/> .

@prefix xsd: <http://www.w3.org/2001/XMLSchema#> .

@prefix rdfs: <http://www.w3.org/2000/01/rdf-schema#> .

@prefix orcid: <https://orcid.org/> .

@prefix prov: <http://www.w3.org/ns/prov#> .

@prefix skos: <http://www.w3.org/2004/02/skos/core#> .

sub:Head {

this: np:hasAssertion sub:assertion ;

np:hasProvenance sub:provenance ;

np:hasPublicationInfo sub:pubinfo ;

a np:Nanopublication .

}

sub:assertion {

sub:automatedDiscovery a <http://www.w3.org/2002/07/owl#Class> ;

rdfs:label "Automated Discovery" ;

rdfs:subClassOf <http://www.wikidata.org/entity/Q12772819> ;

skos:definition "Everything that is discoverable through automation" ;

skos:relatedMatch <http://www.wikidata.org/entity/Q184199> .

}

sub:provenance {

sub:assertion prov:wasAttributedTo orcid:0000-0003-4727-9435 .

}

sub:pubinfo {

sub:sig npx:hasAlgorithm "RSA" ;

npx:hasPublicKey "MIGfMA0GCSqGSIb3DQEBAQUAA4GNADCBiQKBgQCTQs+mANCSHWhIW/YPio468UdGNHsPvADpjfaW8um/v2L4AoDIANginfoU65VNbPT5D0ADt1y0uFNne3VEMr9Y+I2HFaz6IKj+LdYMJk6VUf5WJoImRHIX6BZQwcUc22CbTBFYxvqvp3UmmHrCrhLIZjDSyutExK3tOTRoMDjGowIDAQAB" ;

npx:hasSignature "hji2+/D20zQjL8dUsjumjW760vBuClRwU4luJQL8PvMjaenF0hFWf/1qRIqqVLjJ9/unF5nVM2kWKi8frsruGqZdGzy3/8gKnuH6D1RROX3Z1lVqEJJQ8a5gG18ZpArQ7JNRUFYnCDsfFgEaGlFM8HNw4ECttpJUvDlYlnuPxKU=" ;

npx:hasSignatureTarget this: .

this: dct:created "2021-06-25T16:45:52.256+02:00"^^xsd:dateTime ;

dct:creator orcid:0000-0003-4727-9435 ;

npx:introduces sub:automatedDiscovery ;

nt:wasCreatedFromProvenanceTemplate <http://purl.org/np/RANwQa4ICWS5SOjw7gp99nBpXBasapwtZF1fIM3H2gYTM> ;

nt:wasCreatedFromPubinfoTemplate <http://purl.org/np/RAA2MfqdBCzmz9yVWjKLXNbyfBNcwsMmOqcNUxkk1maIM> ;

nt:wasCreatedFromTemplate <http://purl.org/np/RAdpgRpigXtt8iPV9uOPf3wIT3qzOI8Sg2Q72CNV8g-Yo> .

}

**References**

[1] Wilkinson, M., Dumontier, M., Aalbersberg, I. et al. The FAIR Guiding Principles for scientific data management and stewardship. Sci Data 3, 160018 (2016). doi: [10.1038/sdata.2016.18](https://doi-org.vu-nl.idm.oclc.org/10.1038/sdata.2016.18).

[2] Bucur, C.I., Kuhn, T., Ceolin, D., Ossenbruggen, J. van. Expressing high-level scientific claims with formal semantics. In: Proceedings of the 11th Knowledge Capture Conference 2021. doi: 10.1145/3460210.3493561.
