# Supplementary material for: Nanopublication-based semantic publishing and reviewing: a field study with formalization papers
Source: PeerJ Comput Sci. 2023 Feb 21;9:e1159. doi: 10.7717/peerj-cs.1159 (PMC10280262; doi:10.7717/peerj-cs.1159)
Supplement: Supplemental Information 2 [file peerj-cs-09-1159-s002.zip › formalization_papers_supplemental-main/accepted_submissions/s10_Michel_Dumontier.pdf]

Title: A formalization of one of the main claims of “The FAIR Guiding Principles for scientific data management and stewardship” by Wilkinson et al. 2016

Authors: Michel Dumontier, ORCID: 0000-0003-4727-9435

Affiliations: Maastricht University, The Netherlands. E-mail: michel.dumontier@maastrichtuniversity.nl , michel.dumontier@gmail.com

Keywords: “data set”, “adherence to the FAIR guiding principles”, “automated discovery”

Article Type: Formalization Paper

As RDF/nanopublication:
http://purl.org/np/RA22JAQihYeiJkNIjvwnxLPmjuG74yPcRXpPyVX8DV6fA

Editor: Cristina-Iulia Bucur, ORCID: 0000-0002-7114-6459

Review comments from:
- Tobias Kuhn, ORCID: 0000-0002-1267-0234
- Ricardo Usbeck, ORCID: 0000-0002-0191-7211
- Margherita Martorana, ORCID: 0000-0001-8004-0464
- Cristina-Iulia Bucur, ORCID: 0000-0002-7114-6459

Received: 2021-06-25

Accepted: 2021-11-17

Abstract:
Wilkinson et al. claimed in previous work that Adherence of a dataset to the FAIR Guiding Principles enables its automated discovery. We present here a formalization of that claim, stating that all things of class “adherence to the FAIR Guiding principles” that are in the context of a thing of class “data set” can generally have a relation of type “enables” to a thing of class “automated discovery” in the same context.

1. Introduction

Wilkinson et al. state that “the FAIR Principles put specific emphasis on enhancing the ability of machines to automatically find and use the data”. We present here a formalization of the main scientific claim from this quote by using a semantic template called the super-pattern [2].

2. Formalization

Our formalization looks as follows:

CONTEXT-CLASS (“in the context of all ..."):	data set
SUBJECT-CLASS (“things of type ..."):	adherence to the FAIR guiding principles
QUALIFIER:	can generally
RELATION-TYPE (“have a relation of type...”):	enables
OBJECT-CLASS (“to things of type...”):	automated discovery

In the context class we use the “data set” (Q1172284) class from Wikidata. In subject class, we use a new minted class “adherence to the FAIR guiding principles” that is a subclass of “adherence” (Q85315455) and is related to the class “FAIR data principles” (Q29032644) from Wikidata. In the object class we minted a new class “automated discovery” that is a subclass of “discovery” (Q12772819) and is related to the class “automation” (Q184199) from Wikidata.

3. RDF Code

This is our formalization as a nanopublication in TriG format:

```
@prefix this: <http://purl.org/np/RA22JAQihYeiJkNIjvwnxLPmjuG74yPcRXpPyVX8DV6fA> .
@prefix sub: <http://purl.org/np/RA22JAQihYeiJkNIjvwnxLPmjuG74yPcRXpPyVX8DV6fA#> .
@prefix np: <http://www.nanopub.org/nschema#> .
@prefix dct: <http://purl.org/dc/terms/> .
@prefix nt: <https://w3id.org/np/o/ntemplate/> .
@prefix npx: <http://purl.org/nanopub/x/> .
@prefix xsd: <http://www.w3.org/2001/XMLSchema#> .
@prefix rdfs: <http://www.w3.org/2000/01/rdf-schema#> .
@prefix orcid: <https://orcid.org/> .
@prefix prov: <http://www.w3.org/ns/prov#> .
@prefix sp: <https://w3id.org/linkflows/superpattern/terms/> .


sub:Head {
  this: np:hasAssertion sub:assertion ;
    np:hasProvenance sub:provenance ;
    np:hasPublicationInfo sub:pubinfo ;
    a np:Nanopublication .
}
sub:assertion {
  sub:spi a <https://w3id.org/linkflows/superpattern/terms/SuperPatternInstance> ;
    rdfs:label "Adherence of a dataset to the FAIR Guiding Principles enables its automated discovery." ;
    sp:hasContextClass <http://www.wikidata.org/entity/Q1172284> ;
    sp:hasSubjectClass <http://purl.org/np/RAodU4AmRjfzyjwtJK3luO0iyRJJPUBjkijKWdlMHvack#adherenceToTheFAIRGuidingPrinciples> ;
    sp:hasQualifier sp:canGenerallyQualifier ;
    sp:hasRelation sp:enables ;
    sp:hasObjectClass <http://purl.org/np/RAFQovt9yQD7nZ2tdZ9_Uhpb7CsfT3k64pK7dh63xd-50#automatedDiscovery> .
}
sub:provenance {
  sub:activity a sp:FormalizationActivity ;
    prov:used sub:quote , <https://doi.org/10.1038/sdata.2016.18> ;
    prov:wasAssociatedWith orcid:0000-0003-4727-9435 .
  sub:assertion prov:wasGeneratedBy sub:activity .
  sub:quote prov:value "the FAIR Principles put specific emphasis on enhancing the ability of machines to automatically find and 
use the data" ;
    prov:wasQuotedFrom <https://doi.org/10.1038/sdata.2016.18> .
}
sub:pubinfo {
  sub:sig npx:hasAlgorithm "RSA" ;
    npx:hasPublicKey 
"MIGfMA0GCSqGSIb3DQEBAQUAA4GNADCBiQKBgQCTQs+mANCSHWhIW/YPio468UdGNHsPvADpjfaW8um/v2L4AoDIANginfoU65VNbPT5D0ADt1y0uFNne3VEMr9Y+I2H
Faz6IKj+LdYMJk6VUf5WJoImRHIX6BZQwcUc22CbTBFYxvqvp3UmmHrCrhLIZjDSyutExK3tOTRoMDjGowIDAQAB" ;
    npx:hasSignature 
"hHeN9qAHbRXgslk6ztdFWPThPTPYrIjclGL+nH6YX7A88Qqj70dJFXZYBFGcv7OpOiEmEVsAlNs2Xn7oefCmpsfBTIivwVPLf8SWzXrpnDU2p9naIr6YMlyrNJ3wLg61
pXWaOH82njsVr1GMtL7y0VGw8cCmhdvzASigRCfLAQs=" ;
```

```
    npx:hasSignatureTarget this: .
  this: dct:created "2021-11-08T09:09:11.999+01:00"^^xsd:dateTime ;
    dct:creator orcid:0000-0003-4727-9435 ;
    npx:introduces sub:spi ;
    <https://w3id.org/linkflows/reviews/isUpdateOf> <http://purl.org/np/RAPLWOEA5t6_Kx6vVHtibWm92pg1XTN6FIZdWuaTyoy9g> ;
    nt:wasCreatedFromProvenanceTemplate <http://purl.org/np/RAE1wniOy0yO39PlK9QkQ-wqbC3q-R2nXraP5huu8W39k> ;
    nt:wasCreatedFromPubinfoTemplate <http://purl.org/np/RAA2MfqdBCzmz9yVWjKLXNbyfBNcwsMmOqcNUxkk1maIM> ,
<http://purl.org/np/RAOGu9Lh0BD4tbIRB9RG6RGRA_ObDh75NTbIqaWgxxs8M> ;
    nt:wasCreatedFromTemplate <http://purl.org/np/RAv68imZrEjfcp2rnEg1hzoBqEVc0cQMtp9_1Za0BxNM4> .
}
```

The following nanopublications introduce the newly minted classes in TriG format.

This is the class definition of “adherence to the FAIR guiding principles”:

```
@prefix this: <http://purl.org/np/RAodU4AmRjfzyjwtJK3luO0iyRJJPUBjkijKWdlMHvack> .
@prefix sub: <http://purl.org/np/RAodU4AmRjfzyjwtJK3luO0iyRJJPUBjkijKWdlMHvack#> .
@prefix np: <http://www.nanopub.org/nschema#> .
@prefix dct: <http://purl.org/dc/terms/> .
@prefix nt: <https://w3id.org/np/o/ntemplate/> .
@prefix npx: <http://purl.org/nanopub/x/> .
@prefix xsd: <http://www.w3.org/2001/XMLSchema#> .
@prefix rdfs: <http://www.w3.org/2000/01/rdf-schema#> .
@prefix orcid: <https://orcid.org/> .
@prefix prov: <http://www.w3.org/ns/prov#> .
@prefix skos: <http://www.w3.org/2004/02/skos/core#> .


sub:Head {
  this: np:hasAssertion sub:assertion ;
    np:hasProvenance sub:provenance ;
    np:hasPublicationInfo sub:pubinfo ;
    a np:Nanopublication .
}
sub:assertion {
  sub:adherenceToTheFAIRGuidingPrinciples a <http://www.w3.org/2002/07/owl#Class> ;
    rdfs:label "adherence to the FAIR Guiding Principles" ;
    rdfs:subClassOf <http://www.wikidata.org/entity/Q85315455> ;
    skos:definition "everything that adheres to the FAIR Guiding Principles" ;
    skos:relatedMatch <http://www.wikidata.org/entity/Q29032644> .
}
sub:provenance {
  sub:assertion prov:wasAttributedTo orcid:0000-0003-4727-9435 .
}
sub:pubinfo {
  sub:sig npx:hasAlgorithm "RSA" ;
    npx:hasPublicKey
"MIGfMA0GCSqGSIb3DQEBAQUAA4GNADCBiQKBgQCTQs+mANCSHWhIW/YPio468UdGNHsPvADpjfaW8um/v2L4AoDIANginfoU65VNbPT5D0ADt1y0uFNne3VEMr9Y+I2H
Faz6IKj+LdYMJk6VUf5WJoImRHIX6BZQwcUc22CbTBFYxvqvp3UmmHrCrhLIZjDSyutExK3tOTRoMDjGowIDAQAB" ;
    npx:hasSignature
"FU2AFDaNFi5T1Fg9rVBkFRUGzO1XkE+kB6avmaGd2vxwc+1qHRQ39yVXfLs4lEZl19QURYWVcAT9ogv+rs/ZMkyboGgEHPSFzogtUPykLqimfEh+XK2TMvcPP71W3GRJ
qjmo/ZJ/7CGocR022MkmYf0IeyzPWRTrAdD41IXDvfs=" ;
    npx:hasSignatureTarget this: .
  this: dct:created "2021-06-25T16:31:54.801+02:00"^^xsd:dateTime ;
    dct:creator orcid:0000-0003-4727-9435 ;
    npx:introduces sub:adherenceToTheFAIRGuidingPrinciples ;
    nt:wasCreatedFromProvenanceTemplate <http://purl.org/np/RANwQa4ICWS5SOjw7gp99nBpXBasapwtZF1fIM3H2gYTM> ;
    nt:wasCreatedFromPubinfoTemplate <http://purl.org/np/RAA2MfqdBCzmz9yVWjKLXNbyfBNcwsMmOqcNUxkk1maIM> ;
    nt:wasCreatedFromTemplate <http://purl.org/np/RAdpgRpigXtt8iPV9uOPf3wIT3qzOI8Sg2Q72CNV8g-Yo> .
}
```

This is the class definition of “automated discovery”:

```
@prefix this: <http://purl.org/np/RAFQovt9yQD7nZ2tdZ9_Uhpb7CsfT3k64pK7dh63xd-50> .
@prefix sub: <http://purl.org/np/RAFQovt9yQD7nZ2tdZ9_Uhpb7CsfT3k64pK7dh63xd-50#> .
@prefix np: <http://www.nanopub.org/nschema#> .
@prefix dct: <http://purl.org/dc/terms/> .
@prefix nt: <https://w3id.org/np/o/ntemplate/> .
@prefix npx: <http://purl.org/nanopub/x/> .
@prefix xsd: <http://www.w3.org/2001/XMLSchema#> .
@prefix rdfs: <http://www.w3.org/2000/01/rdf-schema#> .
@prefix orcid: <https://orcid.org/> .
@prefix prov: <http://www.w3.org/ns/prov#> .
@prefix skos: <http://www.w3.org/2004/02/skos/core#> .
```

```
sub:Head {
  this: np:hasAssertion sub:assertion ;
    np:hasProvenance sub:provenance ;
    np:hasPublicationInfo sub:pubinfo ;
    a np:Nanopublication .
}
sub:assertion {
  sub:automatedDiscovery a <http://www.w3.org/2002/07/owl#Class> ;
    rdfs:label "Automated Discovery" ;
    rdfs:subClassOf <http://www.wikidata.org/entity/Q12772819> ;
    skos:definition "Everything that is discoverable through automation" ;
    skos:relatedMatch <http://www.wikidata.org/entity/Q184199> .
}
sub:provenance {
  sub:assertion prov:wasAttributedTo orcid:0000-0003-4727-9435 .
}
sub:pubinfo {
  sub:sig npx:hasAlgorithm "RSA" ;
    npx:hasPublicKey
"MIGfMA0GCSqGSIb3DQEBAQUAA4GNADCBiQKBgQCTQs+mANCSHWhIW/YPio468UdGNHsPvADpjfaW8um/v2L4AoDIANginfoU65VNbPT5D0ADt1y0uFNne3VEMr9Y+I2H
Faz6IKj+LdYMJk6VUf5WJoImRHIX6BZQwcUc22CbTBFYxvqvp3UmmHrCrhLIZjDSyutExK3tOTRoMDjGowIDAQAB" ;
    npx:hasSignature
"hji2+/D20zQjL8dUsjumjW760vBuClRwU4luJQL8PvMjaenF0hFWf/1qRIqqVLjJ9/unF5nVM2kWKi8frsruGqZdGzy3/8gKnuH6D1RROX3Z1lVqEJJQ8a5gG18ZpArQ
7JNRUFYnCDsfFgEaGlFM8HNw4ECttpJUvDlYlnuPxKU=" ;
    npx:hasSignatureTarget this: .
  this: dct:created "2021-06-25T16:45:52.256+02:00"^^xsd:dateTime ;
    dct:creator orcid:0000-0003-4727-9435 ;
    npx:introduces sub:automatedDiscovery ;
    nt:wasCreatedFromProvenanceTemplate <http://purl.org/np/RANwQa4ICWS5SOjw7gp99nBpXBasapwtZF1fIM3H2gYTM> ;
    nt:wasCreatedFromPubinfoTemplate <http://purl.org/np/RAA2MfqdBCzmz9yVWjKLXNbyfBNcwsMmOqcNUxkk1maIM> ;
    nt:wasCreatedFromTemplate <http://purl.org/np/RAdpgRpigXtt8iPV9uOPf3wIT3qzOI8Sg2Q72CNV8g-Yo> .
}
```

References

[1] Wilkinson, M., Dumontier, M., Aalbersberg, I. et al. The FAIR Guiding Principles for scientific data management and stewardship. Sci Data 3, 160018 (2016). doi: 10.1038/sdata.2016.18.

[2] Bucur, C.I., Kuhn, T., Ceolin, D., Ossenbruggen, J. van. Expressing high-level scientific claims with formal semantics. In: Proceedings of the 11th Knowledge Capture Conference 2021. doi: 10.1145/3460210.3493561.
