# Supplementary material for: Nanopublication-based semantic publishing and reviewing: a field study with formalization papers
Source: PeerJ Comput Sci. 2023 Feb 21;9:e1159. doi: 10.7717/peerj-cs.1159 (PMC10280262; doi:10.7717/peerj-cs.1159)
Supplement: Supplemental Information 2 [file peerj-cs-09-1159-s002.zip › formalization_papers_supplemental-main/accepted_submissions/s11_Núria_Queralt_Rosinach.docx]

**Title:** A formalization of one of the main claims of “Mutations in NGLY1 cause an inherited disorder of the endoplasmic reticulum–associated degradation pathway” by Enns et al. 2014

**Authors:** Núria Queralt Rosinach, ORCID: 0000-0003-0169-8159

**Affiliations:** Leiden University Medical Center, Leiden, The Netherlands. E-mail: [nqueralt.r@gmail.com](mailto:nqueralt.r@gmail.com)

**Keywords:** “human”, “NGLY1 deficiency”, “dysfunction of ERAD pathway”

**Article Type:** Formalization Paper

**As RDF/nanopublication:** <http://purl.org/np/RA12lVwEtmddK9OwDkZQZlgJaOD2-0NXtAtO_jDaG-3VQ>

**Editor:** Cristina-Iulia Bucur, ORCID: 0000-0002-7114-6459

**Review comments from:**

- Tobias Kuhn, ORCID: 0000-0002-1267-0234
- Cristina-Iulia Bucur, ORCID: 0000-0002-7114-6459

**Received:** 2021-06-03

**Accepted:** 2021-12-17

**Abstract:**

Enns et al. claimed in previous work that NGLY1 deficiency is a novel autosomal recessive disorder of the ERAD pathway. We present here a formalization of that claim, stating that all things of class “NGLY1 deficiency” that are in the context of a thing of class “human” always have a relation of type “is caused by” to a thing of class “dysfunction of ERAD pathway” in the same context.

1. **Introduction**

Enns et al. [1] state that “NGLY1 deficiency is a novel autosomal recessive disorder of the endoplasmic reticulum–associated degradation pathway.”. We present here a formalization of the main scientific claim from this quote by using a semantic template called the super-pattern [2].

1. **Formalization**

Our formalization looks as follows:

| CONTEXT-CLASS (“in the context of all ..."): | [human](http://www.wikidata.org/entity/Q5) |
| --- | --- |
| SUBJECT-CLASS (“things of type ..."): | [NGLY1 deficiency](http://purl.obolibrary.org/obo/MONDO_0014109) |
| QUALIFIER: | [always](https://w3id.org/linkflows/superpattern/terms/alwaysQualifier) |
| RELATION-TYPE (“have a relation of type...”): | [is caused by](https://w3id.org/linkflows/superpattern/terms/isCausedBy) |
| OBJECT-CLASS (“to things of type...”): | [dysfunction of ERAD pathway](http://purl.org/np/RAZVLqlkbwiX40n0GNxcxJany2Cw3oxMCrNuZtjBClryU#Dysfunction_of_ERAD_pathway) |

In the context class we use the class “human” (Q5) from Wikidata. In the subject class, we use the class “NGLY1 deficiency” from Mondo Disease Ontology. In the object class we minted a new class “dysfunction of ERAD pathway” that is related to the class “ERAD pathway” (Q21101062) from Wikidata.

1. **RDF Code**

This is our formalization as a nanopublication in TriG format:

@prefix this: <http://purl.org/np/RA12lVwEtmddK9OwDkZQZlgJaOD2-0NXtAtO_jDaG-3VQ> .

@prefix sub: <http://purl.org/np/RA12lVwEtmddK9OwDkZQZlgJaOD2-0NXtAtO_jDaG-3VQ#> .

@prefix np: <http://www.nanopub.org/nschema#> .

@prefix dct: <http://purl.org/dc/terms/> .

@prefix nt: <https://w3id.org/np/o/ntemplate/> .

@prefix npx: <http://purl.org/nanopub/x/> .

@prefix xsd: <http://www.w3.org/2001/XMLSchema#> .

@prefix rdfs: <http://www.w3.org/2000/01/rdf-schema#> .

@prefix orcid: <https://orcid.org/> .

@prefix prov: <http://www.w3.org/ns/prov#> .

@prefix sp: <<https://w3id.org/linkflows/superpattern/terms/> .

sub:Head {

this: np:hasAssertion sub:assertion ;

np:hasProvenance sub:provenance ;

np:hasPublicationInfo sub:pubinfo ;

a np:Nanopublication .

}

sub:assertion {

sub:spi a sp:SuperPatternInstance ;

rdfs:label "NGLY1 deficiency is a novel autosomal recessive disorder of the ERAD pathway." ;

sp:hasContextClass <http://www.wikidata.org/entity/Q5> ;

sp:hasSubjectClass <http://purl.obolibrary.org/obo/MONDO_0014109> ;

sp:hasQualifier <https://w3id.org/linkflows/superpattern/terms/alwaysQualifier> ;

sp:hasRelation <https://w3id.org/linkflows/superpattern/terms/isCausedBy> ;

sp:hasObjectClass <http://purl.org/np/RAZVLqlkbwiX40n0GNxcxJany2Cw3oxMCrNuZtjBClryU#Dysfunction_of_ERAD_pathway> .

}

sub:provenance {

sub:activity a sp:FormalizationActivity ;

prov:used sub:quote , <https://dx.doi.org/10.1038%2Fgim.2014.22> ;

prov:wasAssociatedWith orcid:0000-0003-0169-8159 .

sub:assertion prov:wasGeneratedBy sub:activity .

sub:quote prov:value "NGLY1 deficiency is a novel autosomal recessive disorder of the endoplasmic reticulum–associated degradation pathway" ;

prov:wasQuotedFrom <https://dx.doi.org/10.1038%2Fgim.2014.22> .

}

sub:pubinfo {

sub:sig npx:hasAlgorithm "RSA" ;

npx:hasPublicKey "MIGfMA0GCSqGSIb3DQEBAQUAA4GNADCBiQKBgQDOWrvo3T4o7/c9+al6pWVu3tb32/oqIpuNK/bZlFUgR6bnQRBl+7ub5H++34CVFU1pWEQb4RXsx39Q0fM1UPvJbZbXDYAsCEPzn0mzN+YAYdSZKAEyjM2cf4cqjQLwKBabOluVKfKetxTaKB/Jr/9U770dEc2LR3lOMFcbALPSXwIDAQAB" ;

npx:hasSignature "hyYI2msaoxuiMomUFjxJ0K37Cko2r6FQsFper7fJQGC9Y5HMIR//VzDqrzXwz1BGdno3PG8WLv3dvy2vDj+ULXqXxFfp4IuSVekvT902OJXbQuCv40jTVPSgc0LsT0HAll64tIu+nR8o2d8f9lr7mZSCitDc0FsEAcw2U9RUIF4=" ;

npx:hasSignatureTarget this: .

this: dct:created "2021-12-17T10:26:28.040+01:00"^^xsd:dateTime ;

dct:creator orcid:0000-0003-0169-8159 ;

npx:introduces sub:spi ;

npx:supersedes <http://purl.org/np/RASPvPrnUq6SOsOkrgOsUvRbsfmC7dHd73FgUA6oRmlGg> ;

<https://w3id.org/linkflows/reviews/isUpdateOf> <http://purl.org/np/RAGgV-nRMWhmgtxx5n3yE29NaopuuDVDKAuZC8IzCATag> ;

nt:wasCreatedFromProvenanceTemplate <http://purl.org/np/RAE1wniOy0yO39PlK9QkQ-wqbC3q-R2nXraP5huu8W39k> ;

nt:wasCreatedFromPubinfoTemplate <http://purl.org/np/RA2vCBXZf-icEcVRGhulJXugTGxpsV5yVr9yqCI1bQh4A> , <http://purl.org/np/RAA2MfqdBCzmz9yVWjKLXNbyfBNcwsMmOqcNUxkk1maIM> , <http://purl.org/np/RAjpBMlw3owYhJUBo3DtsuDlXsNAJ8cnGeWAutDVjuAuI> ;

nt:wasCreatedFromTemplate <http://purl.org/np/RAv68imZrEjfcp2rnEg1hzoBqEVc0cQMtp9_1Za0BxNM4> .

}

The following nanopublications introduce the newly minted classes in TriG format.

This is the class definition of “dysfunction of ERAD pathway”:

@prefix this: <http://purl.org/np/RAZVLqlkbwiX40n0GNxcxJany2Cw3oxMCrNuZtjBClryU> .

@prefix sub: <http://purl.org/np/RAZVLqlkbwiX40n0GNxcxJany2Cw3oxMCrNuZtjBClryU#> .

@prefix np: <http://www.nanopub.org/nschema#> .

@prefix dct: <http://purl.org/dc/terms/> .

@prefix nt: <https://w3id.org/np/o/ntemplate/> .

@prefix npx: <http://purl.org/nanopub/x/> .

@prefix xsd: <http://www.w3.org/2001/XMLSchema#> .

@prefix rdfs: <http://www.w3.org/2000/01/rdf-schema#> .

@prefix orcid: <https://orcid.org/> .

@prefix prov: <http://www.w3.org/ns/prov#> .

@prefix skos: <http://www.w3.org/2004/02/skos/core#> .

sub:Head {

this: np:hasAssertion sub:assertion ;

np:hasProvenance sub:provenance ;

np:hasPublicationInfo sub:pubinfo ;

a np:Nanopublication .

}

sub:assertion {

sub:Dysfunction_of_ERAD_pathway a <http://www.w3.org/2002/07/owl#Class> ;

rdfs:label "Dysfunction of ERAD pathway" ;

skos:definition "Dysfunction of ERAD pathway" ;

skos:relatedMatch <http://www.wikidata.org/entity/Q21101062> .

}

sub:provenance {

sub:assertion prov:wasAttributedTo orcid:0000-0003-0169-8159 .

}

sub:pubinfo {

sub:sig npx:hasAlgorithm "RSA" ;

npx:hasPublicKey "MIGfMA0GCSqGSIb3DQEBAQUAA4GNADCBiQKBgQDOWrvo3T4o7/c9+al6pWVu3tb32/oqIpuNK/bZlFUgR6bnQRBl+7ub5H++34CVFU1pWEQb4RXsx39Q0fM1UPvJbZbXDYAsCEPzn0mzN+YAYdSZKAEyjM2cf4cqjQLwKBabOluVKfKetxTaKB/Jr/9U770dEc2LR3lOMFcbALPSXwIDAQAB" ;

npx:hasSignature "lHpXoxwEiDwRt0Snb8FuYf1erV7wZgddH0orZakfvel/t2mfo2YjppvSes+/zHlMjU2FxYbUIV7KaitPVbbx1skOGyhbn0mLAB6xNKwy+iA7TjX2NazJVWpHq4EPVXJYsjU3x80TjAWJce6sPbU9gtgHsRGC6fFFGp0rDq70ZP4=" ;

npx:hasSignatureTarget this: .

this: dct:created "2021-12-17T10:19:45.788+01:00"^^xsd:dateTime ;

dct:creator orcid:0000-0003-0169-8159 ;

npx:introduces sub:Dysfunction_of_ERAD_pathway ;

npx:supersedes <http://purl.org/np/RAYXLV7qwfx03AGH7k5ZP6zeMj_vx2kj88z5UQ5OX3C90> ;

nt:wasCreatedFromProvenanceTemplate <http://purl.org/np/RANwQa4ICWS5SOjw7gp99nBpXBasapwtZF1fIM3H2gYTM> ;

nt:wasCreatedFromPubinfoTemplate <http://purl.org/np/RAA2MfqdBCzmz9yVWjKLXNbyfBNcwsMmOqcNUxkk1maIM> , <http://purl.org/np/RAjpBMlw3owYhJUBo3DtsuDlXsNAJ8cnGeWAutDVjuAuI> ;

nt:wasCreatedFromTemplate <http://purl.org/np/RAdpgRpigXtt8iPV9uOPf3wIT3qzOI8Sg2Q72CNV8g-Yo> .

}

**References**

[1] Enns, G., Shashi, V., Bainbridge, M. et al. Mutations in NGLY1 cause an inherited disorder of the endoplasmic reticulum–associated degradation pathway. Genet Med 16, 751–758 (2014). doi: 10.1038/gim.2014.22

[2] Bucur, C.I., Kuhn, T., Ceolin, D., Ossenbruggen, J. van. Expressing high-level scientific claims with formal semantics. In: Proceedings of the 11th Knowledge Capture Conference 2021. doi: 10.1145/3460210.3493561.
