# Supplementary material for: Nanopublication-based semantic publishing and reviewing: a field study with formalization papers
Source: PeerJ Comput Sci. 2023 Feb 21;9:e1159. doi: 10.7717/peerj-cs.1159 (PMC10280262; doi:10.7717/peerj-cs.1159)
Supplement: Supplemental Information 2 [file peerj-cs-09-1159-s002.zip › formalization_papers_supplemental-main/accepted_submissions/s11_Núria_Queralt_Rosinach.pdf]

Title: A formalization of one of the main claims of “Mutations in NGLY1 cause an inherited disorder of the endoplasmic reticulum–associated degradation pathway” by Enns et al. 2014

Authors: Núria Queralt Rosinach, ORCID: 0000-0003-0169-8159

Affiliations: Leiden University Medical Center, Leiden, The Netherlands. E-mail: nqueralt.r@gmail.com

Keywords: “human”, “NGLY1 deficiency”, “dysfunction of ERAD pathway”

Article Type: Formalization Paper

As RDF/nanopublication:
http://purl.org/np/RA12lVwEtmddK9OwDkZQZIgJaOD2-0NXtAtO_jDaG-3VQ

Editor: Cristina-Iulia Bucur, ORCID: 0000-0002-7114-6459

Review comments from:

- Tobias Kuhn, ORCID: 0000-0002-1267-0234
- Cristina-Iulia Bucur, ORCID: 0000-0002-7114-6459

Received: 2021-06-03

Accepted: 2021-12-17

Abstract:
Enns et al. claimed in previous work that NGLY1 deficiency is a novel autosomal recessive disorder of the ERAD pathway. We present here a formalization of that claim, stating that all things of class “NGLY1 deficiency” that are in the context of a thing of class “human” always have a relation of type “is caused by” to a thing of class “dysfunction of ERAD pathway” in the same context.

1. Introduction

Enns et al. [1] state that “NGLY1 deficiency is a novel autosomal recessive disorder of the endoplasmic reticulum–associated degradation pathway.”. We present here a formalization of the main scientific claim from this quote by using a semantic template called the super-pattern [2].

2. Formalization

Our formalization looks as follows:

CONTEXT-CLASS (“in the context of all ..."):	human
SUBJECT-CLASS (“things of type ..."):	NGLY1 deficiency
QUALIFIER:	always
RELATION-TYPE (“have a relation of type...”):	is caused by
OBJECT-CLASS (“to things of type...”):	dysfunction of ERAD pathway

In the context class we use the class “human” (Q5) from Wikidata. In the subject class, we use the class “NGLY1 deficiency” from Mondo Disease Ontology. In the object class we minted a new class “dysfunction of ERAD pathway” that is related to the class “ERAD pathway” (Q21101062) from Wikidata.

3. RDF Code

This is our formalization as a nanopublication in TriG format:

```
@prefix this: <http://purl.org/np/RA12lVwEtmddK9OwDkZQZlgJaOD2-0NXtAtO_jDaG-3VQ> .
@prefix sub: <http://purl.org/np/RA12lVwEtmddK9OwDkZQZlgJaOD2-0NXtAtO_jDaG-3VQ#> .
@prefix np: <http://www.nanopub.org/nschema#> .
@prefix dct: <http://purl.org/dc/terms/> .
@prefix nt: <https://w3id.org/np/o/ntemplate/> .
@prefix npx: <http://purl.org/nanopub/x/> .
@prefix xsd: <http://www.w3.org/2001/XMLSchema#> .
@prefix rdfs: <http://www.w3.org/2000/01/rdf-schema#> .
@prefix orcid: <https://orcid.org/> .
@prefix prov: <http://www.w3.org/ns/prov#> .
@prefix sp: <<https://w3id.org/linkflows/superpattern/terms/> .

sub:Head {
  this: np:hasAssertion sub:assertion ;
    np:hasProvenance sub:provenance ;
    np:hasPublicationInfo sub:pubinfo ;
    a np:Nanopublication .
}
sub:assertion {
  sub:spi a sp:SuperPatternInstance ;
    rdfs:label "NGLY1 deficiency is a novel autosomal recessive disorder of the ERAD pathway." ;
    sp:hasContextClass <http://www.wikidata.org/entity/Q5> ;
    sp:hasSubjectClass <http://purl.obolibrary.org/obo/MONDO_0014109> ;
    sp:hasQualifier <https://w3id.org/linkflows/superpattern/terms/alwaysQualifier> ;
    sp:hasRelation <https://w3id.org/linkflows/superpattern/terms/isCausedBy> ;
    sp:hasObjectClass <http://purl.org/np/RAZVLqlkbwiX40n0GNxcxJany2Cw3oxMCrNuZtjBClryU#Dysfunction_of_ERAD_pathway> .
}
sub:provenance {
  sub:activity a sp:FormalizationActivity ;
    prov:used sub:quote , <https://dx.doi.org/10.1038%2Fgim.2014.22> ;
    prov:wasAssociatedWith orcid:0000-0003-0169-8159 .
  sub:assertion prov:wasGeneratedBy sub:activity .
  sub:quote prov:value "NGLY1 deficiency is a novel autosomal recessive disorder of the endoplasmic reticulum-associated
degradation pathway" ;
    prov:wasQuotedFrom <https://dx.doi.org/10.1038%2Fgim.2014.22> .
}
sub:pubinfo {
  sub:sig npx:hasAlgorithm "RSA" ;
    npx:hasPublicKey
"MIGfMA0GCSqGSIb3DQEBAQUAA4GNADCBiQKBgQDOWrvo3T4o7/c9+al6pWVu3tb32/oqIpuNK/bZlFUgR6bnQRBl+7ub5H++34CVFU1pWEQb4RXsx39Q0fM1UPvJbZbX
DYAsCEPzn0mzN+YAYdSZKAEyjM2cf4cqjQLwKBabOluVKfKetxTaKB/Jr/9U770dEc2LR3lOMFcbALPSXwIDAQAB" ;
    npx:hasSignature
"hyYI2msaoxuiMomUFjxJ0K37Cko2r6FQsFper7fJQGC9Y5HMIR//VzDqrzXwz1BGdno3PG8WLv3dvy2vDj+ULXqXxFfp4IuSVekvT902OJXbQuCv40jTVPSgc0LsT0HA
ll64tIu+nR8o2d8f9lr7mZSCitDc0FsEAcw2U9RUIF4=" ;
    npx:hasSignatureTarget this: .
  this: dct:created "2021-12-17T10:26:28.040+01:00"^^xsd:dateTime ;
    dct:creator orcid:0000-0003-0169-8159 ;
```

```
    npx:introduces sub:spi ;
    npx:supersedes <http://purl.org/np/RASPvPrnUq6SOsOkrgOsUvRbsfmC7dHd73FgUA6oRmlGg> ;
    <https://w3id.org/linkflows/reviews/isUpdateOf> <http://purl.org/np/RAGgV-nRMWhmgtxx5n3yE29NaopuuDVDKAuZC8IzCATag> ;
    nt:wasCreatedFromProvenanceTemplate <http://purl.org/np/RAE1wniOy0yO39PlK9QkQ-wqbC3q-R2nXraP5huu8W39k> ;
    nt:wasCreatedFromPubinfoTemplate <http://purl.org/np/RA2vCBXZf-icEcVRGhulJXugTGxpsV5yVr9yqCI1bQh4A> ,
<http://purl.org/np/RAA2MfqdBCzmz9yVWjKLXNbyfBNcwsMmOqcNUxkk1maIM> ,
<http://purl.org/np/RAjpBMlw3owYhJUBo3DtsuDlXsNAJ8cnGeWAutDVjuAuI> ;
    nt:wasCreatedFromTemplate <http://purl.org/np/RAv68imZrEjfcp2rnEg1hzoBqEVc0cQMtp9_1Za0BxNM4> .
}
```

The following nanopublications introduce the newly minted classes in TriG format.

This is the class definition of “dysfunction of ERAD pathway”:

```
@prefix this: <http://purl.org/np/RAZVLqlkbwiX40n0GNxcxJany2Cw3oxMCrNuZtjBClryU> .
@prefix sub: <http://purl.org/np/RAZVLqlkbwiX40n0GNxcxJany2Cw3oxMCrNuZtjBClryU#> .
@prefix np: <http://www.nanopub.org/nschema#> .
@prefix dct: <http://purl.org/dc/terms/> .
@prefix nt: <https://w3id.org/np/o/ntemplate/> .
@prefix npx: <http://purl.org/nanopub/x/> .
@prefix xsd: <http://www.w3.org/2001/XMLSchema#> .
@prefix rdfs: <http://www.w3.org/2000/01/rdf-schema#> .
@prefix orcid: <https://orcid.org/> .
@prefix prov: <http://www.w3.org/ns/prov#> .
@prefix skos: <http://www.w3.org/2004/02/skos/core#> .

sub:Head {
  this: np:hasAssertion sub:assertion ;
    np:hasProvenance sub:provenance ;
    np:hasPublicationInfo sub:pubinfo ;
    a np:Nanopublication .
}
sub:assertion {
  sub:Dysfunction_of_ERAD_pathway a <http://www.w3.org/2002/07/owl#Class> ;
    rdfs:label "Dysfunction of ERAD pathway" ;
    skos:definition "Dysfunction of ERAD pathway" ;
    skos:relatedMatch <http://www.wikidata.org/entity/Q21101062> .
}
sub:provenance {
  sub:assertion prov:wasAttributedTo orcid:0000-0003-0169-8159 .
}
sub:pubinfo {
  sub:sig npx:hasAlgorithm "RSA" ;
    npx:hasPublicKey
"MIGfMA0GCSqGSIb3DQEBAQUAA4GNADCBiQKBgQDOWrvo3T4o7/c9+al6pWVu3tb32/oqIpuNK/bZlFUgR6bnQRBl+7ub5H++34CVFU1pWEQb4RXsx39Q0fM1UPvJbZbX
DYAsCEPzn0mzN+YAYdSZKAEyjM2cf4cqjQLwKBabOluVKfKetxTaKB/Jr/9U770dEc2LR3lOMFcbALPSXwIDAQAB" ;
    npx:hasSignature
"lHpXoxwEiDwRt0Snb8FuYf1erV7wZgddH0orZakfvel/t2mfo2YjppvSes+/zHlMjU2FxYbUIV7KaitPVbbx1skOGyhbn0mLAB6xNKwy+iA7TjX2NazJVWpHq4EPVXJY
sjU3x80TjAWJce6sPbU9gtgHsRGC6fFFGp0rDq70ZP4=" ;
    npx:hasSignatureTarget this: .
  this: dct:created "2021-12-17T10:19:45.788+01:00"^^xsd:dateTime ;
    dct:creator orcid:0000-0003-0169-8159 ;
    npx:introduces sub:Dysfunction_of_ERAD_pathway ;
    npx:supersedes <http://purl.org/np/RAYXLV7qwfx03AGH7k5ZP6zeMj_vx2kj88z5UQ5OX3C90> ;
    nt:wasCreatedFromProvenanceTemplate <http://purl.org/np/RANwQa4ICWS5SOjw7gp99nBpXBasapwtZF1fIM3H2gYTM> ;
    nt:wasCreatedFromPubinfoTemplate <http://purl.org/np/RAA2MfqdBCzmz9yVWjKLXNbyfBNcwsMmOqcNUxkk1maIM> ,
<http://purl.org/np/RAjpBMlw3owYhJUBo3DtsuDlXsNAJ8cnGeWAutDVjuAuI> ;
    nt:wasCreatedFromTemplate <http://purl.org/np/RAdpgRpigXtt8iPV9uOPf3wIT3qzOI8Sg2Q72CNV8g-Yo> .
}
```

References

[1] Enns, G., Shashi, V., Bainbridge, M. et al. Mutations in NGLY1 cause an inherited disorder of the endoplasmic reticulum–associated degradation pathway. Genet Med 16, 751–758 (2014). doi: 10.1038/gim.2014.22

[2] Bucur, C.I., Kuhn, T., Ceolin, D., Ossenbruggen, J. van. Expressing high-level scientific claims with formal semantics. In: Proceedings of the 11th Knowledge Capture Conference 2021. doi: 10.1145/3460210.3493561.
