# Supplementary material for: Nanopublication-based semantic publishing and reviewing: a field study with formalization papers
Source: PeerJ Comput Sci. 2023 Feb 21;9:e1159. doi: 10.7717/peerj-cs.1159 (PMC10280262; doi:10.7717/peerj-cs.1159)
Supplement: Supplemental Information 2 [file peerj-cs-09-1159-s002.zip › formalization_papers_supplemental-main/accepted_submissions/s12_Ricardo_Usbeck.pdf]

Title: A formalization of one of the main claims of "'Dunbar's number' deconstructed" by Lindenfors et al. 2021

Authors: Ricardo Usbeck, ORCID: 0000-0002-0191-7211

Affiliations: University of Hamburg, Germany. E-mail: ricardo.usbeck@uni-hamburg.de , ricardo.usbeck@googlemail.com

Keywords: "social group", "relative neocortex size", "social group size"

Article Type: Formalization Paper

As RDF/nanopublication:
http://purl.org/np/RAbWbJCYlLhlYBDn9PVxdJP_WUbbi058aRcK-3sOJsRwY

Editor: Cristina-Iulia Bucur, ORCID: 0000-0002-7114-6459

Review comments from:
- Tobias Kuhn, ORCID: 0000-0002-1267-0234
- Michel Dumontier, ORCID: 0000-0003-4727-9435
- Cristina-Iulia Bucur, ORCID: 0000-0002-7114-6459

Received: 2021-06-22

Accepted: 2021-11-17

Abstract:
Lindenfors et al. claimed in previous work that the cortex size of humans does not relate to their social group size. We present here a formalization of that claim, stating that all things of class "relative neocortex size" that are in the context of a thing of class "social group" never have a relation of type "affects" to a thing of class "social group size" in the same context.

1. Introduction

Lindenfors et al. [1] state that "A cognitive limit on human group size cannot be derived in this manner.". We present here a formalization of the main scientific claim from this quote by using a semantic template called the super-pattern [2].

2. Formalization

Our formalization looks as follows:

CONTEXT-CLASS ("in the context of all ..."): social group

SUBJECT-CLASS (“things of type ..."):	relative neocortex size
QUALIFIER:	never
RELATION-TYPE (“have a relation of type...”):	affects
OBJECT-CLASS (“to things of type...”):	social group size

In the context class, we use “social group” (Q874405) from Wikidata. In the subject class, we use a new minted class “relative neocortex size” that is related to the class “size”(Q1152227) and “neocortex” (Q726562) from Wikidata. In the object class, we minted a new class “social group size” that is a subclass of “social group” (Q874405) from Wikidata.

3. RDF Code

This is our formalization as a nanopublication in TriG format:

```
@prefix this: <http://purl.org/np/RAbWbJCYlLhlYBDn9PVxdJP_WUbbi058aRcK-3sOJsRwY> .
@prefix sub: <http://purl.org/np/RAbWbJCYlLhlYBDn9PVxdJP_WUbbi058aRcK-3sOJsRwY#> .
@prefix np: <http://www.nanopub.org/nschema#> .
@prefix dct: <http://purl.org/dc/terms/> .
@prefix nt: <https://w3id.org/np/o/ntemplate/> .
@prefix npx: <http://purl.org/nanopub/x/> .
@prefix xsd: <http://www.w3.org/2001/XMLSchema#> .
@prefix rdfs: <http://www.w3.org/2000/01/rdf-schema#> .
@prefix orcid: <https://orcid.org/> .
@prefix prov: <http://www.w3.org/ns/prov#> .
@prefix sp: <https://w3id.org/linkflows/superpattern/terms/> .

sub:Head {
  this: np:hasAssertion sub:assertion ;
    np:hasProvenance sub:provenance ;
    np:hasPublicationInfo sub:pubinfo ;
    a np:Nanopublication .
}
sub:assertion {
  sub:spi a sp:SuperPatternInstance ;
    rdfs:label "The cortex size of humans does not relate to their social group size" ;
    sp:hasContextClass <http://www.wikidata.org/entity/Q874405> ;
    sp:hasSubjectClass <http://purl.org/np/RAhnnsMWVM8M29NixCJfVDLWzRzwwCPnUD7LI2kxT-FME#relative-neocortex-size> ;
    sp:hasQualifier sp:neverQualifier ;
    sp:hasRelation sp:affects ;
    sp:hasObjectClass <http://purl.org/np/RAlKYv_sE8qwiSqsRdcr7KrkU1bsqlqiFmhDPtPBwpLrM#social-group-size> .
}
sub:provenance {
  sub:activity a sp:FormalizationActivity ;
    prov:used sub:quote , <https://royalsocietypublishing.org/doi/10.1098/rsbl.2021.0158> ;
    prov:wasAssociatedWith orcid:0000-0002-0191-7211 .
  sub:assertion prov:wasGeneratedBy sub:activity .
  sub:quote prov:value "A cognitive limit on human group size cannot be derived in this manner." ;
    prov:wasQuotedFrom <https://royalsocietypublishing.org/doi/10.1098/rsbl.2021.0158> .
}
sub:pubinfo {
  sub:sig npx:hasAlgorithm "RSA" ;
    npx:hasPublicKey
"MIGfMA0GCSqGSIb3DQEBAQUAA4GNADCBiQKBgQC3cICbOHZ1ecuLOsgovKwUlFRRvNWNgBJxoXCFAYWtU2OK97i/SjoIaxFphg9tkv9WxOjtPqYJ4cuM0E76wxeRZ7VB
MHBrBIyCAGTvAfESWVQZCDgulG46VjffEzXuRmqOka/C5Ur6beLijFPMtiWEg6I2Mbj8z9vuHPxReIu4JwIDAQAB" ;
    npx:hasSignature
"ZFigeQHif/2IZT/QsVTqDThjV1P0Uy4IkGEmOC8W/Iygn8sGpJt4DOBDLDrT2egAwiesYqFxVqSDQnWCeY2C/yaVIob6j49CYlZor+VZQo1HdsGvEq3V7xiLobl/w4sM
PxA690y9DF5zp8w3wggXaJxMjrAESSCpaPxtnKGhxt0=" ;
    npx:hasSignatureTarget this: .
  this: dct:created "2021-11-17T18:03:20.686+01:00"^^xsd:dateTime ;
    dct:creator orcid:0000-0002-0191-7211 ;
    npx:introduces sub:spi ;
    npx:supersedes <http://purl.org/np/RA7MgxMCcVykE2RBJHm-Ou_CuuhPXqfcinswtIfjXReOc> ;
    <https://w3id.org/linkflows/reviews/isUpdateOf> <http://purl.org/np/RAYJe1ruxzvy8idQGuxziYN4ri8OFeKZxzK-QdJPvDug0> ;
```

```
    nt:wasCreatedFromProvenanceTemplate <http://purl.org/np/RAB_oy10D3XUP-zYlqGz7Uj58AsUXhEKeGqmRFg5LSgDM> ;
    nt:wasCreatedFromPubinfoTemplate <http://purl.org/np/RA2vCBXZf-icEcVRGhulJXugTGxpsV5yVr9yqCI1bQh4A> ,
<http://purl.org/np/RAA2MfqdBCzmz9yVWjKLXNbyfBNcwsMmOqcNUxkk1maIM> ,
<http://purl.org/np/RAjpBMlw3owYhJUBo3DtsuDlXsNAJ8cnGeWAutDVjuAuI> ;
    nt:wasCreatedFromTemplate <http://purl.org/np/RAv68imZrEjfcp2rnEg1hzoBqEVc0cQMtp9_1Za0BxNM4> .
}
```

The following nanopublications introduce the newly minted classes in TriG format.

This is the class definition of “relative neocortex size”:

```
@prefix this: <http://purl.org/np/RAhnnsMWVM8M29NixCJfVDLWzRzwwCPnUD7LI2kxT-FME> .
@prefix sub: <http://purl.org/np/RAhnnsMWVM8M29NixCJfVDLWzRzwwCPnUD7LI2kxT-FME#> .
@prefix np: <http://www.nanopub.org/nschema#> .
@prefix dct: <http://purl.org/dc/terms/> .
@prefix nt: <https://w3id.org/np/o/ntemplate/> .
@prefix npx: <http://purl.org/nanopub/x/> .
@prefix xsd: <http://www.w3.org/2001/XMLSchema#> .
@prefix rdfs: <http://www.w3.org/2000/01/rdf-schema#> .
@prefix orcid: <https://orcid.org/> .
@prefix prov: <http://www.w3.org/ns/prov#> .
@prefix skos: <http://www.w3.org/2004/02/skos/core#> .

sub:Head {
  this: np:hasAssertion sub:assertion ;
    np:hasProvenance sub:provenance ;
    np:hasPublicationInfo sub:pubinfo ;
    a np:Nanopublication .
}
sub:assertion {
  sub:relative-neocortex-size a <http://www.w3.org/2002/07/owl#Class> ;
    rdfs:label "Relative size of the neocortex" ;
    skos:definition "This class signifies the weight or volume of a part of the brain called neocortex." ;
    skos:relatedMatch <http://www.wikidata.org/entity/Q322481> , <http://www.wikidata.org/entity/Q726562> .
}
sub:provenance {
  sub:assertion prov:wasAttributedTo orcid:0000-0002-0191-7211 .
}
sub:pubinfo {
  sub:sig npx:hasAlgorithm "RSA" ;
    npx:hasPublicKey
"MIGfMA0GCSqGSIb3DQEBAQUAA4GNADCBiQKBgQC3cICbOHZ1ecuLOsgovKwUlFRRvNWNgBJxoXCFAYWtU2OK97i/SjoIaxFphg9tkv9WxOjtPqYJ4cuM0E76wxeRZ7VB
MHBrBIyCAGTvAfESWVQZCDgulG46VjffEzXuRmqOka/C5Ur6beLijFPMtiWEg6I2Mbj8z9vuHPxReIu4JwIDAQAB" ;
    npx:hasSignature
"NMWYJ3I459BEnN7NgKELsfNucMayG4MXMMjOrZm0h+g/5Mbu8vbLVhkSi5C9N+SGc67qW6YtdndVxrMS7qpWfzUTv+FzVOgydGqs30VKUC5F0TNBzvTnTNvT9Bq61oQ5
cNIxUHef6/Mqte2auchZPLgY8yap3ImLEaNTWwdQpTM=" ;
    npx:hasSignatureTarget this: .
  this: dct:created "2021-10-19T14:07:44.194+02:00"^^xsd:dateTime ;
    dct:creator orcid:0000-0002-0191-7211 ;
    npx:introduces sub:relative-neocortex-size ;
    npx:supersedes <http://purl.org/np/RAIwMZ0StXU9DCY1WGoJMwD2NAc30mijCb0FML8TXIB0Y> ;
    <https://w3id.org/linkflows/reviews/isUpdateOf> <http://purl.org/np/RAIwMZ0StXU9DCY1WGoJMwD2NAc30mijCb0FML8TXIB0Y> ;
    nt:wasCreatedFromProvenanceTemplate <http://purl.org/np/RANwQa4ICWS5SOjw7gp99nBpXBasapwtZF1fIM3H2gYTM> ;
    nt:wasCreatedFromPubinfoTemplate <http://purl.org/np/RAA2MfqdBCzmz9yVWjKLXNbyfBNcwsMmOqcNUxkk1maIM> ,
<http://purl.org/np/RAOGu9Lh0BD4tbIRB9RG6RGRA_ObDh75NTbIqaWgxxs8M> ,
<http://purl.org/np/RAjpBMlw3owYhJUBo3DtsuDlXsNAJ8cnGeWAutDVjuAuI> ;
    nt:wasCreatedFromTemplate <http://purl.org/np/RAdpgRpigXtt8iPV9uOPf3wIT3qzOI8Sg2Q72CNV8g-Yo> .
}
```

This is the class definition of “social group size”:

```
@prefix this: <http://purl.org/np/RAlKYv_sE8qwiSqsRdcr7KrkU1bsqlqiFmhDPtPBwpLrM> .
@prefix sub: <http://purl.org/np/RAlKYv_sE8qwiSqsRdcr7KrkU1bsqlqiFmhDPtPBwpLrM#> .
@prefix np: <http://www.nanopub.org/nschema#> .
@prefix dct: <http://purl.org/dc/terms/> .
@prefix nt: <https://w3id.org/np/o/ntemplate/> .
@prefix npx: <http://purl.org/nanopub/x/> .
@prefix xsd: <http://www.w3.org/2001/XMLSchema#> .
@prefix rdfs: <http://www.w3.org/2000/01/rdf-schema#> .
@prefix orcid: <https://orcid.org/> .
@prefix prov: <http://www.w3.org/ns/prov#> .
@prefix skos: <http://www.w3.org/2004/02/skos/core#> .

sub:Head {
  this: np:hasAssertion sub:assertion ;
```

```
    np:hasProvenance sub:provenance ;
    np:hasPublicationInfo sub:pubinfo ;
    a np:Nanopublication .
}
sub:assertion {
  sub:social-group-size a <http://www.w3.org/2002/07/owl#Class> ;
    rdfs:label "Social group size" ;
    rdfs:subClassOf <https://www.wikidata.org/wiki/Q874405> ;
    skos:definition "Social group size is the size of a group of humans that a person socially interacts with (on a regular
basis)." .
}
sub:provenance {
  sub:assertion prov:wasAttributedTo orcid:0000-0002-0191-7211 .
}
sub:pubinfo {
  sub:sig npx:hasAlgorithm "RSA" ;
    npx:hasPublicKey
"MIGfMA0GCSqGSIb3DQEBAQUAA4GNADCBiQKBgQC3cICbOHZ1ecuLOsgovKwUlFRRvNWNgBJxoXCFAYWtU2OK97i/SjoIaxFphg9tkv9WxOjtPqYJ4cuM0E76wxeRZ7VB
MHBrBIyCAGTvAfESWVQZCDgulG46VjffEzXuRmqOka/C5Ur6beLijFPMtiWEg6I2Mbj8z9vuHPxReIu4JwIDAQAB" ;
    npx:hasSignature
"WBZ10x+hSX0zXoXQAGBkM89SWDOnViMI8NoLH8w1GsjOWSjT/pasNugo6CEDZkWb5ZyHsXSSzG7JDCldnhVQkH64Nuok6C3QvV9+EUVMZaDHByMhObzJ3Pld8pYNHWtW
1wNve4xkb91Mzq5zYfBw+Y4ufB5Kqi39zH1dr6XRZRo=" ;
    npx:hasSignatureTarget this: .
  this: dct:created "2021-06-22T09:49:42.152Z"^^xsd:dateTime ;
    dct:creator orcid:0000-0002-0191-7211 ;
    npx:introduces sub:social-group-size ;
    nt:wasCreatedFromProvenanceTemplate <http://purl.org/np/RANwQa4ICWS5SOjw7gp99nBpXBasapwtZF1fIM3H2gYTM> ;
    nt:wasCreatedFromPubinfoTemplate <http://purl.org/np/RAA2MfqdBCzmz9yVWjKLXNbyfBNcwsMmOqcNUxkk1maIM> ;
    nt:wasCreatedFromTemplate <http://purl.org/np/RAdpgRpigXtt8iPV9uOPf3wIT3qzOI8Sg2Q72CNV8g-Yo> .
}
```

References

[1] Lindenfors, P., Wartel, A. and Lind, J. ‘Dunbar's number’ deconstructed. Biol. Lett.(2021). doi: 10.1098/rsbl.2021.0158.
[2] Bucur, C.I., Kuhn, T., Ceolin, D., Ossenbruggen, J. van. Expressing high-level scientific claims with formal semantics. In: Proceedings of the 11th Knowledge Capture Conference 2021. doi: 10.1145/3460210.3493561.
