# Supplementary material for: Nanopublication-based semantic publishing and reviewing: a field study with formalization papers
Source: PeerJ Comput Sci. 2023 Feb 21;9:e1159. doi: 10.7717/peerj-cs.1159 (PMC10280262; doi:10.7717/peerj-cs.1159)
Supplement: Supplemental Information 2 [file peerj-cs-09-1159-s002.zip › formalization_papers_supplemental-main/accepted_submissions/s13_Russell_Bainer.docx]

**Title:** A formalization of one of the main claims of “The cancer glycocalyx mechanically primes integrin-mediated growth and survival” by Paszek et al. 2014

**Authors:** Russell Bainer, ORCID: 0000-0002-0830-7029

**Affiliations:** Maze Therapeutics, USA. E-mail: [rbainer@mazetx.com](mailto:rbainer@mazetx.com)

**Keywords:** “ecm bound cancer cell”, “glycocalyx bulk”, “integrin clustering”

**Article Type:** Formalization Paper

**As RDF/nanopublication:** <http://purl.org/np/RAoo8EvTgfkxJw5SgZXbJvRl5nQG7ygeGaHp8Zud1U4Zw>

**Editor:** Cristina-Iulia Bucur, ORCID: 0000-0002-7114-6459

**Review comments from:**

- Tobias Kuhn, ORCID: 0000-0002-1267-0234
- Michel Dumontier, ORCID: 0000-0003-4727-9435
- Margherita Martorana, ORCID: 0000-0001-8004-0464
- Cristina-Iulia Bucur, ORCID: 0000-0002-7114-6459

**Received:** 2021-06-18

**Accepted:** 2021-11-29

**Abstract:**

Paszek et al. claimed in previous work that glycocalyx bulk mechanically drives integrin clustering in cancer cells engaged with the extracellular matrix. We present here a formalization of that claim, stating that all things of class “glycocalyx bulk” that are in the context of a thing of class “ecm bound cancer cell” generally have a relation of type “increases” to a thing of class “integrin clustering” in the same context.

1. **Introduction**

Paszek et al. [1] state that “Expression of large tumour-associated glycoproteins in non-transformed mammary cells promoted focal adhesion assembly and facilitated integrin-dependent growth factor signalling to support cell growth and survival.”. We present here a formalization of the main scientific claim from this quote by using a semantic template called the super-pattern [2].

1. **Formalization**

Our formalization looks as follows:

| CONTEXT-CLASS (“in the context of all ..."): | [ecm bound cancer cell](http://purl.org/np/RAaOAF90U6YxAvnchfj0dRtT5HRz320Pz202aGap-VfuI#ecm-bound-cancer-cell) |
| --- | --- |
| SUBJECT-CLASS (“things of type ..."): | [glycocalyx bulk](http://purl.org/np/RA-jkb7qPNTSOe_EXltW_rlQWQ9x3_Y1KOzW6J_bbPz4U#glycocalyx-bulk) |
| QUALIFIER: | [generally](https://w3id.org/linkflows/superpattern/terms/generallyQualifier) |
| RELATION-TYPE (“have a relation of type...”): | [increases](https://w3id.org/linkflows/superpattern/terms/increases) |
| OBJECT-CLASS (“to things of type...”): | [integrin clustering](http://purl.org/np/RAFH8AVn-wnTcSGxvPZ1Uiy_AtOhINlynnAxxiCdcTVWU#integrin-clustering) |

In the context class we use a new minted class “ecm bound cancer cell” that is a subclass of “cancer cell” (Q4118072) from Wikidata and is related to the class “extracellular matrix” (Q193825) from Wikidata. In the subject class, we use a new minted class “glycocalyx bulk” that is related to the class “glycocalyx” (Q898356) from Wikidata. In the object class we minted a new class “integrin clustering” that is a subclass of “integrin binding” (Q14633861) from Wikidata and is related to the class “focal adhesion” (Q904514) from Wikidata.

1. **RDF Code**

This is our formalization as a nanopublication in TriG format:

@prefix this: <http://purl.org/np/RAoo8EvTgfkxJw5SgZXbJvRl5nQG7ygeGaHp8Zud1U4Zw> .

@prefix sub: <http://purl.org/np/RAoo8EvTgfkxJw5SgZXbJvRl5nQG7ygeGaHp8Zud1U4Zw#> .

@prefix np: <http://www.nanopub.org/nschema#> .

@prefix dct: <http://purl.org/dc/terms/> .

@prefix nt: <https://w3id.org/np/o/ntemplate/> .

@prefix npx: <http://purl.org/nanopub/x/> .

@prefix xsd: <http://www.w3.org/2001/XMLSchema#> .

@prefix rdfs: <http://www.w3.org/2000/01/rdf-schema#> .

@prefix orcid: <https://orcid.org/> .

@prefix prov: <http://www.w3.org/ns/prov#> .

@prefix sp: <https://w3id.org/linkflows/superpattern/terms/> .

sub:Head {

this: np:hasAssertion sub:assertion ;

np:hasProvenance sub:provenance ;

np:hasPublicationInfo sub:pubinfo ;

a np:Nanopublication .

}

sub:assertion {

sub:spi a sp:SuperPatternInstance ;

rdfs:label "Glycocalyx bulk mechanically drives integrin clustering in cancer cells engaged with the extracellular matrix" ;

sp:hasContextClass <http://purl.org/np/RAaOAF90U6YxAvnchfj0dRtT5HRz320Pz202aGap-VfuI#ecm-bound-cancer-cell> ;

sp:hasSubjectClass <http://purl.org/np/RA-jkb7qPNTSOe_EXltW_rlQWQ9x3_Y1KOzW6J_bbPz4U#glycocalyx-bulk> ;

sp:hasQualifier <https://w3id.org/linkflows/superpattern/terms/generallyQualifier> ;

sp:hasRelation <https://w3id.org/linkflows/superpattern/terms/increases> ;

sp:hasObjectClass <http://purl.org/np/RAFH8AVn-wnTcSGxvPZ1Uiy_AtOhINlynnAxxiCdcTVWU#integrin-clustering> .

}

sub:provenance {

sub:activity a sp:FormalizationActivity ;

prov:used sub:quote , <https://www.nature.com/articles/nature13535> ;

prov:wasAssociatedWith orcid:0000-0002-0830-7029 .

sub:assertion prov:wasGeneratedBy sub:activity .

sub:quote prov:value "Expression of large tumour-associated glycoproteins in non-transformed mammary cells promoted focal adhesion assembly and facilitated integrin-dependent growth factor signalling to support cell growth and survival." ;

prov:wasQuotedFrom <https://www.nature.com/articles/nature13535> .

}

sub:pubinfo {

sub:sig npx:hasAlgorithm "RSA" ;

npx:hasPublicKey "MIGfMA0GCSqGSIb3DQEBAQUAA4GNADCBiQKBgQCluZ2msgo7OqPkyRoZMluzpaLX8KRHyDs3J/cZwm6+Vq/CKVIGsGaT7/XH435cE9J8dwCgQ/Jssjlj6oqX+nSI9xXBtMH9ZeyGHFBzK1cUBy/rRSez4EnwbS1sKMBSc4MBCXQ9R3jZ3GpsN0GmIrS1ukKkUBQrREBYpb4w5MbGywIDAQAB" ;

npx:hasSignature "Mc6yNlLGIu3AnhZEmrajdDXbYISe2imFa8OBDfBIWbQvJlOdxKaohx+dR9jwbzecksZ+bMNvtojD/ftMxBeLu3126L1DmW0kxIWoTvfi+RRhgeaXTkRT/RV7EBmcuEfzEU6Lk3YkK/v4dOiCP24mei1YLdsNRLK4n44AvOr54lE=" ;

npx:hasSignatureTarget this: .

this: dct:created "2021-11-26T17:14:38.341-07:00"^^xsd:dateTime ;

dct:creator orcid:0000-0002-0830-7029 ;

npx:introduces sub:spi ;

npx:supersedes <http://purl.org/np/RAh1GOK4_HhqeUeJhIyV_DDCFFTY9jeeiKWgbSzooWEi4> ;

<https://w3id.org/linkflows/reviews/isUpdateOf> <http://purl.org/np/RASZZ5T1Ca5gpCMPubKoypR_0WcSUkHiMaADu9o1BS1Xs> ;

nt:wasCreatedFromProvenanceTemplate <http://purl.org/np/RAE1wniOy0yO39PlK9QkQ-wqbC3q-R2nXraP5huu8W39k> ;

nt:wasCreatedFromPubinfoTemplate <http://purl.org/np/RA2vCBXZf-icEcVRGhulJXugTGxpsV5yVr9yqCI1bQh4A> , <http://purl.org/np/RAA2MfqdBCzmz9yVWjKLXNbyfBNcwsMmOqcNUxkk1maIM> , <http://purl.org/np/RAjpBMlw3owYhJUBo3DtsuDlXsNAJ8cnGeWAutDVjuAuI> ;

nt:wasCreatedFromTemplate <http://purl.org/np/RAv68imZrEjfcp2rnEg1hzoBqEVc0cQMtp9_1Za0BxNM4> .

}

The following nanopublications introduce the newly minted classes in TriG format.

This is the class definition of “ecm bound cancer cell”:

@prefix this: <http://purl.org/np/RAaOAF90U6YxAvnchfj0dRtT5HRz320Pz202aGap-VfuI> .

@prefix sub: <http://purl.org/np/RAaOAF90U6YxAvnchfj0dRtT5HRz320Pz202aGap-VfuI#> .

@prefix np: <http://www.nanopub.org/nschema#> .

@prefix dct: <http://purl.org/dc/terms/> .

@prefix nt: <https://w3id.org/np/o/ntemplate/> .

@prefix npx: <http://purl.org/nanopub/x/> .

@prefix xsd: <http://www.w3.org/2001/XMLSchema#> .

@prefix rdfs: <http://www.w3.org/2000/01/rdf-schema#> .

@prefix orcid: <https://orcid.org/> .

@prefix prov: <http://www.w3.org/ns/prov#> .

@prefix skos: <http://www.w3.org/2004/02/skos/core#> .

sub:Head {

this: np:hasAssertion sub:assertion ;

np:hasProvenance sub:provenance ;

np:hasPublicationInfo sub:pubinfo ;

a np:Nanopublication .

}

sub:assertion {

sub:ecm-bound-cancer-cell a <http://www.w3.org/2002/07/owl#Class> ;

rdfs:label "cancer cell engaged in extracellular matrix" ;

rdfs:subClassOf <http://www.wikidata.org/entity/Q4118072> ;

skos:definition "cancer cell engaged in extracellular matrix" ;

skos:relatedMatch <http://www.wikidata.org/entity/Q193825> .

}

sub:provenance {

sub:assertion prov:wasAttributedTo orcid:0000-0002-0830-7029 .

}

sub:pubinfo {

sub:sig npx:hasAlgorithm "RSA" ;

npx:hasPublicKey "MIGfMA0GCSqGSIb3DQEBAQUAA4GNADCBiQKBgQCluZ2msgo7OqPkyRoZMluzpaLX8KRHyDs3J/cZwm6+Vq/CKVIGsGaT7/XH435cE9J8dwCgQ/Jssjlj6oqX+nSI9xXBtMH9ZeyGHFBzK1cUBy/rRSez4EnwbS1sKMBSc4MBCXQ9R3jZ3GpsN0GmIrS1ukKkUBQrREBYpb4w5MbGywIDAQAB" ;

npx:hasSignature "J5J3Blgx/OmReiNFC8+4TzqHm34UUmKcQKRhq7SVPq5H0rLE0fqyX4RfZfA0oQg5dfD7vnbVttaGsUQZs1Iw1dU4r/CYko2IXUQiptdcY28cR76t/4coiBcFCZpqy3GPTKwIj7uR/aJIUj4UDzA6LCmsWju0hT1bp5NDmNhT+8E=" ;

npx:hasSignatureTarget this: .

this: dct:created "2021-11-12T09:21:35.816-08:00"^^xsd:dateTime ;

dct:creator orcid:0000-0002-0830-7029 ;

npx:introduces sub:ecm-bound-cancer-cell ;

npx:supersedes <http://purl.org/np/RAXecDWH8gZVp7FZRP1m_Fg6EXH_uoZGhKN9blfN9lrfI> ;

<https://w3id.org/linkflows/reviews/isUpdateOf> <http://purl.org/np/RAXecDWH8gZVp7FZRP1m_Fg6EXH_uoZGhKN9blfN9lrfI> ;

nt:wasCreatedFromProvenanceTemplate <http://purl.org/np/RANwQa4ICWS5SOjw7gp99nBpXBasapwtZF1fIM3H2gYTM> ;

nt:wasCreatedFromPubinfoTemplate <http://purl.org/np/RAA2MfqdBCzmz9yVWjKLXNbyfBNcwsMmOqcNUxkk1maIM> , <http://purl.org/np/RAOGu9Lh0BD4tbIRB9RG6RGRA_ObDh75NTbIqaWgxxs8M> , <http://purl.org/np/RAjpBMlw3owYhJUBo3DtsuDlXsNAJ8cnGeWAutDVjuAuI> ;

nt:wasCreatedFromTemplate <http://purl.org/np/RAdpgRpigXtt8iPV9uOPf3wIT3qzOI8Sg2Q72CNV8g-Yo> .

}

This is the class definition of “glycocalyx bulk”:

@prefix this: <http://purl.org/np/RA-jkb7qPNTSOe_EXltW_rlQWQ9x3_Y1KOzW6J_bbPz4U> .

@prefix sub: <http://purl.org/np/RA-jkb7qPNTSOe_EXltW_rlQWQ9x3_Y1KOzW6J_bbPz4U#> .

@prefix np: <http://www.nanopub.org/nschema#> .

@prefix dct: <http://purl.org/dc/terms/> .

@prefix nt: <https://w3id.org/np/o/ntemplate/> .

@prefix npx: <http://purl.org/nanopub/x/> .

@prefix xsd: <http://www.w3.org/2001/XMLSchema#> .

@prefix rdfs: <http://www.w3.org/2000/01/rdf-schema#> .

@prefix orcid: <https://orcid.org/> .

@prefix prov: <http://www.w3.org/ns/prov#> .

@prefix skos: <http://www.w3.org/2004/02/skos/core#> .

sub:Head {

this: np:hasAssertion sub:assertion ;

np:hasProvenance sub:provenance ;

np:hasPublicationInfo sub:pubinfo ;

a np:Nanopublication .

}

sub:assertion {

sub:glycocalyx-bulk a <http://www.w3.org/2002/07/owl#Class> ;

rdfs:label "glycocalyx bulk" ;

skos:definition "Relating to size, density, or bulk of the glycocalyx" ;

skos:relatedMatch <http://www.wikidata.org/entity/Q898356> .

}

sub:provenance {

sub:assertion prov:wasAttributedTo orcid:0000-0002-0830-7029 .

}

sub:pubinfo {

sub:sig npx:hasAlgorithm "RSA" ;

npx:hasPublicKey "MIGfMA0GCSqGSIb3DQEBAQUAA4GNADCBiQKBgQCluZ2msgo7OqPkyRoZMluzpaLX8KRHyDs3J/cZwm6+Vq/CKVIGsGaT7/XH435cE9J8dwCgQ/Jssjlj6oqX+nSI9xXBtMH9ZeyGHFBzK1cUBy/rRSez4EnwbS1sKMBSc4MBCXQ9R3jZ3GpsN0GmIrS1ukKkUBQrREBYpb4w5MbGywIDAQAB" ;

npx:hasSignature "WlbAvo5qWbVaY31i3E/uayLdSkt0IFbe2m+P7z0CEaoghb+sTAehmDjw9pmd2bzPQFblGdIwBr5yhGHXGorM4v3bkV2nIiRecOwhvtu9hM2pIUtS8QwD6kggHsO1WxzT4Wqs3GmS63PgN3cxZjEs0BuTWUhiXbd2V6gkLUPvaK8=" ;

npx:hasSignatureTarget this: .

this: dct:created "2021-11-12T09:27:02.246-08:00"^^xsd:dateTime ;

dct:creator orcid:0000-0002-0830-7029 ;

npx:introduces sub:glycocalyx-bulk ;

npx:supersedes <http://purl.org/np/RACS5sSm45MGbuJrztnPrpdu1AuLiKxDni-6JcKzN5ZtM> ;

<https://w3id.org/linkflows/reviews/isUpdateOf> <http://purl.org/np/RACS5sSm45MGbuJrztnPrpdu1AuLiKxDni-6JcKzN5ZtM> ;

nt:wasCreatedFromProvenanceTemplate <http://purl.org/np/RANwQa4ICWS5SOjw7gp99nBpXBasapwtZF1fIM3H2gYTM> ;

nt:wasCreatedFromPubinfoTemplate <http://purl.org/np/RAA2MfqdBCzmz9yVWjKLXNbyfBNcwsMmOqcNUxkk1maIM> , <http://purl.org/np/RAOGu9Lh0BD4tbIRB9RG6RGRA_ObDh75NTbIqaWgxxs8M> , <http://purl.org/np/RAjpBMlw3owYhJUBo3DtsuDlXsNAJ8cnGeWAutDVjuAuI> ;

nt:wasCreatedFromTemplate <http://purl.org/np/RAdpgRpigXtt8iPV9uOPf3wIT3qzOI8Sg2Q72CNV8g-Yo> .

}

This is the class definition of “integrin clustering”:

@prefix this: <http://purl.org/np/RAFH8AVn-wnTcSGxvPZ1Uiy_AtOhINlynnAxxiCdcTVWU> .

@prefix sub: <http://purl.org/np/RAFH8AVn-wnTcSGxvPZ1Uiy_AtOhINlynnAxxiCdcTVWU#> .

@prefix np: <http://www.nanopub.org/nschema#> .

@prefix dct: <http://purl.org/dc/terms/> .

@prefix nt: <https://w3id.org/np/o/ntemplate/> .

@prefix npx: <http://purl.org/nanopub/x/> .

@prefix xsd: <http://www.w3.org/2001/XMLSchema#> .

@prefix rdfs: <http://www.w3.org/2000/01/rdf-schema#> .

@prefix orcid: <https://orcid.org/> .

@prefix prov: <http://www.w3.org/ns/prov#> .

@prefix skos: <http://www.w3.org/2004/02/skos/core#> .

sub:Head {

this: np:hasAssertion sub:assertion ;

np:hasProvenance sub:provenance ;

np:hasPublicationInfo sub:pubinfo ;

a np:Nanopublication .

}

sub:assertion {

sub:integrin-clustering a <http://www.w3.org/2002/07/owl#Class> ;

rdfs:label "integrin clustering" ;

rdfs:subClassOf <http://www.wikidata.org/entity/Q14633861> ;

skos:definition "clustering of integrin proteins at a focal adhesion" ;

skos:relatedMatch <http://www.wikidata.org/entity/Q904514> .

}

sub:provenance {

sub:assertion prov:wasAttributedTo orcid:0000-0002-0830-7029 .

}

sub:pubinfo {

sub:sig npx:hasAlgorithm "RSA" ;

npx:hasPublicKey "MIGfMA0GCSqGSIb3DQEBAQUAA4GNADCBiQKBgQCluZ2msgo7OqPkyRoZMluzpaLX8KRHyDs3J/cZwm6+Vq/CKVIGsGaT7/XH435cE9J8dwCgQ/Jssjlj6oqX+nSI9xXBtMH9ZeyGHFBzK1cUBy/rRSez4EnwbS1sKMBSc4MBCXQ9R3jZ3GpsN0GmIrS1ukKkUBQrREBYpb4w5MbGywIDAQAB" ;

npx:hasSignature "Bw052zD4Sd8+EkXb26m8oz03UEYZVfzKsvkEbf3j/zJeQJ/2PsYF94kN6QBs0mKoSeHJJQxOMOer6WCcTbYmltvLgG2zQYEFC3wkhRMTx5eEVjEMAXx4S57/Ur2a5yh+HzyqELJ1jG+2JIUQdHKO8F0F0VUqB2tyEf/ZhjMFz10=" ;

npx:hasSignatureTarget this: .

this: dct:created "2021-11-12T09:17:34.478-08:00"^^xsd:dateTime ;

dct:creator orcid:0000-0002-0830-7029 ;

npx:introduces sub:integrin-clustering ;

npx:supersedes <http://purl.org/np/RA9UaQM3wVa9xRAmL9CWEXyhDbcgvHlPoGTw4pnKaHYL8> ;

<https://w3id.org/linkflows/reviews/isUpdateOf> <http://purl.org/np/RA9UaQM3wVa9xRAmL9CWEXyhDbcgvHlPoGTw4pnKaHYL8> ;

nt:wasCreatedFromProvenanceTemplate <http://purl.org/np/RANwQa4ICWS5SOjw7gp99nBpXBasapwtZF1fIM3H2gYTM> ;

nt:wasCreatedFromPubinfoTemplate <http://purl.org/np/RAA2MfqdBCzmz9yVWjKLXNbyfBNcwsMmOqcNUxkk1maIM> , <http://purl.org/np/RAOGu9Lh0BD4tbIRB9RG6RGRA_ObDh75NTbIqaWgxxs8M> , <http://purl.org/np/RAjpBMlw3owYhJUBo3DtsuDlXsNAJ8cnGeWAutDVjuAuI> ;

nt:wasCreatedFromTemplate <http://purl.org/np/RAdpgRpigXtt8iPV9uOPf3wIT3qzOI8Sg2Q72CNV8g-Yo> .

}

**References**

[1] Paszek, M., DuFort, C., Rossier, O. et al. The cancer glycocalyx mechanically primes integrin-mediated growth and survival. Nature 511, 319–325 (2014). doi: 10.1038/nature13535.

[2] Bucur, C.I., Kuhn, T., Ceolin, D., Ossenbruggen, J. van. Expressing high-level scientific claims with formal semantics. In: Proceedings of the 11th Knowledge Capture Conference 2021. doi: 10.1145/3460210.3493561.
