# Supplementary material for: Nanopublication-based semantic publishing and reviewing: a field study with formalization papers
Source: PeerJ Comput Sci. 2023 Feb 21;9:e1159. doi: 10.7717/peerj-cs.1159 (PMC10280262; doi:10.7717/peerj-cs.1159)
Supplement: Supplemental Information 2 [file peerj-cs-09-1159-s002.zip › formalization_papers_supplemental-main/accepted_submissions/s14_Valentin_Groues&Carlos_Vega&Venkata_Satagopam.docx]

**Title:** A formalization of one of the main claims of “Mutations in STX1B, encoding a presynaptic protein, cause fever-associated epilepsy syndromes” by Schubert et al. 2014

**Authors:** Valentin Grouès^[1]^, ORCID: 0000-0001-6501-0806 and

Carlos Vega Moreno^[2]^, ORCID: 0000-0002-7979-9921 and

Venkata Pardhasaradhi Satagopam^[3]^, ORCID:0000-0002-6532-5880

**Affiliations:** ^[1]^ Luxembourg Centre for Systems Biomedicine, Université du Luxembourg, Luxemburg. E-mail: [valentin.groues@uni.lu](mailto:valentin.groues@uni.lu) ; ^[2]^ Luxembourg Centre for Systems Biomedicine, Université du Luxembourg, Luxemburg. E-mail: [carlos.vega@uni.lu](mailto:carlos.vega@uni.lu) ; ^[3]^ Luxembourg Centre for Systems Biomedicine, Université du Luxembourg, Luxemburg. E-mail: [venkata.satagopam@uni.lu](mailto:venkata.satagopam@uni.lu)

**Keywords:** “human”, “STX1B mutation”, “epilepsy”

**Article Type:** Formalization Paper

**As RDF/nanopublication:**

<http://purl.org/np/RAeRSya2qIYymsBxiqOZP_oaQpHXUVXiydKvPCFM-7DDQ>

**Editor:** Cristina-Iulia Bucur, ORCID: 0000-0002-7114-6459

**Review comments from:**

- Tobias Kuhn, ORCID: 0000-0002-1267-0234
- Victor de Boer, ORCID: 0000-0001-9079-039X
- Cristina-Iulia Bucur, ORCID: 0000-0002-7114-6459

**Received:** 2021-06-24

**Accepted:** 2021-11-17

**Abstract:**

Schubert et al. claimed in previous work that mutations in STX1B are associated with epilepsy. We present here a formalization of that claim, stating that all things of class “STX1B mutation” that are in the context of a thing of class “human” frequently have a relation of type “co-occurs with” to a thing of class “epilepsy” in the same context.

1. **Introduction**

Schubert et al. [1] state that “Our results thus implicate STX1B and the presynaptic release machinery in fever-associated epilepsy syndromes.”. We present here a formalization of the main scientific claim from this quote by using a semantic template called the super-pattern [2].

1. **Formalization**

Our formalization looks as follows:

| CONTEXT-CLASS (“in the context of all ..."): | [human](https://www.wikidata.org/wiki/Q5) |
| --- | --- |
| SUBJECT-CLASS (“things of type ..."): | [STX1B mutation](http://purl.org/np/RAPVWYH0x-xyDa9PfBcGUFly3m1FNEO43KG9s0uH-y6yo#STX1B-mutation) |
| QUALIFIER: | [frequently](https://w3id.org/linkflows/superpattern/terms/frequentlyQualifier) |
| RELATION-TYPE (“have a relation of type...”): | [co-occurs with](https://w3id.org/linkflows/superpattern/terms/coOccursWith) |
| OBJECT-CLASS (“to things of type...”): | [epilepsy](https://www.wikidata.org/wiki/Q41571) |

In the context class we use the class “human” (Q5) from Wikidata. In the subject class, we use a new minted class “STX1B mutation” that is a subclass of “mutation” (Q42918) from Wikidata and is related to the class “STX1B” (Q18048867) from Wikidata. In the object class we use the class “epilepsy” (Q41571) from Wikidata.

1. **RDF Code**

This is our formalization as a nanopublication in TriG format:

@prefix this: <http://purl.org/np/RAeRSya2qIYymsBxiqOZP_oaQpHXUVXiydKvPCFM-7DDQ> .

@prefix sub: <http://purl.org/np/RAeRSya2qIYymsBxiqOZP_oaQpHXUVXiydKvPCFM-7DDQ#> .

@prefix np: <http://www.nanopub.org/nschema#> .

@prefix dct: <http://purl.org/dc/terms/> .

@prefix nt: <https://w3id.org/np/o/ntemplate/> .

@prefix npx: <http://purl.org/nanopub/x/> .

@prefix xsd: <http://www.w3.org/2001/XMLSchema#> .

@prefix rdfs: <http://www.w3.org/2000/01/rdf-schema#> .

@prefix orcid: <https://orcid.org/> .

@prefix prov: <http://www.w3.org/ns/prov#> .

@prefix sp: <https://w3id.org/linkflows/superpattern/terms/> .

sub:Head {

this: np:hasAssertion sub:assertion ;

np:hasProvenance sub:provenance ;

np:hasPublicationInfo sub:pubinfo ;

a np:Nanopublication .

}

sub:assertion {

sub:spi a sp:SuperPatternInstance ;

rdfs:label "Mutations in STX1B are associated with epilepsy" ;

sp:hasContextClass <http://www.wikidata.org/entity/Q5> ;

sp:hasSubjectClass <http://purl.org/np/RAPVWYH0x-xyDa9PfBcGUFly3m1FNEO43KG9s0uH-y6yo#STX1B-mutation> ;

sp:hasQualifier sp:frequentlyQualifier ;

sp:hasRelation sp:cooccursWith ;

sp:hasObjectClass <http://www.wikidata.org/entity/Q41571> .

}

sub:provenance {

sub:activity a sp:FormalizationActivity ;

prov:used <http://doi.org/10.1038/ng.3130> , sub:quote ;

prov:wasAssociatedWith orcid:0000-0001-6501-0806 , orcid:0000-0002-6532-5880 , orcid:0000-0002-7979-9921 .

sub:assertion prov:wasGeneratedBy sub:activity .

sub:quote prov:value "Our results thus implicate STX1B and the presynaptic release machinery in fever-associated epilepsy syndromes" ;

prov:wasQuotedFrom <http://doi.org/10.1038/ng.3130> .

}

sub:pubinfo {

sub:sig npx:hasAlgorithm "RSA" ;

npx:hasPublicKey "MIGfMA0GCSqGSIb3DQEBAQUAA4GNADCBiQKBgQCY36SLWPLee0SZGM108+7dyjGzKFYg9t09XuL3js13jO3CDzqAZygcrwbJsbLQMRHYvWf0Mkly1ePLgdb43NqEbXiDHC4o49nHjhi2bSWeRDJ4jFicvhpL1Sjv5N0oFU3wTA8KGN+rYvQ10xhZXcjuuiCMb6yInj6wJ2S+wI14fwIDAQAB" ;

npx:hasSignature "QF+C9lXmczrn9cJWuimwLG45Mpntk2CcRIWbeWmKvfE9gmQ6MPKa/x6AfNgVQRnPWppJdDoWepK6m/+m8tWY1WQsXn0KZ8sER+graEHQYuE70Mz9JzuBTyYu0vpWj5jteoCve5fyvFkhkYVjoRK9tV40GDW6zh+bHqH5tBwrc/0=" ;

npx:hasSignatureTarget this: .

this: dct:created "2021-10-29T10:35:33.912+02:00"^^xsd:dateTime ;

dct:creator orcid:0000-0001-6501-0806 ;

npx:introduces sub:spi ;

<https://w3id.org/linkflows/reviews/isUpdateOf> <http://purl.org/np/RAGo62Hb_Bx1klF4pn1q1Ty40860e3A7Sz4hr2vojZ2wA> ;

nt:wasCreatedFromProvenanceTemplate <http://purl.org/np/RAB_oy10D3XUP-zYlqGz7Uj58AsUXhEKeGqmRFg5LSgDM> ;

nt:wasCreatedFromPubinfoTemplate <http://purl.org/np/RAA2MfqdBCzmz9yVWjKLXNbyfBNcwsMmOqcNUxkk1maIM> , <http://purl.org/np/RAOGu9Lh0BD4tbIRB9RG6RGRA_ObDh75NTbIqaWgxxs8M> ;

nt:wasCreatedFromTemplate <http://purl.org/np/RAv68imZrEjfcp2rnEg1hzoBqEVc0cQMtp9_1Za0BxNM4> .

}

The following nanopublications introduce the newly minted classes in TriG format.

This is the class definition of “STX1B mutation”:

@prefix this: <http://purl.org/np/RAPVWYH0x-xyDa9PfBcGUFly3m1FNEO43KG9s0uH-y6yo> .

@prefix sub: <http://purl.org/np/RAPVWYH0x-xyDa9PfBcGUFly3m1FNEO43KG9s0uH-y6yo#> .

@prefix np: <http://www.nanopub.org/nschema#> .

@prefix dct: <http://purl.org/dc/terms/> .

@prefix nt: <https://w3id.org/np/o/ntemplate/> .

@prefix npx: <http://purl.org/nanopub/x/> .

@prefix xsd: <http://www.w3.org/2001/XMLSchema#> .

@prefix rdfs: <http://www.w3.org/2000/01/rdf-schema#> .

@prefix orcid: <https://orcid.org/> .

@prefix prov: <http://www.w3.org/ns/prov#> .

@prefix skos: <http://www.w3.org/2004/02/skos/core#> .

sub:Head {

this: np:hasAssertion sub:assertion ;

np:hasProvenance sub:provenance ;

np:hasPublicationInfo sub:pubinfo ;

a np:Nanopublication .

}

sub:assertion {

sub:STX1B-mutation a <http://www.w3.org/2002/07/owl#Class> ;

rdfs:label "STX1B mutation" ;

rdfs:subClassOf <http://www.wikidata.org/entity/Q42918> ;

skos:definition "mutation in STX1B" ;

skos:relatedMatch <http://www.wikidata.org/entity/Q18048867> .

}

sub:provenance {

sub:assertion prov:wasAttributedTo orcid:0000-0001-6501-0806 .

}

sub:pubinfo {

sub:sig npx:hasAlgorithm "RSA" ;

npx:hasPublicKey "MIGfMA0GCSqGSIb3DQEBAQUAA4GNADCBiQKBgQCY36SLWPLee0SZGM108+7dyjGzKFYg9t09XuL3js13jO3CDzqAZygcrwbJsbLQMRHYvWf0Mkly1ePLgdb43NqEbXiDHC4o49nHjhi2bSWeRDJ4jFicvhpL1Sjv5N0oFU3wTA8KGN+rYvQ10xhZXcjuuiCMb6yInj6wJ2S+wI14fwIDAQAB" ;

npx:hasSignature "EVu/+D116w+WZYRCxa5Q4AgDxW7pGuClv6tHouXYSGTCgGu9nZo2pjEqDBmiUyWY8iYyr4FMxGJz2E0bwMU9Ui5svH0EBZPiJQHT6tvfrSRYOP7txHN/LMaxn+IWRp4Ed1kN9/q5J+BUnnD7x6UghL2s/VlnpSWjVq/EJocnDbU=" ;

npx:hasSignatureTarget this: .

this: dct:created "2021-06-21T14:08:44.937+02:00"^^xsd:dateTime ;

dct:creator orcid:0000-0001-6501-0806 ;

npx:introduces sub:STX1B-mutation ;

nt:wasCreatedFromProvenanceTemplate <http://purl.org/np/RANwQa4ICWS5SOjw7gp99nBpXBasapwtZF1fIM3H2gYTM> ;

nt:wasCreatedFromPubinfoTemplate <http://purl.org/np/RAA2MfqdBCzmz9yVWjKLXNbyfBNcwsMmOqcNUxkk1maIM> ;

nt:wasCreatedFromTemplate <http://purl.org/np/RAdpgRpigXtt8iPV9uOPf3wIT3qzOI8Sg2Q72CNV8g-Yo> .

}

**References**

[1] Schubert, J., Siekierska, A., Langlois, M. et al. Mutations in STX1B, encoding a presynaptic protein, cause fever-associated epilepsy syndromes. Nat Genet 46, 1327–1332 (2014). doi: 10.1038/ng.3130.

[2] Bucur, C.I., Kuhn, T., Ceolin, D., Ossenbruggen, J. van. Expressing high-level scientific claims with formal semantics. In: Proceedings of the 11th Knowledge Capture Conference 2021. doi: 10.1145/3460210.3493561.
