# Supplementary material for: Nanopublication-based semantic publishing and reviewing: a field study with formalization papers
Source: PeerJ Comput Sci. 2023 Feb 21;9:e1159. doi: 10.7717/peerj-cs.1159 (PMC10280262; doi:10.7717/peerj-cs.1159)
Supplement: Supplemental Information 2 [file peerj-cs-09-1159-s002.zip › formalization_papers_supplemental-main/accepted_submissions/s14_Valentin_Groues&Carlos_Vega&Venkata_Satagopam.pdf]

Title: A formalization of one of the main claims of “Mutations in STX1B, encoding a presynaptic protein, cause fever-associated epilepsy syndromes” by Schubert et al. 2014

Authors: Valentin Grouès[1], ORCID: 0000-0001-6501-0806 and
Carlos Vega Moreno[2], ORCID: 0000-0002-7979-9921 and
Venkata Pardhasaradhi Satagopam[3], ORCID:0000-0002-6532-5880

Affiliations: [1] Luxembourg Centre for Systems Biomedicine, Université du Luxembourg, Luxemburg. E-mail: valentin.groues@uni.lu ; [2] Luxembourg Centre for Systems Biomedicine, Université du Luxembourg, Luxemburg. E-mail: carlos.vega@uni.lu ; [3] Luxembourg Centre for Systems Biomedicine, Université du Luxembourg, Luxemburg. E-mail: venkata.satagopam@uni.lu

Keywords: “human”, “STX1B mutation”, “epilepsy”

Article Type: Formalization Paper

As RDF/nanopublication:
http://purl.org/np/RAeRSya2qIYymsBxiqOZP_oaQpHXUVXiydKvPCFM-7DDQ

Editor: Cristina-Iulia Bucur, ORCID: 0000-0002-7114-6459

Review comments from:
- Tobias Kuhn, ORCID: 0000-0002-1267-0234
- Victor de Boer, ORCID: 0000-0001-9079-039X
- Cristina-Iulia Bucur, ORCID: 0000-0002-7114-6459

Received: 2021-06-24

Accepted: 2021-11-17

Abstract:
Schubert et al. claimed in previous work that mutations in STX1B are associated with epilepsy. We present here a formalization of that claim, stating that all things of class “STX1B mutation” that are in the context of a thing of class “human” frequently have a relation of type “co-occurs with” to a thing of class “epilepsy” in the same context.

1. Introduction

Schubert et al. [1] state that “Our results thus implicate STX1B and the presynaptic release machinery in fever-associated epilepsy syndromes.”. We present here a formalization of the main scientific claim from this quote by using a semantic template called the super-pattern [2].

2. Formalization

Our formalization looks as follows:

CONTEXT-CLASS (“in the context of all ..."):	human
SUBJECT-CLASS (“things of type ..."):	STX1B mutation
QUALIFIER:	frequently
RELATION-TYPE (“have a relation of type...”):	co-occurs with
OBJECT-CLASS (“to things of type...”):	epilepsy

In the context class we use the class “human” (Q5) from Wikidata. In the subject class, we use a new minted class “STX1B mutation” that is a subclass of “mutation” (Q42918) from Wikidata and is related to the class “STX1B” (Q18048867) from Wikidata. In the object class we use the class “epilepsy” (Q41571) from Wikidata.

3. RDF Code

This is our formalization as a nanopublication in TriG format:

```
@prefix this: <http://purl.org/np/RAeRSya2qIYymsBxiqOZP_oaQpHXUVXiydKvPCFM-7DDQ> .
@prefix sub: <http://purl.org/np/RAeRSya2qIYymsBxiqOZP_oaQpHXUVXiydKvPCFM-7DDQ#> .
@prefix np: <http://www.nanopub.org/nschema#> .
@prefix dct: <http://purl.org/dc/terms/> .
@prefix nt: <https://w3id.org/np/o/ntemplate/> .
@prefix npx: <http://purl.org/nanopub/x/> .
@prefix xsd: <http://www.w3.org/2001/XMLSchema#> .
@prefix rdfs: <http://www.w3.org/2000/01/rdf-schema#> .
@prefix orcid: <https://orcid.org/> .
@prefix prov: <http://www.w3.org/ns/prov#> .
@prefix sp: <https://w3id.org/linkflows/superpattern/terms/> .

sub:Head {
  this: np:hasAssertion sub:assertion ;
    np:hasProvenance sub:provenance ;
    np:hasPublicationInfo sub:pubinfo ;
    a np:Nanopublication .
}
sub:assertion {
  sub:spi a sp:SuperPatternInstance ;
    rdfs:label "Mutations in STX1B are associated with epilepsy" ;
    sp:hasContextClass <http://www.wikidata.org/entity/Q5> ;
    sp:hasSubjectClass <http://purl.org/np/RAPVWYH0x-xyDa9PfBcGUFly3m1FNEO43KG9s0uH-y6yo#STX1B-mutation> ;
    sp:hasQualifier sp:frequentlyQualifier ;
    sp:hasRelation sp:cooccursWith ;
    sp:hasObjectClass <http://www.wikidata.org/entity/Q41571> .
}
sub:provenance {
  sub:activity a sp:FormalizationActivity ;
    prov:used <http://doi.org/10.1038/ng.3130> , sub:quote ;
    prov:wasAssociatedWith orcid:0000-0001-6501-0806 , orcid:0000-0002-6532-5880 , orcid:0000-0002-7979-9921 .
  sub:assertion prov:wasGeneratedBy sub:activity .
  sub:quote prov:value "Our results thus implicate STX1B and the presynaptic release machinery in fever-associated epilepsy
syndromes" ;
    prov:wasQuotedFrom <http://doi.org/10.1038/ng.3130> .
}
sub:pubinfo {
  sub:sig npx:hasAlgorithm "RSA" ;
    npx:hasPublicKey
"MIGfMA0GCSqGSIb3DQEBAQUAA4GNADCBiQKBgQCY36SLWPLee0SZGM108+7dyjGzKFYg9t09XuL3js13jO3CDzqAZygcrwbJsbLQMRHYvWf0Mkly1ePLgdb43NqEbXiD
HC4o49nHjhi2bSWeRDJ4jFicvhpL1Sjv5N0oFU3wTA8KGN+rYvQ10xhZXcjuuiCMb6yInj6wJ2S+wI14fwIDAQAB" ;
```

```
    npx:hasSignature
"QF+C9lXmczrn9cJWuimwLG45Mpntk2CcRIWbeWmKvfE9gmQ6MPKa/x6AfNgVQRnPWppJdDoWepK6m/+m8tWY1WQsXn0KZ8sER+graEHQYuE70Mz9JzuBTyYu0vpWj5jt
eoCve5fyvFkhkYVjoRK9tV40GDW6zh+bHqH5tBwrc/0=" ;
    npx:hasSignatureTarget this: .
  this: dct:created "2021-10-29T10:35:33.912+02:00"^^xsd:dateTime ;
    dct:creator orcid:0000-0001-6501-0806 ;
    npx:introduces sub:spi ;
    <https://w3id.org/linkflows/reviews/isUpdateOf> <http://purl.org/np/RAGo62Hb_Bx1klF4pn1q1Ty40860e3A7Sz4hr2vojZ2wA> ;
    nt:wasCreatedFromProvenanceTemplate <http://purl.org/np/RAB_oy10D3XUP-zYlqGz7Uj58AsUXhEKeGqmRFg5LSgDM> ;
    nt:wasCreatedFromPubinfoTemplate <http://purl.org/np/RAA2MfqdBCzmz9yVWjKLXNbyfBNcwsMmOqcNUxkk1maIM> ,
<http://purl.org/np/RAOGu9Lh0BD4tbIRB9RG6RGRA_ObDh75NTbIqaWgxxs8M> ;
    nt:wasCreatedFromTemplate <http://purl.org/np/RAv68imZrEjfcp2rnEg1hzoBqEVc0cQMtp9_1Za0BxNM4> .
}
```

The following nanopublications introduce the newly minted classes in TriG format.

This is the class definition of “STX1B mutation”:

```
@prefix this: <http://purl.org/np/RAPVWYH0x-xyDa9PfBcGUFly3m1FNEO43KG9s0uH-y6yo> .
@prefix sub: <http://purl.org/np/RAPVWYH0x-xyDa9PfBcGUFly3m1FNEO43KG9s0uH-y6yo#> .
@prefix np: <http://www.nanopub.org/nschema#> .
@prefix dct: <http://purl.org/dc/terms/> .
@prefix nt: <https://w3id.org/np/o/ntemplate/> .
@prefix npx: <http://purl.org/nanopub/x/> .
@prefix xsd: <http://www.w3.org/2001/XMLSchema#> .
@prefix rdfs: <http://www.w3.org/2000/01/rdf-schema#> .
@prefix orcid: <https://orcid.org/> .
@prefix prov: <http://www.w3.org/ns/prov#> .
@prefix skos: <http://www.w3.org/2004/02/skos/core#> .

sub:Head {
  this: np:hasAssertion sub:assertion ;
    np:hasProvenance sub:provenance ;
    np:hasPublicationInfo sub:pubinfo ;
    a np:Nanopublication .
}
sub:assertion {
  sub:STX1B-mutation a <http://www.w3.org/2002/07/owl#Class> ;
    rdfs:label "STX1B mutation" ;
    rdfs:subClassOf <http://www.wikidata.org/entity/Q42918> ;
    skos:definition "mutation in STX1B" ;
    skos:relatedMatch <http://www.wikidata.org/entity/Q18048867> .
}
sub:provenance {
  sub:assertion prov:wasAttributedTo orcid:0000-0001-6501-0806 .
}
sub:pubinfo {
  sub:sig npx:hasAlgorithm "RSA" ;
    npx:hasPublicKey
"MIGfMA0GCSqGSIb3DQEBAQUAA4GNADCBiQKBgQCY36SLWPLee0SZGM108+7dyjGzKFYg9t09XuL3js13jO3CDzqAZygcrwbJsbLQMRHYvWf0Mkly1ePLgdb43NqEbXiD
HC4o49nHjhi2bSWeRDJ4jFicvhpL1Sjv5N0oFU3wTA8KGN+rYvQ10xhZXcjuuiCMb6yInj6wJ2S+wI14fwIDAQAB" ;
    npx:hasSignature
"EVu/+D116w+WZYRCxa5Q4AgDxW7pGuClv6tHouXYSGTCgGu9nZo2pjEqDBmiUyWY8iYyr4FMxGJz2E0bwMU9Ui5svH0EBZPiJQHT6tvfrSRYOP7txHN/LMaxn+IWRp4E
d1kN9/q5J+BUnnD7x6UghL2s/VlnpSWjVq/EJocnDbU=" ;
    npx:hasSignatureTarget this: .
  this: dct:created "2021-06-21T14:08:44.937+02:00"^^xsd:dateTime ;
    dct:creator orcid:0000-0001-6501-0806 ;
    npx:introduces sub:STX1B-mutation ;
    nt:wasCreatedFromProvenanceTemplate <http://purl.org/np/RANwQa4ICWS5SOjw7gp99nBpXBasapwtZF1fIM3H2gYTM> ;
    nt:wasCreatedFromPubinfoTemplate <http://purl.org/np/RAA2MfqdBCzmz9yVWjKLXNbyfBNcwsMmOqcNUxkk1maIM> ;
    nt:wasCreatedFromTemplate <http://purl.org/np/RAdpgRpigXtt8iPV9uOPf3wIT3qzOI8Sg2Q72CNV8g-Yo> .
}
```

References

[1] Schubert, J., Siekierska, A., Langlois, M. et al. Mutations in STX1B, encoding a presynaptic protein, cause fever-associated epilepsy syndromes. Nat Genet 46, 1327–1332 (2014). doi: 10.1038/ng.3130.

[2] Bucur, C.I., Kuhn, T., Ceolin, D., Ossenbruggen, J. van. Expressing high-level scientific claims with formal semantics. In: Proceedings of the 11th Knowledge Capture Conference 2021. doi: 10.1145/3460210.3493561.
