# Supplementary material for: Nanopublication-based semantic publishing and reviewing: a field study with formalization papers
Source: PeerJ Comput Sci. 2023 Feb 21;9:e1159. doi: 10.7717/peerj-cs.1159 (PMC10280262; doi:10.7717/peerj-cs.1159)
Supplement: Supplemental Information 2 [file peerj-cs-09-1159-s002.zip › formalization_papers_supplemental-main/accepted_submissions/s15_Victor_de_Boer.docx]

**Title:** A formalization of one of the main claims of “A Linked Data Model for Data Scopes” by De Boer et al. 2021

**Authors:** Victor de Boer, ORCID: 0000-0001-9079-039X

**Affiliations:** Vrije Universiteit Amsterdam, The Netherlands. E-mail: [v.de.boer@vu.nl](mailto:v.de.boer@vu.nl)

**Keywords:** “digital humanities research”, “usage of Linked Data Scopes”, “transparency”

**Article Type:** Formalization Paper

**As RDF/nanopublication:** <http://purl.org/np/RAgoIxfXPqNDY8vnK2EmBQDAFwuFIDJtfaMplTvPMq3pg>

**Editor:** Cristina-Iulia Bucur, ORCID: 0000-0002-7114-6459

**Review comments from:**

- Tobias Kuhn, ORCID: 0000-0002-1267-0234
- Ricardo Usbeck, ORCID: 0000-0002-0191-7211
- Margherita Martorana, ORCID: 0000-0001-8004-0464
- Davide Ceolin, ORCID: 0000-0002-3357-9130
- Mariya Dimitrova, ORCID: 0000-0002-8083-6048
- Cristina-Iulia Bucur, ORCID: 0000-0002-7114-6459

**Received:** 2021-06-24

**Accepted:** 2021-11-19

**Abstract:**

De Boer et al. claimed in previous work that in the context of Digital Humanities research, usage of the Linked Data Scopes ontology contributes to transparency of the research. We present here a formalization of that claim, stating that all things of class “usage of Linked Data Scopes” that are in the context of a thing of class “digital humanities research” can generally have a relation of type “contributes to” to a thing of class “transparency” in the same context.

1. **Introduction**

De Boer et al. [1] state that “With the rise of data driven methods in the humanities, it becomes necessary to develop reusable and consistent methodological patterns for dealing with the various data manipulation steps. This increases transparency, replicability of the research. Data scopes present a qualitative framework for such methodological steps. In this work we present a Linked Data model to represent and share Data Scopes”. We present here a formalization of the main scientific claim from this quote by using a semantic template called the super-pattern [2].

1. **Formalization**

Our formalization looks as follows:

| CONTEXT-CLASS (“in the context of all ..."): | [digital humanities research](http://purl.org/np/RAkCjYmMU3obIrC4IpwUw84JW1ymd312yz0N0g-R9yes0#digital-humanities-research) |
| --- | --- |
| SUBJECT-CLASS (“things of type ..."): | [usage of Linked Data Scopes](http://purl.org/np/RAcPa1aO8kAt8QYwjQoJq-PIzYvo0jCzYrAiOX_XOyk1w#usage-of-linked-data-scopes) |
| QUALIFIER: | [can generally](https://w3id.org/linkflows/superpattern/terms/canGenerallyQualifier) |
| RELATION-TYPE (“have a relation of type...”): | [contributes to](https://w3id.org/linkflows/superpattern/terms/contributesTo) |
| OBJECT-CLASS (“to things of type...”): | [transparency](https://www.wikidata.org/wiki/Q535347) |

In the context class we use a new minted class “digital humanities research” that is a subclass of “research” (Q42240) from Wikidata and is related to the class “digital humanities” (Q1026962) from Wikidata. In the subject class, we use a new minted class “usage of Linked Data Scopes” that is defined using the restriction “some values from” from OWL on ”the Data Scopes Ontology” with the property “uses” (P2283) from Wikidata. In the object class we use the class “transparency” (Q535347) from Wikidata.

1. **RDF Code**

This is our formalization as a nanopublication in TriG format:

@prefix this: <http://purl.org/np/RAgoIxfXPqNDY8vnK2EmBQDAFwuFIDJtfaMplTvPMq3pg> .

@prefix sub: <http://purl.org/np/RAgoIxfXPqNDY8vnK2EmBQDAFwuFIDJtfaMplTvPMq3pg#> .

@prefix np: <http://www.nanopub.org/nschema#> .

@prefix dct: <http://purl.org/dc/terms/> .

@prefix nt: <https://w3id.org/np/o/ntemplate/> .

@prefix npx: <http://purl.org/nanopub/x/> .

@prefix xsd: <http://www.w3.org/2001/XMLSchema#> .

@prefix rdfs: <http://www.w3.org/2000/01/rdf-schema#> .

@prefix orcid: <https://orcid.org/> .

@prefix prov: <http://www.w3.org/ns/prov#> .

@prefix sp: <https://w3id.org/linkflows/superpattern/terms/> .

sub:Head {

this: np:hasAssertion sub:assertion ;

np:hasProvenance sub:provenance ;

np:hasPublicationInfo sub:pubinfo ;

a np:Nanopublication .

}

sub:assertion {

sub:spi a sp:SuperPatternInstance ;

rdfs:label "In the context of Digital Humanities research, usage of the Linked Data Scopes ontology contributes to transparency of the research." ;

sp:hasContextClass <http://purl.org/np/RAkCjYmMU3obIrC4IpwUw84JW1ymd312yz0N0g-R9yes0#digital-humanities-research> ;

sp:hasSubjectClass <http://purl.org/np/RAcPa1aO8kAt8QYwjQoJq-PIzYvo0jCzYrAiOX_XOyk1w#usage-of-linked-data-scopes> ;

sp:hasQualifier sp:canGenerallyQualifier ;

sp:hasRelation sp:contributesTo ;

sp:hasObjectClass <https://www.wikidata.org/wiki/Q535347> .

}

sub:provenance {

sub:activity a sp:FormalizationActivity ;

prov:used sub:quote , <https://doi.org/10.1007%2F978-3-030-71903-6_32> ;

prov:wasAssociatedWith orcid:0000-0001-9079-039X .

sub:assertion prov:wasGeneratedBy sub:activity .

sub:quote prov:value "With the rise of data driven methods in the humanities, it becomes necessary to develop reusable and consistent methodological patterns for dealing with the various data manipulation steps. This increases transparency, replicability of the research. Data scopes present a qualitative framework for such methodological steps. In this work we present a Linked Data model to represent and share Data Scopes" ;

prov:wasQuotedFrom <https://doi.org/10.1007%2F978-3-030-71903-6_32> .

}

sub:pubinfo {

sub:sig npx:hasAlgorithm "RSA" ;

npx:hasPublicKey "MIGfMA0GCSqGSIb3DQEBAQUAA4GNADCBiQKBgQCUtUS5tVuMGtBUJ+dpxk0EKcI3xKBG4efTcKjAMHeQj6b+liabAld6kXxQKQIRVQomBKOcvoYJ9oTC4K4AMUPMfQsPSLt1noFxSahw86AXdAh7kWiVZu04Vs0NDuPPUOWwRQjXRf6gYFLQu3gA5o0PkWd7FZ6lrum6lNmi/LFQdwIDAQAB" ;

npx:hasSignature "Zn7f/GSB5lE7q1x9oYoWH2jMAnMIGJhh5bdy6spvltV7O2OWok82jK7IcZoHdtFrzFcMKyrb18VhXJD6KY1NFP2zui5n0gyBNzv/aruPWUdTx8WaijNQv75R96ZmUHIrWknF8lpF53HjGK1TfIVUevYvOlNFv88DiQdMo9naSPM=" ;

npx:hasSignatureTarget this: .

this: dct:created "2021-11-19T09:25:07.665+01:00"^^xsd:dateTime ;

dct:creator orcid:0000-0001-9079-039X ;

npx:introduces sub:spi ;

npx:supersedes <http://purl.org/np/RA0HU7k7XUj81ioBftZFk7ywHx5uBBlVgXduglUYgyW5w> ;

<https://w3id.org/linkflows/reviews/isUpdateOf> <http://purl.org/np/RATc9O9tCdQPlrsJeGNsTNO0PbENIzdKa6XAOex7dXfrY> ;

nt:wasCreatedFromProvenanceTemplate <http://purl.org/np/RAB_oy10D3XUP-zYlqGz7Uj58AsUXhEKeGqmRFg5LSgDM> ;

nt:wasCreatedFromPubinfoTemplate <http://purl.org/np/RA2vCBXZf-icEcVRGhulJXugTGxpsV5yVr9yqCI1bQh4A> , <http://purl.org/np/RAA2MfqdBCzmz9yVWjKLXNbyfBNcwsMmOqcNUxkk1maIM> , <http://purl.org/np/RAjpBMlw3owYhJUBo3DtsuDlXsNAJ8cnGeWAutDVjuAuI> ;

nt:wasCreatedFromTemplate <http://purl.org/np/RAv68imZrEjfcp2rnEg1hzoBqEVc0cQMtp9_1Za0BxNM4> .

}

The following nanopublications introduce the newly minted classes in TriG format.

This is the class definition of “digital humanities research”:

@prefix this: <http://purl.org/np/RAkCjYmMU3obIrC4IpwUw84JW1ymd312yz0N0g-R9yes0> .

@prefix sub: <http://purl.org/np/RAkCjYmMU3obIrC4IpwUw84JW1ymd312yz0N0g-R9yes0#> .

@prefix np: <http://www.nanopub.org/nschema#> .

@prefix dct: <http://purl.org/dc/terms/> .

@prefix nt: <https://w3id.org/np/o/ntemplate/> .

@prefix npx: <http://purl.org/nanopub/x/> .

@prefix xsd: <http://www.w3.org/2001/XMLSchema#> .

@prefix rdfs: <http://www.w3.org/2000/01/rdf-schema#> .

@prefix orcid: <https://orcid.org/> .

@prefix prov: <http://www.w3.org/ns/prov#> .

@prefix skos: <http://www.w3.org/2004/02/skos/core#> .

sub:Head {

this: np:hasAssertion sub:assertion ;

np:hasProvenance sub:provenance ;

np:hasPublicationInfo sub:pubinfo ;

a np:Nanopublication .

}

sub:assertion {

sub:digital-humanities-research a <http://www.w3.org/2002/07/owl#Class> ;

rdfs:label "Digital Humanities Research" ;

rdfs:subClassOf <http://www.wikidata.org/entity/Q42240> ;

skos:definition "systematic study undertaken to increase knowledge in the field of Digital Humanities" ;

skos:relatedMatch <http://www.wikidata.org/entity/Q1026962> .

}

sub:provenance {

sub:assertion prov:wasAttributedTo orcid:0000-0001-9079-039X .

}

sub:pubinfo {

sub:sig npx:hasAlgorithm "RSA" ;

npx:hasPublicKey "MIGfMA0GCSqGSIb3DQEBAQUAA4GNADCBiQKBgQCUtUS5tVuMGtBUJ+dpxk0EKcI3xKBG4efTcKjAMHeQj6b+liabAld6kXxQKQIRVQomBKOcvoYJ9oTC4K4AMUPMfQsPSLt1noFxSahw86AXdAh7kWiVZu04Vs0NDuPPUOWwRQjXRf6gYFLQu3gA5o0PkWd7FZ6lrum6lNmi/LFQdwIDAQAB" ;

npx:hasSignature "iXaNMYj4CT3YA+x8F4yuNplPhG8xjxeAAep0/7CYZS2LQlD2ovNJMvJCSxqLp6qhraPpSo9hnWeS/zLL1TwMfT4W0KtjQnET8mqI2kpqC9XYllcch2jr2sVPgiL7YgR9mir9CzEXHRchU8m6QH52twnsfQWtN3uLo+rOl2hMmJs=" ;

npx:hasSignatureTarget this: .

this: dct:created "2021-10-26T14:11:45.332+02:00"^^xsd:dateTime ;

dct:creator orcid:0000-0001-9079-039X ;

npx:introduces sub:digital-humanities-research ;

nt:wasCreatedFromProvenanceTemplate <http://purl.org/np/RANwQa4ICWS5SOjw7gp99nBpXBasapwtZF1fIM3H2gYTM> ;

nt:wasCreatedFromPubinfoTemplate <http://purl.org/np/RAA2MfqdBCzmz9yVWjKLXNbyfBNcwsMmOqcNUxkk1maIM> ;

nt:wasCreatedFromTemplate <http://purl.org/np/RAdpgRpigXtt8iPV9uOPf3wIT3qzOI8Sg2Q72CNV8g-Yo> .

}

This is the class definition of “usage of Linked Data Scopes”:

@prefix this: <http://purl.org/np/RAcPa1aO8kAt8QYwjQoJq-PIzYvo0jCzYrAiOX_XOyk1w> .

@prefix sub: <http://purl.org/np/RAcPa1aO8kAt8QYwjQoJq-PIzYvo0jCzYrAiOX_XOyk1w#> .

@prefix np: <http://www.nanopub.org/nschema#> .

@prefix dct: <http://purl.org/dc/terms/> .

@prefix nt: <https://w3id.org/np/o/ntemplate/> .

@prefix npx: <http://purl.org/nanopub/x/> .

@prefix xsd: <http://www.w3.org/2001/XMLSchema#> .

@prefix rdfs: <http://www.w3.org/2000/01/rdf-schema#> .

@prefix orcid: <https://orcid.org/> .

@prefix prov: <http://www.w3.org/ns/prov#> .

@prefix owl: <http://www.w3.org/2002/07/owl#> .

sub:Head {

this: np:hasAssertion sub:assertion ;

np:hasProvenance sub:provenance ;

np:hasPublicationInfo sub:pubinfo ;

a np:Nanopublication .

}

sub:assertion {

sub:usage-of-linked-data-scopes dct:description "Usage of the Linked Data Scopes ontology in a research project" ;

a owl:Class , owl:Restriction ;

rdfs:label "Usage of the Linked Data Scopes ontology" ;

owl:onProperty <http://wikidata.org/prop/direct/P2283> ;

owl:someValuesFrom <https://w3id.org/datascope/> .

}

sub:provenance {

sub:assertion prov:wasAttributedTo orcid:0000-0001-9079-039X .

}

sub:pubinfo {

sub:sig npx:hasAlgorithm "RSA" ;

npx:hasPublicKey "MIGfMA0GCSqGSIb3DQEBAQUAA4GNADCBiQKBgQCUtUS5tVuMGtBUJ+dpxk0EKcI3xKBG4efTcKjAMHeQj6b+liabAld6kXxQKQIRVQomBKOcvoYJ9oTC4K4AMUPMfQsPSLt1noFxSahw86AXdAh7kWiVZu04Vs0NDuPPUOWwRQjXRf6gYFLQu3gA5o0PkWd7FZ6lrum6lNmi/LFQdwIDAQAB" ;

npx:hasSignature "CrXMFJLR+Q5CY5wK6uM5TWBFMfb+S3RR77LhqkIUcjwml9ARVAixA6iAyRqUAo/Of3vpJdzPzjoGbieJGYLhbPfn3bVC70Zwzwv1S/+g8jtrNmshNR1ZBsQ/D5zkLHjEdXx52VP+Sqmg4YTQsm7oOTtKu9jZfJo33MS9sogr8B4=" ;

npx:hasSignatureTarget this: .

this: dct:created "2021-11-19T09:23:19.603+01:00"^^xsd:dateTime ;

dct:creator orcid:0000-0001-9079-039X ;

npx:introduces sub:usage-of-linked-data-scopes ;

npx:supersedes <http://purl.org/np/RAUxfk7XeLxn-2hiX3xWSlE5LXgkNW0MIFBGe2h3Bb3pM> ;

nt:wasCreatedFromProvenanceTemplate <http://purl.org/np/RANwQa4ICWS5SOjw7gp99nBpXBasapwtZF1fIM3H2gYTM> ;

nt:wasCreatedFromPubinfoTemplate <http://purl.org/np/RAA2MfqdBCzmz9yVWjKLXNbyfBNcwsMmOqcNUxkk1maIM> , <http://purl.org/np/RAjpBMlw3owYhJUBo3DtsuDlXsNAJ8cnGeWAutDVjuAuI> ;

nt:wasCreatedFromTemplate <http://purl.org/np/RAX7opQ5Tv_uIMSyQNujbJFJO4JLa-xuTUitQlw2aQ1eU> .

}

**References**

[1] de Boer V., Bonestroo I., Koolen M., Hoekstra R. (2021) A Linked Data Model for Data Scopes. In: Garoufallou E., Ovalle-Perandones MA. (eds) Metadata and Semantic Research. MTSR 2020. Communications in Computer and Information Science, vol 1355. Springer, Cham. doi: 10.1007/978-3-030-71903-6_32.

[2] Bucur, C.I., Kuhn, T., Ceolin, D., Ossenbruggen, J. van. Expressing high-level scientific claims with formal semantics. In: Proceedings of the 11th Knowledge Capture Conference 2021. doi: 10.1145/3460210.3493561.
