# Supplementary material for: Nanopublication-based semantic publishing and reviewing: a field study with formalization papers
Source: PeerJ Comput Sci. 2023 Feb 21;9:e1159. doi: 10.7717/peerj-cs.1159 (PMC10280262; doi:10.7717/peerj-cs.1159)
Supplement: Supplemental Information 2 [file peerj-cs-09-1159-s002.zip › formalization_papers_supplemental-main/accepted_submissions/s15_Victor_de_Boer.pdf]

Title: A formalization of one of the main claims of “A Linked Data Model for Data Scopes” by De Boer et al. 2021

Authors: Victor de Boer, ORCID: 0000-0001-9079-039X

Affiliations: Vrije Universiteit Amsterdam, The Netherlands. E-mail: v.de.boer@vu.nl

Keywords: “digital humanities research”, “usage of Linked Data Scopes”, “transparency”

Article Type: Formalization Paper

As RDF/nanopublication:
http://purl.org/np/RAgoIxfXPqNDY8vnK2EmBQDAFwuFIDJtfaMplTvPMq3pg

Editor: Cristina-Iulia Bucur, ORCID: 0000-0002-7114-6459

Review comments from:
- Tobias Kuhn, ORCID: 0000-0002-1267-0234
- Ricardo Usbeck, ORCID: 0000-0002-0191-7211
- Margherita Martorana, ORCID: 0000-0001-8004-0464
- Davide Ceolin, ORCID: 0000-0002-3357-9130
- Mariya Dimitrova, ORCID: 0000-0002-8083-6048
- Cristina-Iulia Bucur, ORCID: 0000-0002-7114-6459

Received: 2021-06-24

Accepted: 2021-11-19

Abstract:
De Boer et al. claimed in previous work that in the context of Digital Humanities research, usage of the Linked Data Scopes ontology contributes to transparency of the research. We present here a formalization of that claim, stating that all things of class “usage of Linked Data Scopes” that are in the context of a thing of class “digital humanities research” can generally have a relation of type “contributes to” to a thing of class “transparency” in the same context.

1. Introduction

De Boer et al. [1] state that “With the rise of data driven methods in the humanities, it becomes necessary to develop reusable and consistent methodological patterns for dealing with the various data manipulation steps. This increases transparency, replicability of the research. Data scopes present a qualitative framework for such methodological steps. In this work we present a Linked Data model to represent and share Data Scopes”. We present here a formalization of the main scientific claim from this quote by using a semantic template called the super-pattern [2].

2. Formalization

Our formalization looks as follows:

CONTEXT-CLASS ("in the context of all ..."):	digital humanities research
SUBJECT-CLASS ("things of type ..."):	usage of Linked Data Scopes
QUALIFIER:	can generally
RELATION-TYPE ("have a relation of type..."):	contributes to
OBJECT-CLASS ("to things of type..."):	transparency

In the context class we use a new minted class "digital humanities research" that is a subclass of "research" (Q42240) from Wikidata and is related to the class "digital humanities" (Q1026962) from Wikidata. In the subject class, we use a new minted class "usage of Linked Data Scopes" that is defined using the restriction "some values from" from OWL on "the Data Scopes Ontology" with the property "uses" (P2283) from Wikidata. In the object class we use the class "transparency" (Q535347) from Wikidata.

3. RDF Code

This is our formalization as a nanopublication in TriG format:

```
@prefix this: <http://purl.org/np/RAgoIxfXPqNDY8vnK2EmBQDAFwuFIDJtfaMplTvPMq3pg> .
@prefix sub: <http://purl.org/np/RAgoIxfXPqNDY8vnK2EmBQDAFwuFIDJtfaMplTvPMq3pg#> .
@prefix np: <http://www.nanopub.org/nschema#> .
@prefix dct: <http://purl.org/dc/terms/> .
@prefix nt: <https://w3id.org/np/o/ntemplate/> .
@prefix npx: <http://purl.org/nanopub/x/> .
@prefix xsd: <http://www.w3.org/2001/XMLSchema#> .
@prefix rdfs: <http://www.w3.org/2000/01/rdf-schema#> .
@prefix orcid: <https://orcid.org/> .
@prefix prov: <http://www.w3.org/ns/prov#> .
@prefix sp: <https://w3id.org/linkflows/superpattern/terms/> .

sub:Head {
  this: np:hasAssertion sub:assertion ;
    np:hasProvenance sub:provenance ;
    np:hasPublicationInfo sub:pubinfo ;
    a np:Nanopublication .
}
sub:assertion {
  sub:spi a sp:SuperPatternInstance ;
    rdfs:label "In the context of Digital Humanities research, usage of the Linked Data Scopes ontology contributes to
transparency of the research." ;
    sp:hasContextClass <http://purl.org/np/RAkCjYmMU3obIrC4IpwUw84JW1ymd312yz0N0g-R9yes0#digital-humanities-research> ;
    sp:hasSubjectClass <http://purl.org/np/RAcPa1aO8kAt8QYwjQoJq-PIzYvo0jCzYrAiOX_XOyk1w#usage-of-linked-data-scopes> ;
    sp:hasQualifier sp:canGenerallyQualifier ;
    sp:hasRelation sp:contributesTo ;
    sp:hasObjectClass <https://www.wikidata.org/wiki/Q535347> .
}
sub:provenance {
  sub:activity a sp:FormalizationActivity ;
    prov:used sub:quote , <https://doi.org/10.1007%2F978-3-030-71903-6_32> ;
    prov:wasAssociatedWith orcid:0000-0001-9079-039X .
  sub:assertion prov:wasGeneratedBy sub:activity .
  sub:quote prov:value "With the rise of data driven methods in the humanities, it becomes necessary to develop reusable and
consistent methodological patterns for dealing with the various data manipulation steps. This increases transparency,
```

```
replicability of the research. Data scopes present a qualitative framework for such methodological steps. In this work we present
a Linked Data model to represent and share Data Scopes" ;
    prov:wasQuotedFrom <https://doi.org/10.1007%2F978-3-030-71903-6_32> .
}
sub:pubinfo {
  sub:sig npx:hasAlgorithm "RSA" ;
    npx:hasPublicKey
"MIGfMA0GCSqGSIb3DQEBAQUAA4GNADCBiQKBgQCUtUS5tVuMGtBUJ+dpxk0EKcI3xKBG4efTcKjAMHeQj6b+liabAld6kXxQKQIRVQomBKOcvoYJ9oTC4K4AMUPMfQsP
SLt1noFxSahw86AXdAh7kWiVZu04Vs0NDuPPUOWwRQjXRf6gYFLQu3gA5o0PkWd7FZ6lrum6lNmi/LFQdwIDAQAB" ;
    npx:hasSignature
"Zn7f/GSB5lE7q1x9oYoWH2jMAnMIGJhh5bdy6spvltV7O2OWok82jK7IcZoHdtFrzFcMKyrb18VhXJD6KY1NFP2zui5n0gyBNzv/aruPWUdTx8WaijNQv75R96ZmUHIr
WknF8lpF53HjGK1TfIVUevYvOlNFv88DiQdMo9naSPM=" ;
    npx:hasSignatureTarget this: .
  this: dct:created "2021-11-19T09:25:07.665+01:00"^^xsd:dateTime ;
    dct:creator orcid:0000-0001-9079-039X ;
    npx:introduces sub:spi ;
    npx:supersedes <http://purl.org/np/RA0HU7k7XUj81ioBftZFk7ywHx5uBBlVgXduglUYgyW5w> ;
    <https://w3id.org/linkflows/reviews/isUpdateOf> <http://purl.org/np/RATc9O9tCdQPlrsJeGNsTNO0PbENIzdKa6XAOex7dXfrY> ;
    nt:wasCreatedFromProvenanceTemplate <http://purl.org/np/RAB_oy10D3XUP-zYlqGz7Uj58AsUXhEKeGqmRFg5LSgDM> ;
    nt:wasCreatedFromPubinfoTemplate <http://purl.org/np/RA2vCBXZf-icEcVRGhulJXugTGxpsV5yVr9yqCI1bQh4A> ,
<http://purl.org/np/RAA2MfqdBCzmz9yVWjKLXNbyfBNcwsMmOqcNUxkk1maIM> ,
<http://purl.org/np/RAjpBMlw3owYhJUBo3DtsuDlXsNAJ8cnGeWAutDVjuAuI> ;
    nt:wasCreatedFromTemplate <http://purl.org/np/RAv68imZrEjfcp2rnEg1hzoBqEVc0cQMtp9_1Za0BxNM4> .
}
```

The following nanopublications introduce the newly minted classes in TriG format.

This is the class definition of “digital humanities research”:

```
@prefix this: <http://purl.org/np/RAkCjYmMU3obIrC4IpwUw84JW1ymd312yz0N0g-R9yes0> .
@prefix sub: <http://purl.org/np/RAkCjYmMU3obIrC4IpwUw84JW1ymd312yz0N0g-R9yes0#> .
@prefix np: <http://www.nanopub.org/nschema#> .
@prefix dct: <http://purl.org/dc/terms/> .
@prefix nt: <https://w3id.org/np/o/ntemplate/> .
@prefix npx: <http://purl.org/nanopub/x/> .
@prefix xsd: <http://www.w3.org/2001/XMLSchema#> .
@prefix rdfs: <http://www.w3.org/2000/01/rdf-schema#> .
@prefix orcid: <https://orcid.org/> .
@prefix prov: <http://www.w3.org/ns/prov#> .
@prefix skos: <http://www.w3.org/2004/02/skos/core#> .

sub:Head {
  this: np:hasAssertion sub:assertion ;
    np:hasProvenance sub:provenance ;
    np:hasPublicationInfo sub:pubinfo ;
    a np:Nanopublication .
}
sub:assertion {
  sub:digital-humanities-research a <http://www.w3.org/2002/07/owl#Class> ;
    rdfs:label "Digital Humanities Research" ;
    rdfs:subClassOf <http://www.wikidata.org/entity/Q42240> ;
    skos:definition "systematic study undertaken to increase knowledge in the field of Digital Humanities" ;
    skos:relatedMatch <http://www.wikidata.org/entity/Q1026962> .
}
sub:provenance {
  sub:assertion prov:wasAttributedTo orcid:0000-0001-9079-039X .
}
sub:pubinfo {
  sub:sig npx:hasAlgorithm "RSA" ;
    npx:hasPublicKey
"MIGfMA0GCSqGSIb3DQEBAQUAA4GNADCBiQKBgQCUtUS5tVuMGtBUJ+dpxk0EKcI3xKBG4efTcKjAMHeQj6b+liabAld6kXxQKQIRVQomBKOcvoYJ9oTC4K4AMUPMfQsP
SLt1noFxSahw86AXdAh7kWiVZu04Vs0NDuPPUOWwRQjXRf6gYFLQu3gA5o0PkWd7FZ6lrum6lNmi/LFQdwIDAQAB" ;
    npx:hasSignature
"iXaNMYj4CT3YA+x8F4yuNplPhG8xjxeAAep0/7CYZS2LQlD2ovNJMvJCSxqLp6qhraPpSo9hnWeS/zLL1TwMfT4W0KtjQnET8mqI2kpqC9XYllcch2jr2sVPgiL7YgR9
mir9CzEXHRchU8m6QH52twnsfQWtN3uLo+rOl2hMmJs=" ;
    npx:hasSignatureTarget this: .
  this: dct:created "2021-10-26T14:11:45.332+02:00"^^xsd:dateTime ;
    dct:creator orcid:0000-0001-9079-039X ;
    npx:introduces sub:digital-humanities-research ;
    nt:wasCreatedFromProvenanceTemplate <http://purl.org/np/RANwQa4ICWS5SOjw7gp99nBpXBasapwtZF1fIM3H2gYTM> ;
    nt:wasCreatedFromPubinfoTemplate <http://purl.org/np/RAA2MfqdBCzmz9yVWjKLXNbyfBNcwsMmOqcNUxkk1maIM> ;
    nt:wasCreatedFromTemplate <http://purl.org/np/RAdpgRpigXtt8iPV9uOPf3wIT3qzOI8Sg2Q72CNV8g-Yo> .
}
```

This is the class definition of “usage of Linked Data Scopes”:

```
@prefix this: <http://purl.org/np/RAcPa1aO8kAt8QYwjQoJq-PIzYvo0jCzYrAiOX_XOyk1w> .
@prefix sub: <http://purl.org/np/RAcPa1aO8kAt8QYwjQoJq-PIzYvo0jCzYrAiOX_XOyk1w#> .
@prefix np: <http://www.nanopub.org/nschema#> .
@prefix dct: <http://purl.org/dc/terms/> .
@prefix nt: <https://w3id.org/np/o/ntemplate/> .
@prefix npx: <http://purl.org/nanopub/x/> .
@prefix xsd: <http://www.w3.org/2001/XMLSchema#> .
@prefix rdfs: <http://www.w3.org/2000/01/rdf-schema#> .
@prefix orcid: <https://orcid.org/> .
@prefix prov: <http://www.w3.org/ns/prov#> .
@prefix owl: <http://www.w3.org/2002/07/owl#> .

sub:Head {
  this: np:hasAssertion sub:assertion ;
    np:hasProvenance sub:provenance ;
    np:hasPublicationInfo sub:pubinfo ;
    a np:Nanopublication .
}
sub:assertion {
  sub:usage-of-linked-data-scopes dct:description "Usage of the Linked Data Scopes ontology in a research project" ;
    a owl:Class , owl:Restriction ;
    rdfs:label "Usage of the Linked Data Scopes ontology" ;
    owl:onProperty <http://wikidata.org/prop/direct/P2283> ;
    owl:someValuesFrom <https://w3id.org/datascope/> .
}
sub:provenance {
  sub:assertion prov:wasAttributedTo orcid:0000-0001-9079-039X .
}
sub:pubinfo {
  sub:sig npx:hasAlgorithm "RSA" ;
    npx:hasPublicKey
"MIGfMA0GCSqGSIb3DQEBAQUAA4GNADCBiQKBgQCUtUS5tVuMGtBUJ+dpxk0EKcI3xKBG4efTcKjAMHeQj6b+liabAld6kXxQKQIRVQomBKOcvoYJ9oTC4K4AMUPMfQsP
SLt1noFxSahw86AXdAh7kWiVZu04Vs0NDuPPUOWwRQjXRf6gYFLQu3gA5o0PkWd7FZ6lrum6lNmi/LFQdwIDAQAB" ;
    npx:hasSignature
"CrXMFJLR+Q5CY5wK6uM5TWBFMfb+S3RR77LhqkIUcjwml9ARVAixA6iAyRqUAo/Of3vpJdzPzjoGbieJGYLhbPfn3bVC70Zwzwv1S/+g8jtrNmshNR1ZBsQ/D5zkLHjE
dXx52VP+Sqmg4YTQsm7oOTtKu9jZfJo33MS9sogr8B4=" ;
    npx:hasSignatureTarget this: .
  this: dct:created "2021-11-19T09:23:19.603+01:00"^^xsd:dateTime ;
    dct:creator orcid:0000-0001-9079-039X ;
    npx:introduces sub:usage-of-linked-data-scopes ;
    npx:supersedes <http://purl.org/np/RAUxfk7XeLxn-2hiX3xWSlE5LXgkNW0MIFBGe2h3Bb3pM> ;
    nt:wasCreatedFromProvenanceTemplate <http://purl.org/np/RANwQa4ICWS5SOjw7gp99nBpXBasapwtZF1fIM3H2gYTM> ;
    nt:wasCreatedFromPubinfoTemplate <http://purl.org/np/RAA2MfqdBCzmz9yVWjKLXNbyfBNcwsMmOqcNUxkk1maIM> ,
<http://purl.org/np/RAjpBMlw3owYhJUBo3DtsuDlXsNAJ8cnGeWAutDVjuAuI> ;
    nt:wasCreatedFromTemplate <http://purl.org/np/RAX7opQ5Tv_uIMSyQNujbJFJO4JLa-xuTUitQlw2aQ1eU> .
}
```

References

[1] de Boer V., Bonestroo I., Koolen M., Hoekstra R. (2021) A Linked Data Model for Data Scopes. In: Garoufallou E., Ovalle-Perandones MA. (eds) Metadata and Semantic Research. MTSR 2020. Communications in Computer and Information Science, vol 1355. Springer, Cham. doi: 10.1007/978-3-030-71903-6_32.

[2] Bucur, C.I., Kuhn, T., Ceolin, D., Ossenbruggen, J. van. Expressing high-level scientific claims with formal semantics. In: Proceedings of the 11th Knowledge Capture Conference 2021. doi: 10.1145/3460210.3493561.
