# Supplementary material for: Nanopublication-based semantic publishing and reviewing: a field study with formalization papers
Source: PeerJ Comput Sci. 2023 Feb 21;9:e1159. doi: 10.7717/peerj-cs.1159 (PMC10280262; doi:10.7717/peerj-cs.1159)
Supplement: Supplemental Information 2 [file peerj-cs-09-1159-s002.zip › formalization_papers_supplemental-main/accepted_submissions/s1_Amelia_Joslin.docx]

**Title:** A formalization of one of the main claims of “FTO Obesity Variant Circuitry and Adipocyte Browning in Humans” by Claussnitzer et al. 2015

**Authors:** Amelia Joslin, ORCID: 0000-0003-2432-4125

**Affiliations:** Maze Therapeutics, USA. E-mail: [ajoslin@mazetx.com](mailto:ajoslin@mazetx.com)

**Keywords:** “early human adipogenesis”, “regulatory element within the first intron of FTO”, “expression of genes IRX3 and IRX5”

**Article Type:** Formalization Paper

**As RDF/nanopublication:** <http://purl.org/np/RAxBBJ2WkonyQNlXfdCAOaCi64J_xqgVGeaLjVQow9M88>

**Editor:** Cristina-Iulia Bucur, ORCID: 0000-0002-7114-6459

**Review comments from:**

- Mariya Dimitrova, ORCID: 0000-0002-8083-6048
- Tobias Kuhn, ORCID: 0000-0002-1267-0234
- Davide Ceolin, ORCID: 0000-0002-3357-9130
- Cristina-Iulia Bucur, ORCID: 0000-0002-7114-6459

**Received:** 2021-06-21

**Accepted:** 2021-11-12

**Abstract:**

Claussnitzer et al. claimed in previous work that a regulatory element within the first intron of FTO affects IRX3 and IRX5 expression during early adipogenesis. We present here a formalization of that claim, stating that all things of class “early human adipogenesis” that are in the context of a thing of class “regulatory element within the first intron of FTO” generally have a relation of type “affects” to a thing of class “expression of genes IRX3 and IRX5” in the same context.

1. **Introduction**

Claussnitzer et al. [1] state that “The rs1421085 T-to-C single-nucleotide variant disrupts a conserved motif for the ARID5B repressor, which leads to derepression of a potent preadipocyte enhancer and a doubling of IRX3 and IRX5 expression during early adipocyte differentiation.”. We present here a formalization of the main scientific claim from this quote by using a semantic template called the super-pattern [2].

1. **Formalization**

Our formalization looks as follows:

| CONTEXT-CLASS (“in the context of all ..."): | [early human adipogenesis](http://purl.org/np/RAtsHwzNs36rGrLnoSbGrPD351Qw033Acoe4zmdXhsYlM#early-human-adipogenesis) |
| --- | --- |
| SUBJECT-CLASS (“things of type ..."): | [regulatory element within the first intron of FTO](http://purl.org/np/RAxLYvJ1JrRf2JAowYGbGJleQPmqtpXnXsIvse7GmLeT8#regulatory-element-within-the-first-intron-of-FTO) |
| QUALIFIER: | [generally](https://w3id.org/linkflows/superpattern/terms/generallyQualifier) |
| RELATION-TYPE (“have a relation of type...”): | [affects](https://w3id.org/linkflows/superpattern/terms/affects) |
| OBJECT-CLASS (“to things of type...”): | [expression of genes IRX3 and IRX5](http://purl.org/np/RAwkXiTv7qCtqOYzlR6ozZRGLRtG6mlogrYdRQ1E4dRDg#expression-of-genes-IRX3-and-IRX5) |

In the context class we use a new minted class “early human adipogenesis” that is a subclass of “adipogenesis” (Q2824461) from Wikidata. In the subject class, we use a new minted class “regulatory element within the first intron of FTO” that is related to the class “FTO” (Q14912501) and “intron” (Q207551) from Wikidata. In the object class we minted a new class “expression of genes IRX3 and IRX5” that is a subclass of “gene expression” (Q26972) from Wikidata and is related to the class “IRX5” (Q18035174) and “IRX3” (Q18046058) from Wikidata.

1. **RDF Code**

This is our formalization as a nanopublication in TriG format:

@prefix this: <http://purl.org/np/RAxBBJ2WkonyQNlXfdCAOaCi64J_xqgVGeaLjVQow9M88> .

@prefix sub: <http://purl.org/np/RAxBBJ2WkonyQNlXfdCAOaCi64J_xqgVGeaLjVQow9M88#> .

@prefix np: <http://www.nanopub.org/nschema#> .

@prefix dct: <http://purl.org/dc/terms/> .

@prefix nt: <https://w3id.org/np/o/ntemplate/> .

@prefix npx: <http://purl.org/nanopub/x/> .

@prefix xsd: <http://www.w3.org/2001/XMLSchema#> .

@prefix rdfs: <http://www.w3.org/2000/01/rdf-schema#> .

@prefix orcid: <https://orcid.org/> .

@prefix prov: <http://www.w3.org/ns/prov#> .

@prefix sp: <https://w3id.org/linkflows/superpattern/terms/> .

sub:Head {

this: np:hasAssertion sub:assertion ;

np:hasProvenance sub:provenance ;

np:hasPublicationInfo sub:pubinfo ;

a np:Nanopublication .

}

sub:assertion {

sub:spi a sp:SuperPatternInstance ;

rdfs:label "A regulatory element within the first intron of FTO affects IRX3 and IRX5 expression during early adipogenesis" ;

sp:hasContextClass <http://purl.org/np/RAtsHwzNs36rGrLnoSbGrPD351Qw033Acoe4zmdXhsYlM#early-human-adipogenesis> ;

sp:hasSubjectClass <http://purl.org/np/RAxLYvJ1JrRf2JAowYGbGJleQPmqtpXnXsIvse7GmLeT8#regulatory-element-within-the-first-intron-of-FTO> ;

sp:hasQualifier sp:generallyQualifier ;

sp:hasRelation sp:affects ;

sp:hasObjectClass <http://purl.org/np/RAwkXiTv7qCtqOYzlR6ozZRGLRtG6mlogrYdRQ1E4dRDg#expression-of-genes-IRX3-and-IRX5> .

}

sub:provenance {

sub:activity a sp:FormalizationActivity ;

prov:used sub:quote , <https://doi.org/10.1056/NEJMoa1502214> ;

prov:wasAssociatedWith orcid:0000-0003-2432-4125 .

sub:assertion prov:wasGeneratedBy sub:activity .

sub:quote prov:value "The rs1421085 T-to-C single-nucleotide variant disrupts a conserved motif for the ARID5B repressor, which leads to derepression of a potent preadipocyte enhancer and a doubling of IRX3 and IRX5 expression during early adipocyte differentiation." ;

prov:wasQuotedFrom <https://doi.org/10.1056/NEJMoa1502214> .

}

sub:pubinfo {

sub:sig npx:hasAlgorithm "RSA" ;

npx:hasPublicKey "MIGfMA0GCSqGSIb3DQEBAQUAA4GNADCBiQKBgQCS1s1OiABv1mrDnFWp9ecwHdQ+UlJn6JCDYXspRsbZgsm2QAiofbnVvf6gfkcjjScd5fq3sW/W8r/o7bdafVSd6xuTG/vHrO9Ax/guPiJ6O/Z57Hk0nQy6c+l71HpaJ2BPPWKOgYiFZ/8eulmuM6BJ8TDKxoBtcqP5QbvN9KYjlQIDAQAB" ;

npx:hasSignature "MUs9dML4QrzQVnfT2hqnfkif+lpusIVT0o4c74M69kgNQmA0CXVRzUZIHcvavS/pMlobVFBhfkTQ7kHs5a0CZGVrvtOhQJYD17eXzg6dwD/LqdYNYErvWxduyvwInXMasXpDZdYkFtddAFt/5Vny6BSBoiIzclnGfGwahZhFmpg=" ;

npx:hasSignatureTarget this: .

this: dct:created "2021-10-27T10:22:03.671-07:00"^^xsd:dateTime ;

dct:creator orcid:0000-0003-2432-4125 ;

npx:introduces sub:spi ;

<https://w3id.org/linkflows/reviews/isUpdateOf> <http://purl.org/np/RAxxJWyH8OdTJLs-ZY1q5jVq1OWFNOoLFvwEcUFyR8EEo> ;

nt:wasCreatedFromProvenanceTemplate <http://purl.org/np/RAB_oy10D3XUP-zYlqGz7Uj58AsUXhEKeGqmRFg5LSgDM> ;

nt:wasCreatedFromPubinfoTemplate <http://purl.org/np/RAA2MfqdBCzmz9yVWjKLXNbyfBNcwsMmOqcNUxkk1maIM> , <http://purl.org/np/RAOGu9Lh0BD4tbIRB9RG6RGRA_ObDh75NTbIqaWgxxs8M> ;

nt:wasCreatedFromTemplate <http://purl.org/np/RAv68imZrEjfcp2rnEg1hzoBqEVc0cQMtp9_1Za0BxNM4> .

}

The following nanopublications introduce the newly minted classes in TriG format.

This is the class definition of “early human adipogenesis”:

@prefix this: <http://purl.org/np/RAtsHwzNs36rGrLnoSbGrPD351Qw033Acoe4zmdXhsYlM> .

@prefix sub: <http://purl.org/np/RAtsHwzNs36rGrLnoSbGrPD351Qw033Acoe4zmdXhsYlM#> .

@prefix np: <http://www.nanopub.org/nschema#> .

@prefix dct: <http://purl.org/dc/terms/> .

@prefix nt: <https://w3id.org/np/o/ntemplate/> .

@prefix npx: <http://purl.org/nanopub/x/> .

@prefix xsd: <http://www.w3.org/2001/XMLSchema#> .

@prefix rdfs: <http://www.w3.org/2000/01/rdf-schema#> .

@prefix orcid: <https://orcid.org/> .

@prefix prov: <http://www.w3.org/ns/prov#> .

@prefix skos: <http://www.w3.org/2004/02/skos/core#> .

sub:Head {

this: np:hasAssertion sub:assertion ;

np:hasProvenance sub:provenance ;

np:hasPublicationInfo sub:pubinfo ;

a np:Nanopublication .

}

sub:assertion {

sub:early-human-adipogenesis a <http://www.w3.org/2002/07/owl#Class> ;

rdfs:label "early human adipogenesis" ;

rdfs:subClassOf <http://www.wikidata.org/entity/Q2824461> ;

skos:definition "early human adipogenesis" .

}

sub:provenance {

sub:assertion prov:wasAttributedTo orcid:0000-0003-2432-4125 .

}

sub:pubinfo {

sub:sig npx:hasAlgorithm "RSA" ;

npx:hasPublicKey "MIGfMA0GCSqGSIb3DQEBAQUAA4GNADCBiQKBgQCS1s1OiABv1mrDnFWp9ecwHdQ+UlJn6JCDYXspRsbZgsm2QAiofbnVvf6gfkcjjScd5fq3sW/W8r/o7bdafVSd6xuTG/vHrO9Ax/guPiJ6O/Z57Hk0nQy6c+l71HpaJ2BPPWKOgYiFZ/8eulmuM6BJ8TDKxoBtcqP5QbvN9KYjlQIDAQAB" ;

npx:hasSignature "eiaG+BcxT16aKpKUpVosKrOxB6PON3szX55WGjEnlAwV87u5mA4huRHXOoDdC/4XgPvT40woR34zhB+VQvLJoizmk6rTPEVReNSG8vdxq3Wdbj6W4qJszBlmxb+XFw2Awdj/4IzNIzK1/at9WQvIE5rV8tkYIvSnmLfWTxKvL/A=" ;

npx:hasSignatureTarget this: .

this: dct:created "2021-06-21T16:44:19.998-07:00"^^xsd:dateTime ;

dct:creator orcid:0000-0003-2432-4125 ;

npx:introduces sub:early-human-adipogenesis ;

nt:wasCreatedFromProvenanceTemplate <http://purl.org/np/RANwQa4ICWS5SOjw7gp99nBpXBasapwtZF1fIM3H2gYTM> ;

nt:wasCreatedFromPubinfoTemplate <http://purl.org/np/RAA2MfqdBCzmz9yVWjKLXNbyfBNcwsMmOqcNUxkk1maIM> ;

nt:wasCreatedFromTemplate <http://purl.org/np/RAdpgRpigXtt8iPV9uOPf3wIT3qzOI8Sg2Q72CNV8g-Yo> .

}

This is the class definition of “regulatory element within the first intron of FTO”:

@prefix this: <http://purl.org/np/RAxLYvJ1JrRf2JAowYGbGJleQPmqtpXnXsIvse7GmLeT8> .

@prefix sub: <http://purl.org/np/RAxLYvJ1JrRf2JAowYGbGJleQPmqtpXnXsIvse7GmLeT8#> .

@prefix np: <http://www.nanopub.org/nschema#> .

@prefix dct: <http://purl.org/dc/terms/> .

@prefix nt: <https://w3id.org/np/o/ntemplate/> .

@prefix npx: <http://purl.org/nanopub/x/> .

@prefix xsd: <http://www.w3.org/2001/XMLSchema#> .

@prefix rdfs: <http://www.w3.org/2000/01/rdf-schema#> .

@prefix orcid: <https://orcid.org/> .

@prefix prov: <http://www.w3.org/ns/prov#> .

@prefix skos: <http://www.w3.org/2004/02/skos/core#> .

sub:Head {

this: np:hasAssertion sub:assertion ;

np:hasProvenance sub:provenance ;

np:hasPublicationInfo sub:pubinfo ;

a np:Nanopublication .

}

sub:assertion {

sub:regulatory-element-within-the-first-intron-of-FTO a <http://www.w3.org/2002/07/owl#Class> ;

rdfs:label "regulatory element within the first intron of FTO" ;

skos:definition "a regulatory element present within the first intron of the human gene FTO" ;

skos:relatedMatch <http://www.wikidata.org/entity/Q14912501> , <http://www.wikidata.org/entity/Q207551> .

}

sub:provenance {

sub:assertion prov:wasAttributedTo orcid:0000-0003-2432-4125 .

}

sub:pubinfo {

sub:sig npx:hasAlgorithm "RSA" ;

npx:hasPublicKey "MIGfMA0GCSqGSIb3DQEBAQUAA4GNADCBiQKBgQCS1s1OiABv1mrDnFWp9ecwHdQ+UlJn6JCDYXspRsbZgsm2QAiofbnVvf6gfkcjjScd5fq3sW/W8r/o7bdafVSd6xuTG/vHrO9Ax/guPiJ6O/Z57Hk0nQy6c+l71HpaJ2BPPWKOgYiFZ/8eulmuM6BJ8TDKxoBtcqP5QbvN9KYjlQIDAQAB" ;

npx:hasSignature "M8cVwUkflLAuLqLUM+ul7EdLSXQhcSsbERJUcBwTF2Ri78XD6JNdHsF5+Qekg3xefqy7d8530m2RHPxjSkooNi3XAOZWsd2T6OZiZ0lSmAIsaRarmsnHbl/GDf0OS4OiqCL1jVFNXMosdVd9runltov655znbpiFBJxmMd66+v0=" ;

npx:hasSignatureTarget this: .

this: dct:created "2021-10-27T10:04:01.217-07:00"^^xsd:dateTime ;

dct:creator orcid:0000-0003-2432-4125 ;

npx:introduces sub:regulatory-element-within-the-first-intron-of-FTO ;

npx:supersedes <http://purl.org/np/RANAxL2OxGmP9VAN6cUi_0KEK0rJb9bPn4l1A5HquXKrI> ;

<https://w3id.org/linkflows/reviews/isUpdateOf> <http://purl.org/np/RANAxL2OxGmP9VAN6cUi_0KEK0rJb9bPn4l1A5HquXKrI> ;

nt:wasCreatedFromProvenanceTemplate <http://purl.org/np/RANwQa4ICWS5SOjw7gp99nBpXBasapwtZF1fIM3H2gYTM> ;

nt:wasCreatedFromPubinfoTemplate <http://purl.org/np/RAA2MfqdBCzmz9yVWjKLXNbyfBNcwsMmOqcNUxkk1maIM> , <http://purl.org/np/RAOGu9Lh0BD4tbIRB9RG6RGRA_ObDh75NTbIqaWgxxs8M> , <http://purl.org/np/RAjpBMlw3owYhJUBo3DtsuDlXsNAJ8cnGeWAutDVjuAuI> ;

nt:wasCreatedFromTemplate <http://purl.org/np/RAdpgRpigXtt8iPV9uOPf3wIT3qzOI8Sg2Q72CNV8g-Yo> .

}

This is the class definition of “expression of genes IRX3 and IRX5”:

@prefix this: <http://purl.org/np/RAwkXiTv7qCtqOYzlR6ozZRGLRtG6mlogrYdRQ1E4dRDg> .

@prefix sub: <http://purl.org/np/RAwkXiTv7qCtqOYzlR6ozZRGLRtG6mlogrYdRQ1E4dRDg#> .

@prefix np: <http://www.nanopub.org/nschema#> .

@prefix dct: <http://purl.org/dc/terms/> .

@prefix nt: <https://w3id.org/np/o/ntemplate/> .

@prefix npx: <http://purl.org/nanopub/x/> .

@prefix xsd: <http://www.w3.org/2001/XMLSchema#> .

@prefix rdfs: <http://www.w3.org/2000/01/rdf-schema#> .

@prefix orcid: <https://orcid.org/> .

@prefix prov: <http://www.w3.org/ns/prov#> .

@prefix skos: <http://www.w3.org/2004/02/skos/core#> .

sub:Head {

this: np:hasAssertion sub:assertion ;

np:hasProvenance sub:provenance ;

np:hasPublicationInfo sub:pubinfo ;

a np:Nanopublication .

}

sub:assertion {

sub:expression-of-genes-IRX3-and-IRX5 a <http://www.w3.org/2002/07/owl#Class> ;

rdfs:label "expression of genes IRX3 and IRX5" ;

rdfs:subClassOf <http://www.wikidata.org/entity/Q26972> ;

skos:definition "Expression of the human genes IRX3 and IRX5" ;

skos:relatedMatch <http://www.wikidata.org/entity/Q18035174> , <http://www.wikidata.org/entity/Q18046058> .

}

sub:provenance {

sub:assertion prov:wasAttributedTo orcid:0000-0003-2432-4125 .

}

sub:pubinfo {

sub:sig npx:hasAlgorithm "RSA" ;

npx:hasPublicKey "MIGfMA0GCSqGSIb3DQEBAQUAA4GNADCBiQKBgQCS1s1OiABv1mrDnFWp9ecwHdQ+UlJn6JCDYXspRsbZgsm2QAiofbnVvf6gfkcjjScd5fq3sW/W8r/o7bdafVSd6xuTG/vHrO9Ax/guPiJ6O/Z57Hk0nQy6c+l71HpaJ2BPPWKOgYiFZ/8eulmuM6BJ8TDKxoBtcqP5QbvN9KYjlQIDAQAB" ;

npx:hasSignature "kT9nlq5nE5y03RkSemozwUYzyNGdKTIPvY7WlWiDD2ZlRrHlOxQPbq+VPMEV6Ay3oEgTi/WuE5o6AY+dGK4V6eLcVnDQ1xqC7GsnaD5JGL89a5Z/oEkHf1JG8fHQFOATrg4n62hcjGeRZOZrZLjdnAozGdDQf19gxq78E4QTyOY=" ;

npx:hasSignatureTarget this: .

this: dct:created "2021-10-27T09:44:46.276-07:00"^^xsd:dateTime ;

dct:creator orcid:0000-0003-2432-4125 ;

npx:introduces sub:expression-of-genes-IRX3-and-IRX5 ;

npx:supersedes <http://purl.org/np/RANjcIDg1VkGWDSqxkFyVn__2UEbR-V7y8lqtV4rxcLJk> ;

<https://w3id.org/linkflows/reviews/isUpdateOf> <http://purl.org/np/RAY3LaUoVtBLj9CN36wyyvTIjSMsACErVtEApnA12zjy0> ;

nt:wasCreatedFromProvenanceTemplate <http://purl.org/np/RANwQa4ICWS5SOjw7gp99nBpXBasapwtZF1fIM3H2gYTM> ;

nt:wasCreatedFromPubinfoTemplate <http://purl.org/np/RA2vCBXZf-icEcVRGhulJXugTGxpsV5yVr9yqCI1bQh4A> , <http://purl.org/np/RAA2MfqdBCzmz9yVWjKLXNbyfBNcwsMmOqcNUxkk1maIM> , <http://purl.org/np/RAjpBMlw3owYhJUBo3DtsuDlXsNAJ8cnGeWAutDVjuAuI> ;

nt:wasCreatedFromTemplate <http://purl.org/np/RAdpgRpigXtt8iPV9uOPf3wIT3qzOI8Sg2Q72CNV8g-Yo> .

}

**References**

[1] Claussnitzer, Melina and Dankel, Simon N. and Kim, Kyoung-Han and Quon, Gerald and Meuleman, Wouter and Haugen, Christine and Glunk, Viktoria and Sousa, Isabel S. and Beaudry, Jacqueline L. and Puviindran, Vijitha and Abdennur, Nezar A. and Liu, Jannel and Svensson, Per-Arne and Hsu, Yi-Hsiang and Drucker, Daniel J. and Mellgren, Gunnar and Hui, Chi-Chung and Hauner, Hans and Kellis, Manolis. FTO Obesity Variant Circuitry and Adipocyte Browning in Humans. New England Journal of Medicine 2015;373(10):895-907. doi: 10.1056/NEJMoa1502214.

[2] Bucur, C.I., Kuhn, T., Ceolin, D., Ossenbruggen, J. van. Expressing high-level scientific claims with formal semantics. In: Proceedings of the 11th Knowledge Capture Conference 2021. doi: 10.1145/3460210.3493561.
