# Supplementary material for: Nanopublication-based semantic publishing and reviewing: a field study with formalization papers
Source: PeerJ Comput Sci. 2023 Feb 21;9:e1159. doi: 10.7717/peerj-cs.1159 (PMC10280262; doi:10.7717/peerj-cs.1159)
Supplement: Supplemental Information 2 [file peerj-cs-09-1159-s002.zip › formalization_papers_supplemental-main/accepted_submissions/s2_Nolan_Nichols.docx]

**Title:** A formalization of one of the main claims of “ALS-implicated protein TDP-43 sustains levels of STMN2, a mediator of motor neuron growth and repair” by Klim et al. 2019

**Authors:** B. Nolan Nichols, ORCID: 0000-0003-1099-3328

**Affiliations:** Maze Therapeutics, USA. E-mail: [nnichols@mazetx.com](mailto:nnichols@mazetx.com) , [nolan.nichols@gmail.com](mailto:nolan.nichols@gmail.com)

**Keywords:** “human motor neuron”, “TAR DNA binding protein”, “transcription of stmn2”

**Article Type:** Formalization Paper

**As RDF/nanopublication:** <http://purl.org/np/RAmG2bXxwkIzARk4Mda-lqZU0RVnkpX7hUHBIPcdLHQUU>

**Editor:** Cristina-Iulia Bucur, ORCID: 0000-0002-7114-6459

**Review comments from:**

- Tobias Kuhn, ORCID: 0000-0002-1267-0234
- Davide Ceolin, ORCID: 0000-0002-3357-9130
- Cristina-Iulia Bucur, ORCID: 0000-0002-7114-6459

**Received:** 2021-10-15

**Accepted:** 2021-11-12

**Abstract:**

Klim et al. claimed in previous work that the protein TDP-43 generally contributes to the transcription of STMN2 in human motor neurons. We present here a formalization of that claim, stating that all things of class “TAR DNA binding protein” that are in the context of a thing of class “human motor neuron” can generally have a relation of type “contributes to” to a thing of class “transcription of stmn2” in the same context.

1. **Introduction**

Klim et al. [1] state that “expression of STMN2, which encodes a microtubule regulator, declined after TDP-43 knockdown and TDP-43 mislocalization as well as in patient-specific motor neurons”. We present here a formalization of the main scientific claim from this quote by using a semantic template called the super-pattern [2].

1. **Formalization**

Our formalization looks as follows:

| CONTEXT-CLASS (“in the context of all ..."): | [human motor neuron](http://www.wikidata.org/entity/Q101404862) |
| --- | --- |
| SUBJECT-CLASS (“things of type ..."): | [TAR DNA binding protein](http://www.wikidata.org/entity/Q21133247) |
| QUALIFIER: | [can generally](https://w3id.org/linkflows/superpattern/terms/canGenerallyQualifier) |
| RELATION-TYPE (“have a relation of type...”): | [contributes to](https://w3id.org/linkflows/superpattern/terms/contributesTo) |
| OBJECT-CLASS (“to things of type...”): | [transcription of stmn2](http://purl.org/np/RAiUYY1dbEDbcsscapEmbMMHsgJmjEJ1yUoNsxZIH1r90#transcription-of-stmn2) |

In the context class we use the class “human motor neuron” (Q101404862) from Wikidata. In the subject class, we use the class “TAR DNA binding protein” (Q21133247) from Wikidata. In the object class we minted a new class “transcription of stmn2” that is a subclass of “transcription” (Q177900) from Wikidata and is related to the class “STMN2” (Q18036104) from Wikidata.

1. **RDF Code**

This is our formalization as a nanopublication in TriG format:

@prefix this: <http://purl.org/np/RAmG2bXxwkIzARk4Mda-lqZU0RVnkpX7hUHBIPcdLHQUU> .

@prefix sub: <http://purl.org/np/RAmG2bXxwkIzARk4Mda-lqZU0RVnkpX7hUHBIPcdLHQUU#> .

@prefix np: <http://www.nanopub.org/nschema#> .

@prefix dct: <http://purl.org/dc/terms/> .

@prefix nt: <https://w3id.org/np/o/ntemplate/> .

@prefix npx: <http://purl.org/nanopub/x/> .

@prefix xsd: <http://www.w3.org/2001/XMLSchema#> .

@prefix rdfs: <http://www.w3.org/2000/01/rdf-schema#> .

@prefix orcid: <https://orcid.org/> .

@prefix prov: <http://www.w3.org/ns/prov#> .

@prefix sp: <https://w3id.org/linkflows/superpattern/terms/>.

sub:Head {

this: np:hasAssertion sub:assertion ;

np:hasProvenance sub:provenance ;

np:hasPublicationInfo sub:pubinfo ;

a np:Nanopublication .

}

sub:assertion {

sub:spi a <https://w3id.org/linkflows/superpattern/terms/SuperPatternInstance> ;

rdfs:label "the protein TDP-43 generally contributes to the transcription of STMN2 in human motor neurons" ;

sp:hasContextClass <http://www.wikidata.org/entity/Q101404862> ;

sp:hasSubjectClass <http://www.wikidata.org/entity/Q21133247> ;

sp:hasQualifier sp:canGenerallyQualifier ;

sp:hasRelation sp:contributesTo ;

sp:hasObjectClass <http://purl.org/np/RAiUYY1dbEDbcsscapEmbMMHsgJmjEJ1yUoNsxZIH1r90#transcription-of-stmn2> .

}

sub:provenance {

sub:activity a sp:FormalizationActivity ;

prov:used sub:quote , <https://doi.org/10.1038/s41593-018-0300-4> ;

prov:wasAssociatedWith orcid:0000-0003-1099-3328 .

sub:assertion prov:wasGeneratedBy sub:activity .

sub:quote prov:value "expression of STMN2, which encodes a microtubule regulator, declined after TDP-43 knockdown and TDP-43 mislocalization as well as in patient-specific motor neurons" ;

prov:wasQuotedFrom <https://doi.org/10.1038/s41593-018-0300-4> .

}

sub:pubinfo {

sub:sig npx:hasAlgorithm "RSA" ;

npx:hasPublicKey "MIGfMA0GCSqGSIb3DQEBAQUAA4GNADCBiQKBgQCWyqaozls49LpqjUqCP7APpHaYeBQB0+Erh4usb4W7fWZRVVybfNg+HynNs2p//7PbfQraV2BEMxNMXF85hDpCjFMt9fQdvnS+zYqCh/p352aCoss3zHjzMp6BE9GhmGvevpGh7eJxSkO9WZ54ld38kq7VrcZQ4Rmy0IlLfk/BdQIDAQAB" ;

npx:hasSignature "L8uNtEZZ3sXEtasw18HmJZSvMaHvtlmGz5ZMHz/Qw4Q3TCv+1eqG/1XalFeFNwrTy715BS9P8ygD0uKRjIcDzONv91pM5I0RwyRT4keBIFHRhN6jzu2LujsdByfQd0VFOfndgH2jaBVg9nZhvDanuZGC0hw5OAXT/E6HygtDoVs=" ;

npx:hasSignatureTarget this: .

this: dct:created "2021-10-20T17:26:34.506Z"^^xsd:dateTime ;

dct:creator orcid:0000-0003-1099-3328 ;

npx:introduces sub:spi ;

<https://w3id.org/linkflows/reviews/isUpdateOf> <http://purl.org/np/RA5rRFy9eDTZSTyVeuTrAO7aCg_JPcq1cVmWWjc-kjFOM> ;

nt:wasCreatedFromProvenanceTemplate <http://purl.org/np/RAE1wniOy0yO39PlK9QkQ-wqbC3q-R2nXraP5huu8W39k> ;

nt:wasCreatedFromPubinfoTemplate <http://purl.org/np/RAA2MfqdBCzmz9yVWjKLXNbyfBNcwsMmOqcNUxkk1maIM> , <http://purl.org/np/RAOGu9Lh0BD4tbIRB9RG6RGRA_ObDh75NTbIqaWgxxs8M> ;

nt:wasCreatedFromTemplate <http://purl.org/np/RAv68imZrEjfcp2rnEg1hzoBqEVc0cQMtp9_1Za0BxNM4> .

}

The following nanopublications introduce the newly minted classes in TriG format.

This is the class definition of “transcription of stmn2”:

@prefix this: <http://purl.org/np/RAiUYY1dbEDbcsscapEmbMMHsgJmjEJ1yUoNsxZIH1r90> .

@prefix sub: <http://purl.org/np/RAiUYY1dbEDbcsscapEmbMMHsgJmjEJ1yUoNsxZIH1r90#> .

@prefix np: <http://www.nanopub.org/nschema#> .

@prefix dct: <http://purl.org/dc/terms/> .

@prefix nt: <https://w3id.org/np/o/ntemplate/> .

@prefix npx: <http://purl.org/nanopub/x/> .

@prefix xsd: <http://www.w3.org/2001/XMLSchema#> .

@prefix rdfs: <http://www.w3.org/2000/01/rdf-schema#> .

@prefix orcid: <https://orcid.org/> .

@prefix prov: <http://www.w3.org/ns/prov#> .

@prefix skos: <http://www.w3.org/2004/02/skos/core#> .

sub:Head {

this: np:hasAssertion sub:assertion ;

np:hasProvenance sub:provenance ;

np:hasPublicationInfo sub:pubinfo ;

a np:Nanopublication .

}

sub:assertion {

sub:transcription-of-stmn2 a <http://www.w3.org/2002/07/owl#Class> ;

rdfs:label "transcription of stmn2" ;

rdfs:subClassOf <http://www.wikidata.org/entity/Q177900> ;

skos:definition "biosynthesis of RNA carried out on a template of STMN2 DNA" ;

skos:relatedMatch <http://www.wikidata.org/entity/Q18036104> .

}

sub:provenance {

sub:assertion prov:wasAttributedTo orcid:0000-0003-1099-3328 .

}

sub:pubinfo {

sub:sig npx:hasAlgorithm "RSA" ;

npx:hasPublicKey "MIGfMA0GCSqGSIb3DQEBAQUAA4GNADCBiQKBgQCWyqaozls49LpqjUqCP7APpHaYeBQB0+Erh4usb4W7fWZRVVybfNg+HynNs2p//7PbfQraV2BEMxNMXF85hDpCjFMt9fQdvnS+zYqCh/p352aCoss3zHjzMp6BE9GhmGvevpGh7eJxSkO9WZ54ld38kq7VrcZQ4Rmy0IlLfk/BdQIDAQAB" ;

npx:hasSignature "DSOLsANesn6/E2TJCxQg3/87KS5SUgXVz2En+wxgK4DwASPkk3pnY3/5Qj144utDYvZZwFBO6Jkk1zUxE28DlZl1XqltyVi6JikVxrDemh0UfesyIaIs0Luq7KXha2mbapYW6VGR/REklYjy+K5cUgGN2PyS3vkNO7j1YsjVMVY=" ;

npx:hasSignatureTarget this: .

this: dct:created "2021-10-15T22:30:19.955Z"^^xsd:dateTime ;

dct:creator orcid:0000-0003-1099-3328 ;

npx:introduces sub:transcription-of-stmn2 ;

nt:wasCreatedFromProvenanceTemplate <http://purl.org/np/RANwQa4ICWS5SOjw7gp99nBpXBasapwtZF1fIM3H2gYTM> ;

nt:wasCreatedFromPubinfoTemplate <http://purl.org/np/RAA2MfqdBCzmz9yVWjKLXNbyfBNcwsMmOqcNUxkk1maIM> ;

nt:wasCreatedFromTemplate <http://purl.org/np/RAdpgRpigXtt8iPV9uOPf3wIT3qzOI8Sg2Q72CNV8g-Yo> .

}

**References**

[1] Klim, J.R., Williams, L.A., Limone, F. et al. ALS-implicated protein TDP-43 sustains levels of STMN2, a mediator of motor neuron growth and repair. Nat Neurosci 22, 167–179 (2019). https://doi.org/10.1038/s41593-018-0300-4.

[2] Bucur, C.I., Kuhn, T., Ceolin, D., Ossenbruggen, J. van. Expressing high-level scientific claims with formal semantics. In: Proceedings of the 11th Knowledge Capture Conference 2021. doi: 10.1145/3460210.3493561.
