# Supplementary material for: Nanopublication-based semantic publishing and reviewing: a field study with formalization papers
Source: PeerJ Comput Sci. 2023 Feb 21;9:e1159. doi: 10.7717/peerj-cs.1159 (PMC10280262; doi:10.7717/peerj-cs.1159)
Supplement: Supplemental Information 2 [file peerj-cs-09-1159-s002.zip › formalization_papers_supplemental-main/accepted_submissions/s2_Nolan_Nichols.pdf]

Title: A formalization of one of the main claims of “ALS-implicated protein TDP-43 sustains levels of STMN2, a mediator of motor neuron growth and repair” by Klim et al. 2019

Authors: B. Nolan Nichols, ORCID: 0000-0003-1099-3328

Affiliations: Maze Therapeutics, USA. E-mail: nnichols@mazetx.com , nolan.nichols@gmail.com

Keywords: “human motor neuron”, “TAR DNA binding protein”, “transcription of stmn2”

Article Type: Formalization Paper

As RDF/nanopublication:
http://purl.org/np/RAmG2bXxwkIzARk4Mda-IqZU0RVnkpX7hUHBIPcdLHQUU

Editor: Cristina-Iulia Bucur, ORCID: 0000-0002-7114-6459

Review comments from:

- Tobias Kuhn, ORCID: 0000-0002-1267-0234
- Davide Ceolin, ORCID: 0000-0002-3357-9130
- Cristina-Iulia Bucur, ORCID: 0000-0002-7114-6459

Received: 2021-10-15

Accepted: 2021-11-12

Abstract:
Klim et al. claimed in previous work that the protein TDP-43 generally contributes to the transcription of STMN2 in human motor neurons. We present here a formalization of that claim, stating that all things of class “TAR DNA binding protein” that are in the context of a thing of class “human motor neuron” can generally have a relation of type “contributes to” to a thing of class “transcription of stmn2” in the same context.

1. Introduction

Klim et al. [1] state that “expression of STMN2, which encodes a microtubule regulator, declined after TDP-43 knockdown and TDP-43 mislocalization as well as in patient-specific motor neurons”. We present here a formalization of the main scientific claim from this quote by using a semantic template called the super-pattern [2].

2. Formalization

Our formalization looks as follows:

CONTEXT-CLASS (“in the context of all ..."):	human motor neuron
SUBJECT-CLASS (“things of type ..."):	TAR DNA binding protein
QUALIFIER:	can generally
RELATION-TYPE (“have a relation of type...”):	contributes to
OBJECT-CLASS (“to things of type...”):	transcription of stmn2

In the context class we use the class “human motor neuron” (Q101404862) from Wikidata. In the subject class, we use the class “TAR DNA binding protein” (Q21133247) from Wikidata. In the object class we minted a new class “transcription of stmn2” that is a subclass of “transcription” (Q177900) from Wikidata and is related to the class “STMN2” (Q18036104) from Wikidata.

3. RDF Code

This is our formalization as a nanopublication in TriG format:

```
@prefix this: <http://purl.org/np/RAmG2bXxwkIzARk4Mda-lqZU0RVnkpX7hUHBIPcdLHQUU> .
@prefix sub: <http://purl.org/np/RAmG2bXxwkIzARk4Mda-lqZU0RVnkpX7hUHBIPcdLHQUU#> .
@prefix np: <http://www.nanopub.org/nschema#> .
@prefix dct: <http://purl.org/dc/terms/> .
@prefix nt: <https://w3id.org/np/o/ntemplate/> .
@prefix npx: <http://purl.org/nanopub/x/> .
@prefix xsd: <http://www.w3.org/2001/XMLSchema#> .
@prefix rdfs: <http://www.w3.org/2000/01/rdf-schema#> .
@prefix orcid: <https://orcid.org/> .
@prefix prov: <http://www.w3.org/ns/prov#> .
@prefix sp: <https://w3id.org/linkflows/superpattern/terms/>.


sub:Head {
  this: np:hasAssertion sub:assertion ;
    np:hasProvenance sub:provenance ;
    np:hasPublicationInfo sub:pubinfo ;
    a np:Nanopublication .
}
sub:assertion {
  sub:spi a <https://w3id.org/linkflows/superpattern/terms/SuperPatternInstance> ;
    rdfs:label "the protein TDP-43 generally contributes to the transcription of STMN2 in human motor neurons" ;
    sp:hasContextClass <http://www.wikidata.org/entity/Q101404862> ;
    sp:hasSubjectClass <http://www.wikidata.org/entity/Q21133247> ;
    sp:hasQualifier sp:canGenerallyQualifier ;
    sp:hasRelation sp:contributesTo ;
    sp:hasObjectClass <http://purl.org/np/RAiUYY1dbEDbcsscapEmbMMHsgJmjEJ1yUoNsxZIH1r90#transcription-of-stmn2> .
}
sub:provenance {
  sub:activity a sp:FormalizationActivity ;
    prov:used sub:quote , <https://doi.org/10.1038/s41593-018-0300-4> ;
    prov:wasAssociatedWith orcid:0000-0003-1099-3328 .
  sub:assertion prov:wasGeneratedBy sub:activity .
  sub:quote prov:value "expression of STMN2, which encodes a microtubule regulator, declined after TDP-43 knockdown and TDP-43
mislocalization as well as in patient-specific motor neurons" ;
    prov:wasQuotedFrom <https://doi.org/10.1038/s41593-018-0300-4> .
}
sub:pubinfo {
  sub:sig npx:hasAlgorithm "RSA" ;
    npx:hasPublicKey
"MIGfMA0GCSqGSIb3DQEBAQUAA4GNADCBiQKBgQCWyqaozls49LpqjUqCP7APpHaYeBQB0+Erh4usb4W7fWZRVVybfNg+HynNs2p//7PbfQraV2BEMxNMXF85hDpCjFMt
9fQdvnS+zYqCh/p352aCoss3zHjzMp6BE9GhmGvevpGh7eJxSkO9WZ54ld38kq7VrcZQ4Rmy0IlLfk/BdQIDAQAB" ;
    npx:hasSignature
"L8uNtEZZ3sXEtasw18HmJZSvMaHvtlmGz5ZMHz/Qw4Q3TCv+1eqG/1XalFeFNwrTy715BS9P8ygD0uKRjIcDzONv91pM5I0RwyRT4keBIFHRhN6jzu2LujsdByfQd0VF
OfndgH2jaBVg9nZhvDanuZGC0hw5OAXT/E6HygtDoVs=" ;
```

```
    npx:hasSignatureTarget this: .
  this: dct:created "2021-10-20T17:26:34.506Z"^^xsd:dateTime ;
    dct:creator orcid:0000-0003-1099-3328 ;
    npx:introduces sub:spi ;
    <https://w3id.org/linkflows/reviews/isUpdateOf> <http://purl.org/np/RA5rRFy9eDTZSTyVeuTrAO7aCg_JPcq1cVmWWjc-kjFOM> ;
    nt:wasCreatedFromProvenanceTemplate <http://purl.org/np/RAE1wniOy0yO39PlK9QkQ-wqbC3q-R2nXraP5huu8W39k> ;
    nt:wasCreatedFromPubinfoTemplate <http://purl.org/np/RAA2MfqdBCzmz9yVWjKLXNbyfBNcwsMmOqcNUxkk1maIM> ,
<http://purl.org/np/RAOGu9Lh0BD4tbIRB9RG6RGRA_ObDh75NTbIqaWgxxs8M> ;
    nt:wasCreatedFromTemplate <http://purl.org/np/RAv68imZrEjfcp2rnEg1hzoBqEVc0cQMtp9_1Za0BxNM4> .
}
```

The following nanopublications introduce the newly minted classes in TriG format.

This is the class definition of “transcription of stmn2”:

```
@prefix this: <http://purl.org/np/RAiUYY1dbEDbcsscapEmbMMHsgJmjEJ1yUoNsxZIH1r90> .
@prefix sub: <http://purl.org/np/RAiUYY1dbEDbcsscapEmbMMHsgJmjEJ1yUoNsxZIH1r90#> .
@prefix np: <http://www.nanopub.org/nschema#> .
@prefix dct: <http://purl.org/dc/terms/> .
@prefix nt: <https://w3id.org/np/o/ntemplate/> .
@prefix npx: <http://purl.org/nanopub/x/> .
@prefix xsd: <http://www.w3.org/2001/XMLSchema#> .
@prefix rdfs: <http://www.w3.org/2000/01/rdf-schema#> .
@prefix orcid: <https://orcid.org/> .
@prefix prov: <http://www.w3.org/ns/prov#> .
@prefix skos: <http://www.w3.org/2004/02/skos/core#> .


sub:Head {
  this: np:hasAssertion sub:assertion ;
    np:hasProvenance sub:provenance ;
    np:hasPublicationInfo sub:pubinfo ;
    a np:Nanopublication .
}
sub:assertion {
  sub:transcription-of-stmn2 a <http://www.w3.org/2002/07/owl#Class> ;
    rdfs:label "transcription of stmn2" ;
    rdfs:subClassOf <http://www.wikidata.org/entity/Q177900> ;
    skos:definition "biosynthesis of RNA carried out on a template of STMN2 DNA" ;
    skos:relatedMatch <http://www.wikidata.org/entity/Q18036104> .
}
sub:provenance {
  sub:assertion prov:wasAttributedTo orcid:0000-0003-1099-3328 .
}
sub:pubinfo {
  sub:sig npx:hasAlgorithm "RSA" ;
    npx:hasPublicKey
"MIGfMA0GCSqGSIb3DQEBAQUAA4GNADCBiQKBgQCWyqaozls49LpqjUqCP7APpHaYeBQB0+Erh4usb4W7fWZRVVybfNg+HynNs2p//7PbfQraV2BEMxNMXF85hDpCjFMt
9fQdvnS+zYqCh/p352aCoss3zHjzMp6BE9GhmGvevpGh7eJxSkO9WZ54ld38kq7VrcZQ4Rmy0IlLfk/BdQIDAQAB" ;
    npx:hasSignature
"DSOLsANesn6/E2TJCxQg3/87KS5SUgXVz2En+wxgK4DwASPkk3pnY3/5Qj144utDYvZZwFBO6Jkk1zUxE28DlZl1XqltyVi6JikVxrDemh0UfesyIaIs0Luq7KXha2mb
apYW6VGR/REklYjy+K5cUgGN2PyS3vkNO7j1YsjVMVY=" ;
    npx:hasSignatureTarget this: .
  this: dct:created "2021-10-15T22:30:19.955Z"^^xsd:dateTime ;
    dct:creator orcid:0000-0003-1099-3328 ;
    npx:introduces sub:transcription-of-stmn2 ;
    nt:wasCreatedFromProvenanceTemplate <http://purl.org/np/RANwQa4ICWS5SOjw7gp99nBpXBasapwtZF1fIM3H2gYTM> ;
    nt:wasCreatedFromPubinfoTemplate <http://purl.org/np/RAA2MfqdBCzmz9yVWjKLXNbyfBNcwsMmOqcNUxkk1maIM> ;
    nt:wasCreatedFromTemplate <http://purl.org/np/RAdpgRpigXtt8iPV9uOPf3wIT3qzOI8Sg2Q72CNV8g-Yo> .
}
```

References

[1] Klim, J.R., Williams, L.A., Limone, F. et al. ALS-implicated protein TDP-43 sustains levels of STMN2, a mediator of motor neuron growth and repair. Nat Neurosci 22, 167–179 (2019). https://doi.org/10.1038/s41593-018-0300-4.

[2] Bucur, C.I., Kuhn, T., Ceolin, D., Ossenbruggen, J. van. Expressing high-level scientific claims with formal semantics. In: Proceedings of the 11th Knowledge Capture Conference 2021. doi: 10.1145/3460210.3493561.
