# Supplementary material for: Nanopublication-based semantic publishing and reviewing: a field study with formalization papers
Source: PeerJ Comput Sci. 2023 Feb 21;9:e1159. doi: 10.7717/peerj-cs.1159 (PMC10280262; doi:10.7717/peerj-cs.1159)
Supplement: Supplemental Information 2 [file peerj-cs-09-1159-s002.zip › formalization_papers_supplemental-main/accepted_submissions/s3_Daniel_Mietchen.docx]

**Title:** A formalization of one of the main claims of “Cortex reorganization of Xenopus laevis eggs in strong static magnetic fields” by Mietchen et al. 2005

**Authors:** Daniel Mietchen, ORCID: 0000-0001-9488-1870

**Affiliations:** Fraunhofer Institute for Biomedical Engineering (IBMT), Sulzbach, Germany. E-mail: [daniel.mietchen@ibmt.fraunhofer.de](mailto:daniel.mietchen@ibmt.fraunhofer.de)

**Keywords:** “dejellied fertilizable stage VI Xenopus laevis oocyte”, “strong static magnetic field”, “cell cortex”

**Article Type:** Formalization Paper

**As RDF/nanopublication:** <http://purl.org/np/RAXVRaFjWDlX5cZcVRXETaEIAx6QAyLK5JCrzDP-yDp9U>

**Editor:** Cristina-Iulia Bucur, ORCID: 0000-0002-7114-6459

**Review comments from:**

- Michel Dumontier, ORCID: 0000-0003-4727-9435
- Tobias Kuhn, ORCID: 0000-0002-1267-0234
- Cristina-Iulia Bucur, ORCID: 0000-0002-7114-6459

**Received:** 2021-07-25

**Accepted:** 2021-11-17

**Abstract:**

Mietchen et al. claimed in previous work that strong static magnetic fields change the cell cortex in dejellied fertilizable stage VI Xenopus lavis oocytes. We present here a formalization of that claim, stating that all things of class “strong static magnetic field” that are in the context of a thing of class “dejellied fertilizable stage VI Xenopus laevis oocyte” generally have a relation of type “affects'' to a thing of class “cell cortex” in the same context.

1. **Introduction**

Mietchen et al. [1] state that “A complex reorganization of cortical pigmentation was found in dejellied eggs as a function of the magnetic field and the field exposure time”. We present here a formalization of the main scientific claim from this quote by using a semantic template called the super-pattern [2].

1. **Formalization**

Our formalization looks as follows:

| CONTEXT-CLASS (“in the context of all ..."): | [dejellied fertilizable stage VI Xenopus laevis oocyte](https://www.wikidata.org/wiki/Q107644116) |
| --- | --- |
| SUBJECT-CLASS (“things of type ..."): | [strong static magnetic field](https://www.wikidata.org/wiki/Q107644241) |
| QUALIFIER: | [generally](https://w3id.org/linkflows/superpattern/terms/generallyQualifier) |
| RELATION-TYPE (“have a relation of type...”): | [affects](https://w3id.org/linkflows/superpattern/terms/affects) |
| OBJECT-CLASS (“to things of type...”): | [cell cortex](https://www.wikidata.org/wiki/Q5058180) |

In the context class, we use the class “dejellied fertilizable stage VI Xenopus laevis oocyte” (Q107644116) from Wikidata. In the subject class, we use the class “strong static magnetic field” (Q107644241) from Wikidata. In the object class, we use the class “cell cortex” (Q5058180) from Wikidata.

1. **RDF Code**

This is our formalization as a nanopublication in TriG format:

@prefix this: <http://purl.org/np/RAXVRaFjWDlX5cZcVRXETaEIAx6QAyLK5JCrzDP-yDp9U> .

@prefix sub: <http://purl.org/np/RAXVRaFjWDlX5cZcVRXETaEIAx6QAyLK5JCrzDP-yDp9U#> .

@prefix np: <http://www.nanopub.org/nschema#> .

@prefix dct: <http://purl.org/dc/terms/> .

@prefix nt: <https://w3id.org/np/o/ntemplate/> .

@prefix npx: <http://purl.org/nanopub/x/> .

@prefix xsd: <http://www.w3.org/2001/XMLSchema#> .

@prefix rdfs: <http://www.w3.org/2000/01/rdf-schema#> .

@prefix orcid: <https://orcid.org/> .

@prefix prov: <http://www.w3.org/ns/prov#> .

@prefix sp: <https://w3id.org/linkflows/superpattern/terms/> .

sub:Head {

this: np:hasAssertion sub:assertion ;

np:hasProvenance sub:provenance ;

np:hasPublicationInfo sub:pubinfo ;

a np:Nanopublication .

}

sub:assertion {

sub:spi a sp:SuperPatternInstance ;

rdfs:label "Strong static magnetic fields change the cell cortex in dejellied fertilizable stage VI Xenopus lavis oocytes." ;

sp:hasContextClass <http://www.wikidata.org/entity/Q107644116> ;

sp:hasSubjectClass <http://www.wikidata.org/entity/Q107644241> ;

sp:hasQualifier sp:generallyQualifier ;

sp:hasRelation sp:affects ;

sp:hasObjectClass <http://www.wikidata.org/entity/Q5058180> .

}

sub:provenance {

sub:activity a sp:FormalizationActivity ;

prov:used sub:quote , <https://doi.org/10.1186/1477-044X-3-2> ;

prov:wasAssociatedWith orcid:0000-0001-9488-1870 .

sub:assertion prov:wasGeneratedBy sub:activity .

sub:quote prov:value "A complex reorganization of cortical pigmentation was found in dejellied eggs as a function of the magnetic field and the field exposure time." ;

prov:wasQuotedFrom <https://doi.org/10.1186/1477-044X-3-2> .

}

sub:pubinfo {

sub:sig npx:hasAlgorithm "RSA" ;

npx:hasPublicKey "MIGfMA0GCSqGSIb3DQEBAQUAA4GNADCBiQKBgQCJlM78d80R+gFMoQB1IG3f7AbqqGOCIv4HmZd1cx1KgEWMUUpPsojFNvx84fC/TltcJ8F8JafnbhDXW2HM2MhdK4yC04ROEV1vIgSzjDicHfiqXvMqdPuMyQp4mmCEY7mUoeEW10mWZqjk+S9TnmiAQbFGcpExP8aosr2aTR7CSQIDAQAB" ;

npx:hasSignature "akW42kGSMe1sO8SU8VqcxrOSssOW3LLBQONsJBvsigKDV8AiBQ/MaR30ve20LhTgtrFQrwB1jA9ZhCy9zrYxyKVCRKvJzovPppGaTyHd8KCeAhsN0ZmSuu2XKUHqbiepzahoPyxX0GdqCox9PS9D6ssFe8WoRHPVRk3Jzwd5k1I=" ;

npx:hasSignatureTarget this: .

this: dct:created "2021-12-17T11:18:24.918+01:00"^^xsd:dateTime ;

dct:creator orcid:0000-0001-9488-1870 , orcid:0000-0002-7114-6459 ;

npx:introduces sub:spi ;

<https://w3id.org/linkflows/reviews/isUpdateOf> <http://purl.org/np/RA2JlYTWhC4PuhqFITergBXYM0CdZ_H-uTJ751rOIntlU> ;

nt:wasCreatedFromProvenanceTemplate <http://purl.org/np/RAE1wniOy0yO39PlK9QkQ-wqbC3q-R2nXraP5huu8W39k> ;

nt:wasCreatedFromPubinfoTemplate <http://purl.org/np/RA2vCBXZf-icEcVRGhulJXugTGxpsV5yVr9yqCI1bQh4A> , <http://purl.org/np/RAA2MfqdBCzmz9yVWjKLXNbyfBNcwsMmOqcNUxkk1maIM> , <http://purl.org/np/RAOGu9Lh0BD4tbIRB9RG6RGRA_ObDh75NTbIqaWgxxs8M> , <http://purl.org/np/RAWv_eqe4tghg-OOg6NqRQODjC865Q0ZWkXTxqjSe59Y4> ;

nt:wasCreatedFromTemplate <http://purl.org/np/RAv68imZrEjfcp2rnEg1hzoBqEVc0cQMtp9_1Za0BxNM4> .

}

**References**

[1] Mietchen, D., Jakobi, J.W. & Richter, HP. Cortex reorganization of Xenopus laeviseggs in strong static magnetic fields. BioMag Res Tech 3, 2 (2005). doi: 10.1186/1477-044X-3-2.

[2] Bucur, C.I., Kuhn, T., Ceolin, D., Ossenbruggen, J. van. Expressing high-level scientific claims with formal semantics. In: Proceedings of the 11th Knowledge Capture Conference 2021. doi: 10.1145/3460210.3493561.
