# Supplementary material for: Nanopublication-based semantic publishing and reviewing: a field study with formalization papers
Source: PeerJ Comput Sci. 2023 Feb 21;9:e1159. doi: 10.7717/peerj-cs.1159 (PMC10280262; doi:10.7717/peerj-cs.1159)
Supplement: Supplemental Information 2 [file peerj-cs-09-1159-s002.zip › formalization_papers_supplemental-main/accepted_submissions/s3_Daniel_Mietchen.pdf]

Title: A formalization of one of the main claims of “Cortex reorganization of Xenopus laevis eggs in strong static magnetic fields” by Mietchen et al. 2005

Authors: Daniel Mietchen, ORCID: 0000-0001-9488-1870

Affiliations: Fraunhofer Institute for Biomedical Engineering (IBMT), Sulzbach, Germany.
E-mail: daniel.mietchen@ibmt.fraunhofer.de

Keywords: “dejellied fertilizable stage VI Xenopus laevis oocyte”, “strong static magnetic field”, “cell cortex”

Article Type: Formalization Paper

As RDF/nanopublication:
http://purl.org/np/RAXVRaFjWDlX5cZcVRXETaEIAx6QAyLK5JCrzDP-yDp9U

Editor: Cristina-Iulia Bucur, ORCID: 0000-0002-7114-6459

Review comments from:

- Michel Dumontier, ORCID: 0000-0003-4727-9435
- Tobias Kuhn, ORCID: 0000-0002-1267-0234
- Cristina-Iulia Bucur, ORCID: 0000-0002-7114-6459

Received: 2021-07-25

Accepted: 2021-11-17

Abstract:
Mietchen et al. claimed in previous work that strong static magnetic fields change the cell cortex in dejellied fertilizable stage VI Xenopus lavis oocytes. We present here a formalization of that claim, stating that all things of class “strong static magnetic field” that are in the context of a thing of class “dejellied fertilizable stage VI Xenopus laevis oocyte” generally have a relation of type “affects" to a thing of class “cell cortex” in the same context.

1. Introduction

Mietchen et al. [1] state that “A complex reorganization of cortical pigmentation was found in dejellied eggs as a function of the magnetic field and the field exposure time”. We present here a formalization of the main scientific claim from this quote by using a semantic template called the super-pattern [2].

2. Formalization

Our formalization looks as follows:

CONTEXT-CLASS ("in the context of all ..."):	dejellied fertilizable stage VI Xenopus laevis oocyte
SUBJECT-CLASS ("things of type ..."):	strong static magnetic field
QUALIFIER:	generally
RELATION-TYPE ("have a relation of type..."):	affects
OBJECT-CLASS ("to things of type..."):	cell cortex

In the context class, we use the class "dejellied fertilizable stage VI Xenopus laevis oocyte" (Q107644116) from Wikidata. In the subject class, we use the class "strong static magnetic field" (Q107644241) from Wikidata. In the object class, we use the class "cell cortex" (Q5058180) from Wikidata.

3. RDF Code

This is our formalization as a nanopublication in TriG format:

```
@prefix this: <http://purl.org/np/RAXVRaFjWDlX5cZcVRXETaEIAx6QAyLK5JCrzDP-yDp9U> .
@prefix sub: <http://purl.org/np/RAXVRaFjWDlX5cZcVRXETaEIAx6QAyLK5JCrzDP-yDp9U#> .
@prefix np: <http://www.nanopub.org/nschema#> .
@prefix dct: <http://purl.org/dc/terms/> .
@prefix nt: <https://w3id.org/np/o/ntemplate/> .
@prefix npx: <http://purl.org/nanopub/x/> .
@prefix xsd: <http://www.w3.org/2001/XMLSchema#> .
@prefix rdfs: <http://www.w3.org/2000/01/rdf-schema#> .
@prefix orcid: <https://orcid.org/> .
@prefix prov: <http://www.w3.org/ns/prov#> .
@prefix sp: <https://w3id.org/linkflows/superpattern/terms/> .

sub:Head {
  this: np:hasAssertion sub:assertion ;
    np:hasProvenance sub:provenance ;
    np:hasPublicationInfo sub:pubinfo ;
    a np:Nanopublication .
}
sub:assertion {
  sub:spi a sp:SuperPatternInstance ;
    rdfs:label "Strong static magnetic fields change the cell cortex in dejellied fertilizable stage VI Xenopus lavis oocytes." ;
    sp:hasContextClass <http://www.wikidata.org/entity/Q107644116> ;
    sp:hasSubjectClass <http://www.wikidata.org/entity/Q107644241> ;
    sp:hasQualifier sp:generallyQualifier ;
    sp:hasRelation sp:affects ;
    sp:hasObjectClass <http://www.wikidata.org/entity/Q5058180> .
}
sub:provenance {
  sub:activity a sp:FormalizationActivity ;
    prov:used sub:quote , <https://doi.org/10.1186/1477-044X-3-2> ;
    prov:wasAssociatedWith orcid:0000-0001-9488-1870 .
  sub:assertion prov:wasGeneratedBy sub:activity .
  sub:quote prov:value "A complex reorganization of cortical pigmentation was found in dejellied eggs as a function of the
magnetic field and the field exposure time." ;
    prov:wasQuotedFrom <https://doi.org/10.1186/1477-044X-3-2> .
}
sub:pubinfo {
  sub:sig npx:hasAlgorithm "RSA" ;
```

```
    npx:hasPublicKey
"MIGfMA0GCSqGSIb3DQEBAQUAA4GNADCBiQKBgQCJlM78d80R+gFMoQB1IG3f7AbqqGOCIv4HmZd1cx1KgEWMUUpPsojFNvx84fC/TltcJ8F8JafnbhDXW2HM2MhdK4yC
04ROEV1vIgSzjDicHfiqXvMqdPuMyQp4mmCEY7mUoeEW10mWZqjk+S9TnmiAQbFGcpExP8aosr2aTR7CSQIDAQAB" ;
    npx:hasSignature
"akW42kGSMe1sO8SU8VqcxrOSssOW3LLBQONsJBvsigKDV8AiBQ/MaR30ve20LhTgtrFQrwB1jA9ZhCy9zrYxyKVCRKvJzovPppGaTyHd8KCeAhsN0ZmSuu2XKUHqbiep
zahoPyxX0GdqCox9PS9D6ssFe8WoRHPVRk3Jzwd5k1I=" ;
    npx:hasSignatureTarget this: .
  this: dct:created "2021-12-17T11:18:24.918+01:00"^^xsd:dateTime ;
    dct:creator orcid:0000-0001-9488-1870 , orcid:0000-0002-7114-6459 ;
    npx:introduces sub:spi ;
    <https://w3id.org/linkflows/reviews/isUpdateOf> <http://purl.org/np/RA2JlYTWhC4PuhqFITergBXYM0CdZ_H-uTJ751rOIntlU> ;
    nt:wasCreatedFromProvenanceTemplate <http://purl.org/np/RAE1wniOy0yO39PlK9QkQ-wqbC3q-R2nXraP5huu8W39k> ;
    nt:wasCreatedFromPubinfoTemplate <http://purl.org/np/RA2vCBXZf-icEcVRGhulJXugTGxpsV5yVr9yqCI1bQh4A> ,
<http://purl.org/np/RAA2MfqdBCzmz9yVWjKLXNbyfBNcwsMmOqcNUxkk1maIM> ,
<http://purl.org/np/RAOGu9Lh0BD4tbIRB9RG6RGRA_ObDh75NTbIqaWgxxs8M> ,
<http://purl.org/np/RAWv_eqe4tghg-OOg6NqRQODjC865Q0ZWkXTxqjSe59Y4> ;
    nt:wasCreatedFromTemplate <http://purl.org/np/RAv68imZrEjfcp2rnEg1hzoBqEVc0cQMtp9_1Za0BxNM4> .
}
```


References

[1] Mietchen, D., Jakobi, J.W. & Richter, HP. Cortex reorganization of Xenopus laeviseggs in strong static magnetic fields. BioMag Res Tech 3, 2 (2005). doi: 10.1186/1477-044X-3-2.

[2] Bucur, C.I., Kuhn, T., Ceolin, D., Ossenbruggen, J. van. Expressing high-level scientific claims with formal semantics. In: Proceedings of the 11th Knowledge Capture Conference 2021. doi: 10.1145/3460210.3493561.
