# Supplementary material for: Nanopublication-based semantic publishing and reviewing: a field study with formalization papers
Source: PeerJ Comput Sci. 2023 Feb 21;9:e1159. doi: 10.7717/peerj-cs.1159 (PMC10280262; doi:10.7717/peerj-cs.1159)
Supplement: Supplemental Information 2 [file peerj-cs-09-1159-s002.zip › formalization_papers_supplemental-main/accepted_submissions/s4_Friederike_Ehrhart&Chris_Evelo.pdf]

Title: A formalization of one of the main claims of “Overlap of vitamin A and vitamin D target genes with CAKUT-related processes” by Ozisik et al. 2021

Authors: Friederike Ehrhart[1], ORCID: 0000-0002-7770-620X and Chris T. Evelo[2], ORCID: 0000-0002-5301-3142

Affiliations: [1]Department of Bioinformatics, NUTRIM/MHeNs, Maastricht University, The Netherlands. E-mail: friederike.ehrhart@maastrichtuniversity.nl ; [2] Department of Bioinformatics, NUTRIM, Maastricht University, The Netherlands and Maastricht Center for Systems Biology (MaCSBio), Maastricht University, The Netherlands. E-mail: chris.evelo@maastrichtuniversity.nl

Keywords: “genes associated with CAKUT”, “targets of Vitamin A”

Article Type: Formalization Paper

As RDF/nanopublication:
http://purl.org/np/RAyg4UgIVovBGia-hk4qEuRzOq14fcOIYAclC6YGQaVYU

Editor: Cristina-Iulia Bucur, ORCID: 0000-0002-7114-6459

Review comments from:

- Tobias Kuhn, ORCID: 0000-0002-1267-0234
- Cristina-Iulia Bucur, ORCID: 0000-0002-7114-6459

Received: 2021-09-24

Accepted: 2021-11-15

Abstract:
In a previous paper that we co-authored (Ozisik et al. 2021) we showed that in some cases Vitamin A and Vitamin D receptor binding sites are present in genes associated with Congenital Anomalies of the Kidney and Urinary Tract (CAKUT). From that finding we derived the statement that sometimes Vitamin A targets are the same as genes associated with CAKUT. We present here a formalization of that claim, stating that all things of class “genes associated with CAKUT” sometimes have a relation of type “is same as” to a thing of class “targets of vitamin A”.

1. Introduction

We present here a formalization of the main scientific claim from Ozisik et al. [1] by using a semantic template called the super-pattern [2].

2. Formalization

Our formalization looks as follows:

CONTEXT-CLASS ("in the context of all ..."):	[(universal context)]
SUBJECT-CLASS ("things of type ..."):	genes associated with CAKUT
QUALIFIER:	sometimes
RELATION-TYPE ("have a relation of type..."):	is same as
OBJECT-CLASS ("to things of type..."):	targets of vitamin A

In the context class we use the class "universal context" from SuperPattern ontology. In the subject class, we use the class "genes associated with CAKUT" (Q109406970) from Wikidata. In the object class we use the class "targets of vitamin A" (Q109406949) from Wikidata.

3. RDF Code

This is our formalization as a nanopublication in TriG format:

```
@prefix this: <http://purl.org/np/RAyg4UgIVovBGia-hk4qEuRzOq14fcOlYAclC6YGQaVYU> .
@prefix sub: <http://purl.org/np/RAyg4UgIVovBGia-hk4qEuRzOq14fcOlYAclC6YGQaVYU#> .
@prefix np: <http://www.nanopub.org/nschema#> .
@prefix dct: <http://purl.org/dc/terms/> .
@prefix nt: <https://w3id.org/np/o/ntemplate/> .
@prefix npx: <http://purl.org/nanopub/x/> .
@prefix xsd: <http://www.w3.org/2001/XMLSchema#> .
@prefix rdfs: <http://www.w3.org/2000/01/rdf-schema#> .
@prefix orcid: <https://orcid.org/> .
@prefix prov: <http://www.w3.org/ns/prov#> .
@prefix sp: <<https://w3id.org/linkflows/superpattern/terms/> .


sub:Head {
  this: np:hasAssertion sub:assertion ;
    np:hasProvenance sub:provenance ;
    np:hasPublicationInfo sub:pubinfo ;
    a np:Nanopublication .
}
sub:assertion {
  sub:spi a <https://w3id.org/linkflows/superpattern/terms/SuperPatternInstance> ;
    rdfs:label "Sometimes Vitamin A targets are the same as genes associated with CAKUT" ;
    sp:hasContextClass sp:UniversalContext ;
    sp:hasSubjectClass <https://www.wikidata.org/wiki/Q109406970> ;
    sp:hasQualifier sp:sometimesQualifier ;
    sp:hasRelation sp:isSameAs ;
    sp:hasObjectClass <https://www.wikidata.org/wiki/Q109406949> .
}
sub:provenance {
  sub:activity a sp:FormalizationActivity ;
    prov:used <https://doi.org/10.12688/f1000research.51018.1> ;
    prov:wasAssociatedWith orcid:0000-0002-5301-3142 , orcid:0000-0002-7770-620X .
  sub:assertion prov:wasGeneratedBy sub:activity .
}
sub:pubinfo {
  sub:sig npx:hasAlgorithm "RSA" ;
    npx:hasPublicKey
"MIGfMA0GCSqGSIb3DQEBAQUAA4GNADCBiQKBgQCCYtnHL3Zu9ExrWA28zHnRA9JKmj8V9awChjn+7oBn9p6wdLx61J5mD/lWK9H8NdxSk/fdoaEJAin2WLiWs6qBJdSZ
OLbfq7HSF/GVYoTkuvvjnf2rozS08mI+xwEXMsa7XVa+4bz8jauPyp/eEFbcIHEWtstiuQvjvoXKizK5HQIDAQAB" ;
    npx:hasSignature
"I3KzDEFUZEyP4oMCYA1MchEGcmMpEmFjZLwDF/TtxkXD1yoRj40BopL4n78X+5ldUVsO4ufY1J8JXe+TFgPXX/Z9mBLE+EbzhuX7pGRG9woM16C5fLTwrJoJL7rcUK6i
i7moFZ/LsChOKdtC0DrZuie+BecFjvt6xKi693gPih4=" ;
    npx:hasSignatureTarget this: .
  this: dct:created "2021-11-15T09:46:19.034+01:00"^^xsd:dateTime ;
    dct:creator orcid:0000-0002-7770-620X ;
```

```
    npx:introduces sub:spi ;
    npx:supersedes <http://purl.org/np/RAokVMmiZSbRh01diNeJLum4p13kUd-NZjGFuVtxVz4Bs> ;
    <https://w3id.org/linkflows/reviews/isUpdateOf> <http://purl.org/np/RAsdV8EQ1qn_lXOrgoG7mPaF1JXdFLzt2iYy4eMhMMuM4> ;
    nt:wasCreatedFromProvenanceTemplate <http://purl.org/np/RAE1wniOy0yO39PlK9QkQ-wqbC3q-R2nXraP5huu8W39k> ;
    nt:wasCreatedFromPubinfoTemplate <http://purl.org/np/RA2vCBXZf-icEcVRGhulJXugTGxpsV5yVr9yqCI1bQh4A> ,
<http://purl.org/np/RAA2MfqdBCzmz9yVWjKLXNbyfBNcwsMmOqcNUxkk1maIM> ,
<http://purl.org/np/RAjpBMlw3owYhJUBo3DtsuDlXsNAJ8cnGeWAutDVjuAuI> ;
    nt:wasCreatedFromTemplate <http://purl.org/np/RAv68imZrEjfcp2rnEg1hzoBqEVc0cQMtp9_1Za0BxNM4> .
}
```

Funding: FE and CEs work is supported by the funding from the European Union’s Horizon 2020 research and innovation programme under the EJP RD COFUND-EJP N° 825575.

References

[1] Ozisik O, Ehrhart F, Evelo CT et al. Overlap of vitamin A and vitamin D target genes with CAKUT-related processes [version 1; peer review: 2 approved with reservations]. F1000Research 2021, 395(10), doi: 10.12688/f1000research.51018.1.

[2] Bucur, C.I., Kuhn, T., Ceolin, D., Ossenbruggen, J. van. Expressing high-level scientific claims with formal semantics. In: Proceedings of the 11th Knowledge Capture Conference 2021. doi: 10.1145/3460210.3493561.
