# Supplementary material for: Nanopublication-based semantic publishing and reviewing: a field study with formalization papers
Source: PeerJ Comput Sci. 2023 Feb 21;9:e1159. doi: 10.7717/peerj-cs.1159 (PMC10280262; doi:10.7717/peerj-cs.1159)
Supplement: Supplemental Information 2 [file peerj-cs-09-1159-s002.zip › formalization_papers_supplemental-main/accepted_submissions/s5_George_Patrinos.docx]

**Title:** A formalization of one of the main claims of “Cost-effectiveness analysis of pharmacogenomics-guided clopidogrel treatment in Spanish patients undergoing percutaneous coronary intervention” by Fragoulakis et al. 2019

**Authors:** George P. Patrinos, ORCID: 0000-0002-0519-7776

**Affiliations:** University of Patras, Greece. E-mail: [gpatrinos@upatras.gr](mailto:gpatrinos@upatras.gr)

**Keywords:** “patient undergoing PCI”, “pharmacogenomics guided clopidogrel therapy”, “cost-effective treatment”

**Article Type:** Formalization Paper

**As RDF/nanopublication:** <http://purl.org/np/RAn15vsPJEVdJvjNKtBPo_oadtjeP9oc3Si-69FiJ4poQ>

**Editor:** Cristina-Iulia Bucur, ORCID: 0000-0002-7114-6459

**Review comments from:**

- Tobias Kuhn, ORCID: 0000-0002-1267-0234
- Davide Ceolin, ORCID: 0000-0002-3357-9130
- Margherita Martorana, ORCID: 0000-0001-8004-0464
- Cristina-Iulia Bucur, ORCID: 0000-0002-7114-6459

**Received:** 2021-07-05

**Accepted:** 2021-11-17

**Abstract:**

Fragoulakis et al. claimed in previous work that pharmacogenomics-guided clopidogrel strategy represents a cost-effective treatment for patients undergoing PCI. We present here a formalization of that claim, stating that all things of class “pharmacogenomics guided clopidogrel therapy” that are in the context of a thing of class “patient undergoing PCI” generally have a relation of type “enables” to a thing of class “cost-effective treatment” in the same context.

1. **Introduction**

Fragoulakis et al. [1] state that “Our data show that pharmacogenomics-guided clopidogrel treatment strategy may represent a cost-effective choice compared with non-pharmacogenomics-guided strategy for patients undergoing PCI.”. We present here a formalization of the main scientific claim from this quote by using a semantic template called the super-pattern [2].

1. **Formalization**

Our formalization looks as follows:

| CONTEXT-CLASS (“in the context of all ..."): | [patient undergoing PCI](http://purl.org/np/RA9pwySo43TIfbvPuhK4ZuisvMsDvZ6TeR5N6MNKft8Nw#patient_undergoing_PCI) |
| --- | --- |
| SUBJECT-CLASS (“things of type ..."): | [pharmacogenomics guided clopidogrel therapy](http://purl.org/np/RAOxICL4ULhzr5mxC9cyzStCBtpoETQGin6Vr-Ns7JNtA#pharmacogenomics_guided_clopidogrel_therapy) |
| QUALIFIER: | [generally](https://w3id.org/linkflows/superpattern/terms/generallyQualifier) |
| RELATION-TYPE (“have a relation of type...”): | [enables](https://w3id.org/linkflows/superpattern/terms/enables) |
| OBJECT-CLASS (“to things of type...”): | [cost-effective treatment](http://purl.org/np/RAlfRfPak2jsyyVy4knjOmxQSYtociP8Cc0O7gemMtqQY#cost-effective_treatment) |

In the context class we use a new minted class “patient undergoing PCI” that is a subclass of “patient” (Q181600) from Wikidata and is related to the class “percutaneous coronary intervention” (Q2008344) from Wikidata. In the subject class, we use a new minted class “pharmacogenomics guided clopidogrel therapy” that is a subclass of “treatment” (Q179661) from Wikidata and is related to the class “pharmacogenomics” (Q1152227) and “clopidogrel” (Q410237) from Wikidata. In the object class we minted a new class “cost-effective treatment” that is a subclass of “treatment” (Q179661) from Wikidata and is related to the class “cost-effectiveness analysis” (Q1754768) from Wikidata.

1. **RDF Code**

This is our formalization as a nanopublication in TriG format:

@prefix this: <http://purl.org/np/RAn15vsPJEVdJvjNKtBPo_oadtjeP9oc3Si-69FiJ4poQ> .

@prefix sub: <http://purl.org/np/RAn15vsPJEVdJvjNKtBPo_oadtjeP9oc3Si-69FiJ4poQ#> .

@prefix np: <http://www.nanopub.org/nschema#> .

@prefix dct: <http://purl.org/dc/terms/> .

@prefix nt: <https://w3id.org/np/o/ntemplate/> .

@prefix npx: <http://purl.org/nanopub/x/> .

@prefix xsd: <http://www.w3.org/2001/XMLSchema#> .

@prefix rdfs: <http://www.w3.org/2000/01/rdf-schema#> .

@prefix orcid: <https://orcid.org/> .

@prefix prov: <http://www.w3.org/ns/prov#> .

@prefix sp: <https://w3id.org/linkflows/superpattern/terms/> .

sub:Head {

this: np:hasAssertion sub:assertion ;

np:hasProvenance sub:provenance ;

np:hasPublicationInfo sub:pubinfo ;

a np:Nanopublication .

}

sub:assertion {

sub:spi a sp:SuperPatternInstance ;

rdfs:label "Pharmacogenomics-guided clopidogrel strategy represents a cost-effective treatment for patients undergoing PCI" ;

sp:hasContextClass <http://purl.org/np/RA9pwySo43TIfbvPuhK4ZuisvMsDvZ6TeR5N6MNKft8Nw#patient_undergoing_PCI> ;

sp:hasSubjectClass <http://purl.org/np/RAOxICL4ULhzr5mxC9cyzStCBtpoETQGin6Vr-Ns7JNtA#pharmacogenomics_guided_clopidogrel_therapy> ;

sp:hasQualifier sp:generallyQualifier ;

sp:hasRelation sp:enables ;

sp:hasObjectClass <http://purl.org/np/RAlfRfPak2jsyyVy4knjOmxQSYtociP8Cc0O7gemMtqQY#cost-effective_treatment> .

}

sub:provenance {

sub:activity a sp:FormalizationActivity ;

prov:used sub:quote , <https://pubmed.ncbi.nlm.nih.gov/30647444/> ;

prov:wasAssociatedWith orcid:0000-0002-0519-7776 .

sub:assertion prov:wasGeneratedBy sub:activity .

sub:quote prov:value "Our data show that pharmacogenomics-guided clopidogrel treatment strategy may represent a cost-effective choice compared with non-pharmacogenomics-guided strategy for patients undergoing PCI." ;

prov:wasQuotedFrom <https://pubmed.ncbi.nlm.nih.gov/30647444/> .

}

sub:pubinfo {

sub:sig npx:hasAlgorithm "RSA" ;

npx:hasPublicKey "MIGfMA0GCSqGSIb3DQEBAQUAA4GNADCBiQKBgQCJlM78d80R+gFMoQB1IG3f7AbqqGOCIv4HmZd1cx1KgEWMUUpPsojFNvx84fC/TltcJ8F8JafnbhDXW2HM2MhdK4yC04ROEV1vIgSzjDicHfiqXvMqdPuMyQp4mmCEY7mUoeEW10mWZqjk+S9TnmiAQbFGcpExP8aosr2aTR7CSQIDAQAB" ;

npx:hasSignature "cEQZgFxgTd99RYcmWsqM1LCUciLc1lXuy6mfjgKhn0BfsMcesqIKR+51xx6lfHD31tnz/bovIDh1YEPORGBe2PEvFMPvRRFd67B9s1awepEPll5tA7wmhkTOjPrEUXISQaQu93NbpbYMYSGnRx+Shv7jNxE+hPrxQw7fD6EgJr8=" ;

npx:hasSignatureTarget this: .

this: dct:created "2021-11-17T21:22:59.625+02:00"^^xsd:dateTime ;

dct:creator orcid:0000-0002-0519-7776 , orcid:0000-0002-7114-6459 ;

npx:introduces sub:spi ;

npx:supersedes <http://purl.org/np/RAB_yY8X4BUuS9HMBso8SvvPfBX31cb8cCv41SgfyMBew> ;

<https://w3id.org/linkflows/reviews/isUpdateOf> <http://purl.org/np/RAWCmrfeUUoh1tOl_7qFYoWgrYeonmX4FzAFboCSEoL-s> ;

nt:wasCreatedFromProvenanceTemplate <http://purl.org/np/RAE1wniOy0yO39PlK9QkQ-wqbC3q-R2nXraP5huu8W39k> ;

nt:wasCreatedFromPubinfoTemplate <http://purl.org/np/RA2vCBXZf-icEcVRGhulJXugTGxpsV5yVr9yqCI1bQh4A> , <http://purl.org/np/RAA2MfqdBCzmz9yVWjKLXNbyfBNcwsMmOqcNUxkk1maIM> , <http://purl.org/np/RAjpBMlw3owYhJUBo3DtsuDlXsNAJ8cnGeWAutDVjuAuI> ;

nt:wasCreatedFromTemplate <http://purl.org/np/RAv68imZrEjfcp2rnEg1hzoBqEVc0cQMtp9_1Za0BxNM4> .

}

The following nanopublications introduce the newly minted classes in TriG format.

This is the class definition of “patient undergoing PCI”:

@prefix this: <http://purl.org/np/RA9pwySo43TIfbvPuhK4ZuisvMsDvZ6TeR5N6MNKft8Nw> .

@prefix sub: <http://purl.org/np/RA9pwySo43TIfbvPuhK4ZuisvMsDvZ6TeR5N6MNKft8Nw#> .

@prefix np: <http://www.nanopub.org/nschema#> .

@prefix dct: <http://purl.org/dc/terms/> .

@prefix nt: <https://w3id.org/np/o/ntemplate/> .

@prefix npx: <http://purl.org/nanopub/x/> .

@prefix xsd: <http://www.w3.org/2001/XMLSchema#> .

@prefix rdfs: <http://www.w3.org/2000/01/rdf-schema#> .

@prefix orcid: <https://orcid.org/> .

@prefix prov: <http://www.w3.org/ns/prov#> .

@prefix skos: <http://www.w3.org/2004/02/skos/core#> .

sub:Head {

this: np:hasAssertion sub:assertion ;

np:hasProvenance sub:provenance ;

np:hasPublicationInfo sub:pubinfo ;

a np:Nanopublication .

}

sub:assertion {

sub:patient_undergoing_PCI a <http://www.w3.org/2002/07/owl#Class> ;

rdfs:label "patient undergoing PCI" ;

rdfs:subClassOf <http://www.wikidata.org/entity/Q181600> ;

skos:definition "patient undergoing percutaneous coronary intervention" ;

skos:relatedMatch <http://www.wikidata.org/entity/Q2008344> .

}

sub:provenance {

sub:assertion prov:wasAttributedTo orcid:0000-0002-0519-7776 .

}

sub:pubinfo {

sub:sig npx:hasAlgorithm "RSA" ;

npx:hasPublicKey "MIGfMA0GCSqGSIb3DQEBAQUAA4GNADCBiQKBgQCJlM78d80R+gFMoQB1IG3f7AbqqGOCIv4HmZd1cx1KgEWMUUpPsojFNvx84fC/TltcJ8F8JafnbhDXW2HM2MhdK4yC04ROEV1vIgSzjDicHfiqXvMqdPuMyQp4mmCEY7mUoeEW10mWZqjk+S9TnmiAQbFGcpExP8aosr2aTR7CSQIDAQAB" ;

npx:hasSignature "Iv6wAp5BYaY+1tBEBIcPls8rQjacsBZifWaNxBQkJaoYJs5/ejUrEiMXVwul4D+4baebbEm8rkT6vp336uDcencmlDiZzHZGL1ioFQTG1Bc//XZYWBkO/jcGRB3HNolScLz1Fo/dWaB94+qrW9MSTi6eVmIY9YqjH+3mGY6HJRk=" ;

npx:hasSignatureTarget this: .

this: dct:created "2021-11-04T10:24:46.053+02:00"^^xsd:dateTime ;

dct:creator orcid:0000-0002-0519-7776 , orcid:0000-0002-7114-6459 ;

npx:introduces sub:patient_undergoing_PCI ;

npx:supersedes <http://purl.org/np/RAuLZtfsHn3NGe0Md8lLNvYmBseEtW9v1rVUVfJCpJYUE> ;

<https://w3id.org/linkflows/reviews/isUpdateOf> <http://purl.org/np/RAuLZtfsHn3NGe0Md8lLNvYmBseEtW9v1rVUVfJCpJYUE> ;

nt:wasCreatedFromProvenanceTemplate <http://purl.org/np/RAi6zZAwhaJ23Hzg4lIjlPir6Take3ZQp-lS9skfBEwfQ> ;

nt:wasCreatedFromPubinfoTemplate <http://purl.org/np/RA2vCBXZf-icEcVRGhulJXugTGxpsV5yVr9yqCI1bQh4A> , <http://purl.org/np/RAA2MfqdBCzmz9yVWjKLXNbyfBNcwsMmOqcNUxkk1maIM> , <http://purl.org/np/RAOGu9Lh0BD4tbIRB9RG6RGRA_ObDh75NTbIqaWgxxs8M> , <http://purl.org/np/RAjpBMlw3owYhJUBo3DtsuDlXsNAJ8cnGeWAutDVjuAuI> ;

nt:wasCreatedFromTemplate <http://purl.org/np/RAdpgRpigXtt8iPV9uOPf3wIT3qzOI8Sg2Q72CNV8g-Yo> .

}

This is the class definition of “pharmacogenomics guided clopidogrel therapy”:

@prefix this: <http://purl.org/np/RAOxICL4ULhzr5mxC9cyzStCBtpoETQGin6Vr-Ns7JNtA> .

@prefix sub: <http://purl.org/np/RAOxICL4ULhzr5mxC9cyzStCBtpoETQGin6Vr-Ns7JNtA#> .

@prefix np: <http://www.nanopub.org/nschema#> .

@prefix dct: <http://purl.org/dc/terms/> .

@prefix nt: <https://w3id.org/np/o/ntemplate/> .

@prefix npx: <http://purl.org/nanopub/x/> .

@prefix xsd: <http://www.w3.org/2001/XMLSchema#> .

@prefix rdfs: <http://www.w3.org/2000/01/rdf-schema#> .

@prefix orcid: <https://orcid.org/> .

@prefix prov: <http://www.w3.org/ns/prov#> .

@prefix skos: <http://www.w3.org/2004/02/skos/core#> .

sub:Head {

this: np:hasAssertion sub:assertion ;

np:hasProvenance sub:provenance ;

np:hasPublicationInfo sub:pubinfo ;

a np:Nanopublication .

}

sub:assertion {

sub:pharmacogenomics_guided_clopidogrel_therapy a <http://www.w3.org/2002/07/owl#Class> ;

rdfs:label "pharmacogenomics guided clopidogrel therapy" ;

rdfs:subClassOf <http://www.wikidata.org/entity/Q179661> ;

skos:definition "A clopidogrel therapy assisted by pharmacogenomics." ;

skos:relatedMatch <http://www.wikidata.org/entity/Q1152227> , <http://www.wikidata.org/entity/Q410237> .

}

sub:provenance {

sub:assertion prov:wasAttributedTo orcid:0000-0002-0519-7776 .

}

sub:pubinfo {

sub:sig npx:hasAlgorithm "RSA" ;

npx:hasPublicKey "MIGfMA0GCSqGSIb3DQEBAQUAA4GNADCBiQKBgQCJlM78d80R+gFMoQB1IG3f7AbqqGOCIv4HmZd1cx1KgEWMUUpPsojFNvx84fC/TltcJ8F8JafnbhDXW2HM2MhdK4yC04ROEV1vIgSzjDicHfiqXvMqdPuMyQp4mmCEY7mUoeEW10mWZqjk+S9TnmiAQbFGcpExP8aosr2aTR7CSQIDAQAB" ;

npx:hasSignature "QNIcAE5wfsq1p8Z7J+KLQOwA1pbm5/VZFeRJe6JKkIB6TvXTdlO2OKOTDYMPZys15bU9yibaKOA/7YRQnaf6oTbgOmaT1mV5+zffRjl5SG+z+4A1R7HLIfr2G2x9Z4kz55rQL+AQbvYgkDXiVMSsbdJdraQ5t/SBCJcEHOh2xuQ=" ;

npx:hasSignatureTarget this: .

this: dct:created "2021-11-04T11:02:34.246+02:00"^^xsd:dateTime ;

dct:creator orcid:0000-0002-0519-7776 , orcid:0000-0002-7114-6459 ;

npx:introduces sub:pharmacogenomics_guided_clopidogrel_therapy ;

npx:supersedes <http://purl.org/np/RA4BJq9pLse6z1BRbZIOgARyf1zOY9Qw33ix3lsHcazOE> ;

<https://w3id.org/linkflows/reviews/isUpdateOf> <http://purl.org/np/RAvOAyEg-J8ynmZNrkCgg5XqGuqRo-dXcGFvgDLxtY6ck> ;

nt:wasCreatedFromProvenanceTemplate <http://purl.org/np/RAi6zZAwhaJ23Hzg4lIjlPir6Take3ZQp-lS9skfBEwfQ> ;

nt:wasCreatedFromPubinfoTemplate <http://purl.org/np/RA2vCBXZf-icEcVRGhulJXugTGxpsV5yVr9yqCI1bQh4A> , <http://purl.org/np/RAA2MfqdBCzmz9yVWjKLXNbyfBNcwsMmOqcNUxkk1maIM> , <http://purl.org/np/RAjpBMlw3owYhJUBo3DtsuDlXsNAJ8cnGeWAutDVjuAuI> ;

nt:wasCreatedFromTemplate <http://purl.org/np/RAdpgRpigXtt8iPV9uOPf3wIT3qzOI8Sg2Q72CNV8g-Yo> .

}

This is the class definition of “cost-effective treatment”:

@prefix this: <http://purl.org/np/RAlfRfPak2jsyyVy4knjOmxQSYtociP8Cc0O7gemMtqQY> .

@prefix sub: <http://purl.org/np/RAlfRfPak2jsyyVy4knjOmxQSYtociP8Cc0O7gemMtqQY#> .

@prefix np: <http://www.nanopub.org/nschema#> .

@prefix dct: <http://purl.org/dc/terms/> .

@prefix nt: <https://w3id.org/np/o/ntemplate/> .

@prefix npx: <http://purl.org/nanopub/x/> .

@prefix xsd: <http://www.w3.org/2001/XMLSchema#> .

@prefix rdfs: <http://www.w3.org/2000/01/rdf-schema#> .

@prefix orcid: <https://orcid.org/> .

@prefix prov: <http://www.w3.org/ns/prov#> .

@prefix skos: <http://www.w3.org/2004/02/skos/core#> .

sub:Head {

this: np:hasAssertion sub:assertion ;

np:hasProvenance sub:provenance ;

np:hasPublicationInfo sub:pubinfo ;

a np:Nanopublication .

}

sub:assertion {

sub:cost-effective_treatment a <http://www.w3.org/2002/07/owl#Class> ;

rdfs:label "cost-effective treatment" ;

rdfs:subClassOf <http://www.wikidata.org/entity/Q179661> ;

skos:definition "cost-effective treatment" ;

skos:relatedMatch <http://www.wikidata.org/entity/Q1754768> .

}

sub:provenance {

sub:assertion prov:wasAttributedTo orcid:0000-0002-0519-7776 .

}

sub:pubinfo {

sub:sig npx:hasAlgorithm "RSA" ;

npx:hasPublicKey "MIGfMA0GCSqGSIb3DQEBAQUAA4GNADCBiQKBgQCJlM78d80R+gFMoQB1IG3f7AbqqGOCIv4HmZd1cx1KgEWMUUpPsojFNvx84fC/TltcJ8F8JafnbhDXW2HM2MhdK4yC04ROEV1vIgSzjDicHfiqXvMqdPuMyQp4mmCEY7mUoeEW10mWZqjk+S9TnmiAQbFGcpExP8aosr2aTR7CSQIDAQAB" ;

npx:hasSignature "f+XJLoVxSGhXnuQYxbgd+cAl1jMEdNppqO11jalPaJlnUREFKwRB5A9mhsvEuV1TWGzHfzi1wEL8FiguHgg7YUTJlbA1pb8qA98/MFkWtXeYzIjwOgmuFUrSIBQF0cEVX5OV8D3nzwwa+Y2glBes11pIkSRgAH4b3KMy8+PbNBg=" ;

npx:hasSignatureTarget this: .

this: dct:created "2021-11-04T10:28:39.058+02:00"^^xsd:dateTime ;

dct:creator orcid:0000-0002-0519-7776 , orcid:0000-0002-7114-6459 ;

npx:introduces sub:cost-effective_treatment ;

npx:supersedes <http://purl.org/np/RAjD4-Q2kO_cIUE52rde2uWbV3RtuCR90geXW90il2YX4> ;

<https://w3id.org/linkflows/reviews/isUpdateOf> <http://purl.org/np/RAjD4-Q2kO_cIUE52rde2uWbV3RtuCR90geXW90il2YX4> ;

nt:wasCreatedFromProvenanceTemplate <http://purl.org/np/RAi6zZAwhaJ23Hzg4lIjlPir6Take3ZQp-lS9skfBEwfQ> ;

nt:wasCreatedFromPubinfoTemplate <http://purl.org/np/RA2vCBXZf-icEcVRGhulJXugTGxpsV5yVr9yqCI1bQh4A> , <http://purl.org/np/RAA2MfqdBCzmz9yVWjKLXNbyfBNcwsMmOqcNUxkk1maIM> , <http://purl.org/np/RAOGu9Lh0BD4tbIRB9RG6RGRA_ObDh75NTbIqaWgxxs8M> , <http://purl.org/np/RAjpBMlw3owYhJUBo3DtsuDlXsNAJ8cnGeWAutDVjuAuI> ;

nt:wasCreatedFromTemplate <http://purl.org/np/RAdpgRpigXtt8iPV9uOPf3wIT3qzOI8Sg2Q72CNV8g-Yo> .

}

**References**

[1] Fragoulakis V, Bartsakoulia M, Díaz-Villamarín X, Chalikiopoulou K, Kehagia K, Ramos JGS, Martínez-González LJ, Gkotsi M, Katrali E, Skoufas E, Vozikis A, John A, Ali BR, Wordsworth S, Dávila-Fajardo CL, Katsila T, Patrinos GP, Mitropoulou C. Cost-effectiveness analysis of pharmacogenomics-guided clopidogrel treatment in Spanish patients undergoing percutaneous coronary intervention. Pharmacogenomics J. 2019 Oct;19(5):438-445. doi: 10.1038/s41397-019-0069-1.

[2] Bucur, C.I., Kuhn, T., Ceolin, D., Ossenbruggen, J. van. Expressing high-level scientific claims with formal semantics. In: Proceedings of the 11th Knowledge Capture Conference 2021. doi: 10.1145/3460210.3493561.
