# Supplementary material for: Nanopublication-based semantic publishing and reviewing: a field study with formalization papers
Source: PeerJ Comput Sci. 2023 Feb 21;9:e1159. doi: 10.7717/peerj-cs.1159 (PMC10280262; doi:10.7717/peerj-cs.1159)
Supplement: Supplemental Information 2 [file peerj-cs-09-1159-s002.zip › formalization_papers_supplemental-main/accepted_submissions/s6_Margherita_Martorana.docx]

**Title:** A formalization of one of the main claims of “Sonic hedgehog signaling in astrocytes” by Hill et al. 2021

**Authors:** Margherita Martorana, ORCID: 0000-0001-8004-0464

**Affiliations:** Vrije Universiteit Amsterdam, The Netherlands. E-mail: [m.martorana@vu.nl](mailto:m.martorana@vu.nl)

**Keywords:** “human”, “smoothened signaling pathway”, “astrocyte development”

**Article Type:** Formalization Paper

**As RDF/nanopublication:** <http://purl.org/np/RA1FoHM9lwJ1XAV1eB871XcMAKfod73G_i4YtgoLpJVH0>

**Editor:** Cristina-Iulia Bucur, ORCID: 0000-0002-7114-6459

**Review comments from:**

- Tobias Kuhn, ORCID: 0000-0002-1267-0234
- Michel Dumontier, ORCID: 0000-0003-4727-9435
- Cristina-Iulia Bucur, ORCID: 0000-0002-7114-6459

**Received:** 2021-06-25

**Accepted:** 2021-11-12

**Abstract:**

Hill et al. claimed in previous work that sonic hedgehog signalling pathway is an essential regulator of astrocytes development. We present here a formalization of that claim, stating that all things of class “smoothened signaling pathway” that are in the context of a thing of class “human” mostly have a relation of type “affects” to a thing of class “astrocyte development” in the same context.

1. **Introduction**

Hill et al. [1] state that “Shh signaling and emerging data point to essential roles for this pleiotropic signaling pathway in regulating various functional properties of astrocytes.”. We present here a formalization of the main scientific claim from this quote by using a semantic template called the super-pattern [2].

1. **Formalization**

Our formalization looks as follows:

| CONTEXT-CLASS (“in the context of all ..."): | [human](http://www.wikidata.org/entity/Q5) |
| --- | --- |
| SUBJECT-CLASS (“things of type ..."): | [smoothened signaling pathway](http://purl.obolibrary.org/obo/GO_0007224) |
| QUALIFIER: | [mostly](https://w3id.org/linkflows/superpattern/terms/mostlyQualifier) |
| RELATION-TYPE (“have a relation of type...”): | [affects](https://w3id.org/linkflows/superpattern/terms/affects) |
| OBJECT-CLASS (“to things of type...”): | [astrocyte development](http://purl.obolibrary.org/obo/GO_0014002) |

In the context class we use the “human” (Q5) class from Wikipedia. In subject class, we use the “smoothened signaling pathway” (GO:0007224) from GeneOntology. In the object class we used the “astrocyte development” (GO:0014002) class from GeneOntology.

1. **RDF Code**

This is our formalization as a nanopublication in TriG format:

@prefix this: <http://purl.org/np/RA1FoHM9lwJ1XAV1eB871XcMAKfod73G_i4YtgoLpJVH0> .

@prefix sub: <http://purl.org/np/RA1FoHM9lwJ1XAV1eB871XcMAKfod73G_i4YtgoLpJVH0#> .

@prefix np: <http://www.nanopub.org/nschema#> .

@prefix dct: <http://purl.org/dc/terms/> .

@prefix nt: <https://w3id.org/np/o/ntemplate/> .

@prefix npx: <http://purl.org/nanopub/x/> .

@prefix xsd: <http://www.w3.org/2001/XMLSchema#> .

@prefix rdfs: <http://www.w3.org/2000/01/rdf-schema#> .

@prefix orcid: <https://orcid.org/> .

@prefix prov: <http://www.w3.org/ns/prov#> .

@prefix sp: <https://w3id.org/linkflows/superpattern/terms/> .

sub:Head {

this: np:hasAssertion sub:assertion ;

np:hasProvenance sub:provenance ;

np:hasPublicationInfo sub:pubinfo ;

a np:Nanopublication .

}

sub:assertion {

sub:spi a <https://w3id.org/linkflows/superpattern/terms/SuperPatternInstance> ;

rdfs:label "Sonic hedgehog signalling pathway is an essential regulator of astrocytes development." ;

sp:hasContextClass <http://www.wikidata.org/entity/Q5> ;

sp:hasSubjectClass <http://purl.obolibrary.org/obo/GO_0007224> ;

sp:hasQualifier sp:mostlyQualifier ;

sp:hasRelation sp:affects ;

sp:hasObjectClass <http://purl.obolibrary.org/obo/GO_0014002> .

}

sub:provenance {

sub:activity a sp:FormalizationActivity ;

prov:used sub:quote , <https://link.springer.com/article/10.1007%2Fs00018-020-03668-8> ;

prov:wasAssociatedWith orcid:0000-0001-8004-0464 .

sub:assertion prov:wasGeneratedBy sub:activity .

sub:quote prov:value "Shh signaling and emerging data point to essential roles for this pleiotropic signaling pathway in regulating various functional properties of astrocytes." ;

prov:wasQuotedFrom <https://link.springer.com/article/10.1007%2Fs00018-020-03668-8> .

}

sub:pubinfo {

sub:sig npx:hasAlgorithm "RSA" ;

npx:hasPublicKey "MIGfMA0GCSqGSIb3DQEBAQUAA4GNADCBiQKBgQDs0t7O15Wx/NFoleAZFCOuayiJlHtJ7daow/5JX9WuaUi0hjKn+wPdhgxDuxQvTPQIe8D6JE1LZnY2LXBSOzDcHKn+QWB6Zkn/ZisiG24V5C0kGpNji6Ab0gaAFZYl32VdS0qLPr34LLsEDzJRUoZHWxg0KoHw85F0EzlrPH+JpwIDAQAB" ;

npx:hasSignature "k7zk9oeQr6IarkWA3guYqppm8oIdPR8cWvcJWsi+iyQUXLG3s7BOD5oqAPzfTQ0BYwl91ZIIO5kXyJ4sob/m4lSJUc6AQ3XqNbgg5hIsL/F5EUo9XpL511ywLMYVKJ054/HrTvDw0oip/0Z4KKKmRPse7PeyE9b6fOMj/wz8jAo=" ;

npx:hasSignatureTarget this: .

this: dct:created "2021-10-20T12:00:18.181+02:00"^^xsd:dateTime ;

dct:creator orcid:0000-0001-8004-0464 ;

npx:introduces sub:spi ;

<https://w3id.org/linkflows/reviews/isUpdateOf> <http://purl.org/np/RAmfrSLt-WVQVNTrJi6IlNk3ZiQyYBds0NYGJpUEsPjfI> ;

nt:wasCreatedFromProvenanceTemplate <http://purl.org/np/RAB_oy10D3XUP-zYlqGz7Uj58AsUXhEKeGqmRFg5LSgDM> ;

nt:wasCreatedFromPubinfoTemplate <http://purl.org/np/RAA2MfqdBCzmz9yVWjKLXNbyfBNcwsMmOqcNUxkk1maIM> , <http://purl.org/np/RAOGu9Lh0BD4tbIRB9RG6RGRA_ObDh75NTbIqaWgxxs8M> ;

nt:wasCreatedFromTemplate <http://purl.org/np/RAv68imZrEjfcp2rnEg1hzoBqEVc0cQMtp9_1Za0BxNM4> .

}

**References**

[1] Hill, S.A., Fu, M. & Garcia, A.D.R. Sonic hedgehog signaling in astrocytes. Cell. Mol. Life Sci. 78, 1393–1403 (2021). Doi: 10.1007/s00018-020-03668-8.

[2] Bucur, C.I., Kuhn, T., Ceolin, D., Ossenbruggen, J. van. Expressing high-level scientific claims with formal semantics. In: Proceedings of the 11th Knowledge Capture Conference 2021. doi: 10.1145/3460210.3493561.
