# Supplementary material for: Nanopublication-based semantic publishing and reviewing: a field study with formalization papers
Source: PeerJ Comput Sci. 2023 Feb 21;9:e1159. doi: 10.7717/peerj-cs.1159 (PMC10280262; doi:10.7717/peerj-cs.1159)
Supplement: Supplemental Information 2 [file peerj-cs-09-1159-s002.zip › formalization_papers_supplemental-main/accepted_submissions/s6_Margherita_Martorana.pdf]

Title: A formalization of one of the main claims of "Sonic hedgehog signaling in astrocytes" by Hill et al. 2021

Authors: Margherita Martorana, ORCID: 0000-0001-8004-0464

Affiliations: Vrije Universiteit Amsterdam, The Netherlands. E-mail: m.martorana@vu.nl

Keywords: "human", "smoothened signaling pathway", "astrocyte development"

Article Type: Formalization Paper

As RDF/nanopublication:
http://purl.org/np/RA1FoHM9lwJ1XAV1eB871XcMAKfod73G_i4YtgoLpJVH0

Editor: Cristina-Iulia Bucur, ORCID: 0000-0002-7114-6459

Review comments from:

- Tobias Kuhn, ORCID: 0000-0002-1267-0234
- Michel Dumontier, ORCID: 0000-0003-4727-9435
- Cristina-Iulia Bucur, ORCID: 0000-0002-7114-6459

Received: 2021-06-25

Accepted: 2021-11-12

Abstract:
Hill et al. claimed in previous work that sonic hedgehog signalling pathway is an essential regulator of astrocytes development. We present here a formalization of that claim, stating that all things of class "smoothened signaling pathway" that are in the context of a thing of class "human" mostly have a relation of type "affects" to a thing of class "astrocyte development" in the same context.

1. Introduction

Hill et al. [1] state that "Shh signaling and emerging data point to essential roles for this pleiotropic signaling pathway in regulating various functional properties of astrocytes.". We present here a formalization of the main scientific claim from this quote by using a semantic template called the super-pattern [2].

2. Formalization

Our formalization looks as follows:

CONTEXT-CLASS (“in the context of all ..."):	human
SUBJECT-CLASS (“things of type ..."):	smoothened signaling pathway
QUALIFIER:	mostly
RELATION-TYPE (“have a relation of type...”):	affects
OBJECT-CLASS (“to things of type...”):	astrocyte development

In the context class we use the “human” (Q5) class from Wikipedia. In subject class, we use the “smoothened signaling pathway” (GO:0007224) from GeneOntology. In the object class we used the “astrocyte development” (GO:0014002) class from GeneOntology.

3. RDF Code

This is our formalization as a nanopublication in TriG format:

```
@prefix this: <http://purl.org/np/RA1FoHM9lwJ1XAV1eB871XcMAKfod73G_i4YtgoLpJVH0> .
@prefix sub: <http://purl.org/np/RA1FoHM9lwJ1XAV1eB871XcMAKfod73G_i4YtgoLpJVH0#> .
@prefix np: <http://www.nanopub.org/nschema#> .
@prefix dct: <http://purl.org/dc/terms/> .
@prefix nt: <https://w3id.org/np/o/ntemplate/> .
@prefix npx: <http://purl.org/nanopub/x/> .
@prefix xsd: <http://www.w3.org/2001/XMLSchema#> .
@prefix rdfs: <http://www.w3.org/2000/01/rdf-schema#> .
@prefix orcid: <https://orcid.org/> .
@prefix prov: <http://www.w3.org/ns/prov#> .
@prefix sp: <https://w3id.org/linkflows/superpattern/terms/> .


sub:Head {
  this: np:hasAssertion sub:assertion ;
    np:hasProvenance sub:provenance ;
    np:hasPublicationInfo sub:pubinfo ;
    a np:Nanopublication .
}
sub:assertion {
  sub:spi a <https://w3id.org/linkflows/superpattern/terms/SuperPatternInstance> ;
    rdfs:label "Sonic hedgehog signalling pathway is an essential regulator of astrocytes development." ;
    sp:hasContextClass <http://www.wikidata.org/entity/Q5> ;
    sp:hasSubjectClass <http://purl.obolibrary.org/obo/GO_0007224> ;
    sp:hasQualifier sp:mostlyQualifier ;
    sp:hasRelation sp:affects ;
    sp:hasObjectClass <http://purl.obolibrary.org/obo/GO_0014002> .
}
sub:provenance {
  sub:activity a sp:FormalizationActivity ;
    prov:used sub:quote , <https://link.springer.com/article/10.1007%2Fs00018-020-03668-8> ;
    prov:wasAssociatedWith orcid:0000-0001-8004-0464 .
  sub:assertion prov:wasGeneratedBy sub:activity .
  sub:quote prov:value "Shh signaling and emerging data point to essential roles for this pleiotropic signaling pathway in
regulating various functional properties of astrocytes." ;
    prov:wasQuotedFrom <https://link.springer.com/article/10.1007%2Fs00018-020-03668-8> .
}
sub:pubinfo {
  sub:sig npx:hasAlgorithm "RSA" ;
    npx:hasPublicKey
"MIGfMA0GCSqGSIb3DQEBAQUAA4GNADCBiQKBgQDs0t7O15Wx/NFoleAZFCOuayiJlHtJ7daow/5JX9WuaUi0hjKn+wPdhgxDuxQvTPQIe8D6JE1LZnY2LXBSOzDcHKn+
QWB6Zkn/ZisiG24V5C0kGpNji6Ab0gaAFZYl32VdS0qLPr34LLsEDzJRUoZHWxg0KoHw85F0EzlrPH+JpwIDAQAB" ;
    npx:hasSignature
"k7zk9oeQr6IarkWA3guYqppm8oIdPR8cWvcJWsi+iyQUXLG3s7BOD5oqAPzfTQ0BYwl91ZIIO5kXyJ4sob/m4lSJUc6AQ3XqNbgg5hIsL/F5EUo9XpL511ywLMYVKJ05
4/HrTvDw0oip/0Z4KKKmRPse7PeyE9b6fOMj/wz8jAo=" ;
    npx:hasSignatureTarget this: .
  this: dct:created "2021-10-20T12:00:18.181+02:00"^^xsd:dateTime ;
```

```
    dct:creator orcid:0000-0001-8004-0464 ;
    npx:introduces sub:spi ;
    <https://w3id.org/linkflows/reviews/isUpdateOf> <http://purl.org/np/RAmfrSLt-WVQVNTrJi6IlNk3ZiQyYBds0NYGJpUEsPjfI> ;
    nt:wasCreatedFromProvenanceTemplate <http://purl.org/np/RAB_oy10D3XUP-zYlqGz7Uj58AsUXhEKeGqmRFg5LSgDM> ;
    nt:wasCreatedFromPubinfoTemplate <http://purl.org/np/RAA2MfqdBCzmz9yVWjKLXNbyfBNcwsMmOqcNUxkk1maIM> ,
<http://purl.org/np/RAOGu9Lh0BD4tbIRB9RG6RGRA_ObDh75NTbIqaWgxxs8M> ;
    nt:wasCreatedFromTemplate <http://purl.org/np/RAv68imZrEjfcp2rnEg1hzoBqEVc0cQMtp9_1Za0BxNM4> .
}
```

References

[1] Hill, S.A., Fu, M. & Garcia, A.D.R. Sonic hedgehog signaling in astrocytes. Cell. Mol. Life Sci. 78, 1393–1403 (2021). Doi: 10.1007/s00018-020-03668-8.
[2] Bucur, C.I., Kuhn, T., Ceolin, D., Ossenbruggen, J. van. Expressing high-level scientific claims with formal semantics. In: Proceedings of the 11th Knowledge Capture Conference 2021. doi: 10.1145/3460210.3493561.
