# Supplementary material for: Nanopublication-based semantic publishing and reviewing: a field study with formalization papers
Source: PeerJ Comput Sci. 2023 Feb 21;9:e1159. doi: 10.7717/peerj-cs.1159 (PMC10280262; doi:10.7717/peerj-cs.1159)
Supplement: Supplemental Information 2 [file peerj-cs-09-1159-s002.zip › formalization_papers_supplemental-main/accepted_submissions/s7_Daniel_Mietchen&Lyubomir_Penev&Mariya_Dimitrova.docx]

**Title:** A formalization of one of the main claims of “Creative Commons licenses and the non-commercial condition: Implications for the re-use of biodiversity information” by Hagedorn et al. 2011

**Authors:** Daniel Mietchen^[1]^, ORCID: 0000-0001-9488-1870 and

Lyubomir Penev^[2]^, ORCID: 0000-0002-2186-5033 and

Mariya Dimitrova^[3]^, ORCID: 0000-0002-8083-6048

**Affiliations:** ^[1]^ Freie Universität Berlin, Germany. E-mail: [daniel.mietchen@googlemail.com](mailto:daniel.mietchen@googlemail.com) ; ^[2]^ Bulgarian Academy of Sciences, Bulgaria and Pensoft Publishers, Bulgaria. E-mail: [l.penev@pensoft.net](mailto:l.penev@pensoft.net) ; ^[3]^ Bulgarian Academy of Sciences, Bulgaria and Pensoft Publishers, Bulgaria. E-mail: [m.dimitrova@pensoft.net](mailto:m.dimitrova@pensoft.net)

**Keywords:** “biodiversity data”, “license with non-commercial clause”, “data reuse”

**Article Type:** Formalization Paper

**As RDF/nanopublication:** <http://purl.org/np/RABzhulhaPhOzo9MxWxl230N72-azdlpMNwu_HtDqsuUc>

**Editor:** Cristina-Iulia Bucur, ORCID: 0000-0002-7114-6459

**Review comments from:**

- Tobias Kuhn, ORCID: 0000-0002-1267-0234
- Ricardo Usbeck, ORCID: 0000-0002-0191-7211
- Cristina-Iulia Bucur, ORCID: 0000-0002-7114-6459

**Received:** 2021-07-07

**Accepted:** 2021-12-03

**Abstract:**

Hagedorn et al. claimed in previous work that when talking about biodiversity data, licenses with a non-commercial clause generally inhibit data reuse. We present here a formalization of that claim, stating that all things of class “license with non-commercial clause” that are in the context of a thing of class “biodiversity data” generally have a relation of type “inhibits” to a thing of class “data reuse” in the same context.

1. **Introduction**

Hagedorn et al. [1] state that “Unfortunately, in science and education, knowledge and data are often intermingled with copyrighted expressions of the same. Many publishers establish barriers to knowledge sharing by asserting copyright on non-copyrightable plain or formal expressions of that knowledge. In the area of biodiversity, often dealing with textually expressed data or data expressed in images, this is a major obstacle.”. We present here a formalization of the main scientific claim from this quote by using a semantic template called the super-pattern [2].

1. **Formalization**

Our formalization looks as follows:

| CONTEXT-CLASS (“in the context of all ..."): | [biodiversity data](https://www.wikidata.org/wiki/Q28946370) |
| --- | --- |
| SUBJECT-CLASS (“things of type ..."): | [license with non-commercial clause](http://purl.org/np/RA5Txa3acYP9_MUWEw7s7wenDTB1QXNMB7UehJW-2E-_8#license-with-non-commercial-clause) |
| QUALIFIER: | [generally](https://w3id.org/linkflows/superpattern/terms/generallyQualifier) |
| RELATION-TYPE (“have a relation of type...”): | [inhibits](https://w3id.org/linkflows/superpattern/terms/inhibits) |
| OBJECT-CLASS (“to things of type...”): | [data reuse](https://www.wikidata.org/wiki/Q58023280) |

In the context class we use the class “biodiversity data” (Q28946370) from Wikidata. In the subject class, we use a new minted class “license with non-commercial clause” that is a subclass of “license” (Q79719) from Wikidata and is related to the class “Creative Commons NonCommercial” (Q65071627) from Wikidata. In the object class we use the class “data reuse” (Q58023280) from Wikidata.

1. **RDF Code**

This is our formalization as a nanopublication in TriG format:

@prefix this: <http://purl.org/np/RABzhulhaPhOzo9MxWxl230N72-azdlpMNwu_HtDqsuUc> .

@prefix sub: <http://purl.org/np/RABzhulhaPhOzo9MxWxl230N72-azdlpMNwu_HtDqsuUc#> .

@prefix np: <http://www.nanopub.org/nschema#> .

@prefix dct: <http://purl.org/dc/terms/> .

@prefix nt: <https://w3id.org/np/o/ntemplate/> .

@prefix npx: <http://purl.org/nanopub/x/> .

@prefix xsd: <http://www.w3.org/2001/XMLSchema#> .

@prefix rdfs: <http://www.w3.org/2000/01/rdf-schema#> .

@prefix orcid: <https://orcid.org/> .

@prefix prov: <http://www.w3.org/ns/prov#> .

@prefix sp: <https://w3id.org/linkflows/superpattern/terms/> .

sub:Head {

this: np:hasAssertion sub:assertion ;

np:hasProvenance sub:provenance ;

np:hasPublicationInfo sub:pubinfo ;

a np:Nanopublication .

}

sub:assertion {

sub:spi a sp:SuperPatternInstance ;

rdfs:label "When talking about biodiversity data, licenses with a non-commercial clause generally inhibit data reuse." ;

sp:hasContextClass <http://www.wikidata.org/entity/Q28946370> ;

sp:hasSubjectClass <http://purl.org/np/RA5Txa3acYP9_MUWEw7s7wenDTB1QXNMB7UehJW-2E-_8#license-with-non-commercial-clause> ;

sp:hasQualifier> sp:generallyQualifier ;

sp:hasRelation sp:inhibits ;

sp:hasObjectClass <http://www.wikidata.org/entity/Q58023280> .

}

sub:provenance {

sub:activity a sp:FormalizationActivity ;

prov:used sub:quote , <https://doi.org/10.3897/zookeys.150.2189> ;

prov:wasAssociatedWith orcid:0000-0001-9488-1870 , orcid:0000-0002-2186-5033 , orcid:0000-0002-8083-6048 .

sub:assertion prov:wasGeneratedBy sub:activity .

sub:quote prov:value "Unfortunately, in science and education, knowledge and data are often intermingled with copyrighted expressions of the same. Many publishers establish barriers to knowledge sharing by asserting copyright on non-copyrightable plain or formal expressions of that knowledge. In the area of biodiversity, often dealing with textually expressed data or data expressed in images, this is a major obstacle." ;

prov:wasQuotedFrom <https://doi.org/10.3897/zookeys.150.2189> .

}

sub:pubinfo {

sub:sig npx:hasAlgorithm "RSA" ;

npx:hasPublicKey "MIGfMA0GCSqGSIb3DQEBAQUAA4GNADCBiQKBgQCwi1YMnpV5z/0oMXyFYDKS5cirQQWTMZ/jbglmVY1gl3YAgrkqsQUmgaxA/ho+VV8jsfREIL8PT2czbmCjGjMJoNaKVqJq1GoIUV9+Kj9yXxNySyDAOTDsIM0zdj1BEu5uNbtfzwQPGuhVpgLR05Uk/meCyYLPs3BxC5zRbtulmwIDAQAB" ;

npx:hasSignature "pqWZhyfzNdZhmUc6gaCbmq2GWJb4R/pWwk4rMvlOn35Nx9y44fsz9zGWDyEEVg0frXq6EOsd5Umyq5YY1BIHFJBjiOKUaVmtxU+MuyCuTCbLp3s1k+hYl7ocAl40F5SOMfcY+Dqxte1rNQM5rmVQJa9wJ+mmKejz7Cs+Bm9xsD8=" ;

npx:hasSignatureTarget this: .

this: dct:created "2021-11-30T21:54:51.835+02:00"^^xsd:dateTime ;

dct:creator orcid:0000-0002-8083-6048 ;

npx:introduces sub:spi ;

npx:supersedes <http://purl.org/np/RA2hde9hXKfi0juhaF7yWd1aKsyZknPYj2Od-rLlAKmSM> ;

<https://w3id.org/linkflows/reviews/isUpdateOf> <http://purl.org/np/RAjk3YoNydLhbuD4BV9FD21ecELazvZibHHM7IXtJd-4A> ;

nt:wasCreatedFromProvenanceTemplate <http://purl.org/np/RAE1wniOy0yO39PlK9QkQ-wqbC3q-R2nXraP5huu8W39k> ;

nt:wasCreatedFromPubinfoTemplate <http://purl.org/np/RA2vCBXZf-icEcVRGhulJXugTGxpsV5yVr9yqCI1bQh4A> , <http://purl.org/np/RAA2MfqdBCzmz9yVWjKLXNbyfBNcwsMmOqcNUxkk1maIM> , <http://purl.org/np/RAjpBMlw3owYhJUBo3DtsuDlXsNAJ8cnGeWAutDVjuAuI> ;

nt:wasCreatedFromTemplate <http://purl.org/np/RAv68imZrEjfcp2rnEg1hzoBqEVc0cQMtp9_1Za0BxNM4> .

}

The following nanopublications introduce the newly minted classes in TriG format.

This is the class definition of “license with non-commercial clause”:

@prefix this: <http://purl.org/np/RA5Txa3acYP9_MUWEw7s7wenDTB1QXNMB7UehJW-2E-_8> .

@prefix sub: <http://purl.org/np/RA5Txa3acYP9_MUWEw7s7wenDTB1QXNMB7UehJW-2E-_8#> .

@prefix np: <http://www.nanopub.org/nschema#> .

@prefix dct: <http://purl.org/dc/terms/> .

@prefix nt: <https://w3id.org/np/o/ntemplate/> .

@prefix npx: <http://purl.org/nanopub/x/> .

@prefix xsd: <http://www.w3.org/2001/XMLSchema#> .

@prefix rdfs: <http://www.w3.org/2000/01/rdf-schema#> .

@prefix orcid: <https://orcid.org/> .

@prefix prov: <http://www.w3.org/ns/prov#> .

@prefix skos: <http://www.w3.org/2004/02/skos/core#> .

sub:Head {

this: np:hasAssertion sub:assertion ;

np:hasProvenance sub:provenance ;

np:hasPublicationInfo sub:pubinfo ;

a np:Nanopublication .

}

sub:assertion {

sub:license-with-non-commercial-clause a <http://www.w3.org/2002/07/owl#Class> ;

rdfs:label "license with a non-commercial clause" ;

rdfs:subClassOf <http://www.wikidata.org/entity/Q79719> ;

skos:definition "a type of license which contain a clause which prohibits use for commercial purposes" ;

skos:relatedMatch <http://www.wikidata.org/entity/Q65071627> .

}

sub:provenance {

sub:assertion prov:wasAttributedTo orcid:0000-0002-8083-6048 .

}

sub:pubinfo {

sub:sig npx:hasAlgorithm "RSA" ;

npx:hasPublicKey "MIGfMA0GCSqGSIb3DQEBAQUAA4GNADCBiQKBgQCwi1YMnpV5z/0oMXyFYDKS5cirQQWTMZ/jbglmVY1gl3YAgrkqsQUmgaxA/ho+VV8jsfREIL8PT2czbmCjGjMJoNaKVqJq1GoIUV9+Kj9yXxNySyDAOTDsIM0zdj1BEu5uNbtfzwQPGuhVpgLR05Uk/meCyYLPs3BxC5zRbtulmwIDAQAB" ;

npx:hasSignature "lv9gGKSM989WhbvU+7dDcM+xIFW6jJnN0Lc91RN3tagMFc4utpd3/DBLQg8eDWPL+H9zfUa7SQ0grqCKggz86CyRJYctYHP/baTyhgemrPO3gyDTob5CqKpNm6hJ3xhC8ud3KH1LyzM+9UZW29OKeIe+zBx0rSs4PgS4z+cURsg=" ;

npx:hasSignatureTarget this: .

this: dct:created "2021-10-28T10:20:26.973+03:00"^^xsd:dateTime ;

dct:creator orcid:0000-0002-8083-6048 ;

npx:introduces sub:license-with-non-commercial-clause ;

npx:supersedes <http://purl.org/np/RAlLxbLmjwDt_cTFqr44HHD0xfGJJbhuYLJn0GeGGa3lI> ;

nt:wasCreatedFromProvenanceTemplate <http://purl.org/np/RANwQa4ICWS5SOjw7gp99nBpXBasapwtZF1fIM3H2gYTM> ;

nt:wasCreatedFromPubinfoTemplate <http://purl.org/np/RAA2MfqdBCzmz9yVWjKLXNbyfBNcwsMmOqcNUxkk1maIM> , <http://purl.org/np/RAjpBMlw3owYhJUBo3DtsuDlXsNAJ8cnGeWAutDVjuAuI> ;

nt:wasCreatedFromTemplate <http://purl.org/np/RAdpgRpigXtt8iPV9uOPf3wIT3qzOI8Sg2Q72CNV8g-Yo> .

}

**References**

[1] Hagedorn, G., Mietchen, D., Agosti, D., Penev, L., Berendsohn, W., Hobern, D. Creative Commons licenses and the non-commercial condition: Implications for the re-use of biodiversity information.In: ZooKeys 150 (2011): 127-149. doi: 10.3897/zookeys.150.2189.

[2] Bucur, C.I., Kuhn, T., Ceolin, D., Ossenbruggen, J. van. Expressing high-level scientific claims with formal semantics. In: Proceedings of the 11th Knowledge Capture Conference 2021. doi: 10.1145/3460210.3493561.
