# Supplementary material for: Nanopublication-based semantic publishing and reviewing: a field study with formalization papers
Source: PeerJ Comput Sci. 2023 Feb 21;9:e1159. doi: 10.7717/peerj-cs.1159 (PMC10280262; doi:10.7717/peerj-cs.1159)
Supplement: Supplemental Information 2 [file peerj-cs-09-1159-s002.zip › formalization_papers_supplemental-main/accepted_submissions/s7_Daniel_Mietchen&Lyubomir_Penev&Mariya_Dimitrova.pdf]

Title: A formalization of one of the main claims of "Creative Commons licenses and the non-commercial condition: Implications for the re-use of biodiversity information" by Hagedorn et al. 2011

Authors: Daniel Mietchen[1], ORCID: 0000-0001-9488-1870 and
Lyubomir Penev[2], ORCID: 0000-0002-2186-5033 and
Mariya Dimitrova[3], ORCID: 0000-0002-8083-6048

Affiliations: [1] Freie Universität Berlin, Germany. E-mail: daniel.mietchen@googlemail.com ; [2] Bulgarian Academy of Sciences, Bulgaria and Pensoft Publishers, Bulgaria. E-mail: l.penev@pensoft.net ; [3] Bulgarian Academy of Sciences, Bulgaria and Pensoft Publishers, Bulgaria. E-mail: m.dimitrova@pensoft.net

Keywords: "biodiversity data", "license with non-commercial clause", "data reuse"

Article Type: Formalization Paper

As RDF/nanopublication:
http://purl.org/np/RABzhulhaPhOzo9MxWxI230N72-azdlpMNwu_HtDqsuUc

Editor: Cristina-Iulia Bucur, ORCID: 0000-0002-7114-6459

Review comments from:
- Tobias Kuhn, ORCID: 0000-0002-1267-0234
- Ricardo Usbeck, ORCID: 0000-0002-0191-7211
- Cristina-Iulia Bucur, ORCID: 0000-0002-7114-6459

Received: 2021-07-07

Accepted: 2021-12-03

Abstract:
Hagedorn et al. claimed in previous work that when talking about biodiversity data, licenses with a non-commercial clause generally inhibit data reuse. We present here a formalization of that claim, stating that all things of class "license with non-commercial clause" that are in the context of a thing of class "biodiversity data" generally have a relation of type "inhibits" to a thing of class "data reuse" in the same context.

1. Introduction

Hagedorn et al. [1] state that "Unfortunately, in science and education, knowledge and data are often intermingled with copyrighted expressions of the same. Many publishers establish barriers to knowledge sharing by asserting copyright on non-copyrightable plain or formal expressions of that knowledge. In the area of biodiversity, often dealing with textually expressed data or data

expressed in images, this is a major obstacle.”. We present here a formalization of the main scientific claim from this quote by using a semantic template called the super-pattern [2].

2. Formalization

Our formalization looks as follows:

CONTEXT-CLASS (“in the context of all ..."):	biodiversity data
SUBJECT-CLASS (“things of type ..."):	license with non-commercial clause
QUALIFIER:	generally
RELATION-TYPE (“have a relation of type...”):	inhibits
OBJECT-CLASS (“to things of type...”):	data reuse

In the context class we use the class “biodiversity data” (Q28946370) from Wikidata. In the subject class, we use a new minted class “license with non-commercial clause” that is a subclass of “license” (Q79719) from Wikidata and is related to the class “Creative Commons NonCommercial” (Q65071627) from Wikidata. In the object class we use the class “data reuse” (Q58023280) from Wikidata.

3. RDF Code

This is our formalization as a nanopublication in TriG format:

```
@prefix this: <http://purl.org/np/RABzhulhaPhOzo9MxWxl230N72-azdlpMNwu_HtDqsuUc> .
@prefix sub: <http://purl.org/np/RABzhulhaPhOzo9MxWxl230N72-azdlpMNwu_HtDqsuUc#> .
@prefix np: <http://www.nanopub.org/nschema#> .
@prefix dct: <http://purl.org/dc/terms/> .
@prefix nt: <https://w3id.org/np/o/ntemplate/> .
@prefix npx: <http://purl.org/nanopub/x/> .
@prefix xsd: <http://www.w3.org/2001/XMLSchema#> .
@prefix rdfs: <http://www.w3.org/2000/01/rdf-schema#> .
@prefix orcid: <https://orcid.org/> .
@prefix prov: <http://www.w3.org/ns/prov#> .
@prefix sp: <https://w3id.org/linkflows/superpattern/terms/> .

sub:Head {
  this: np:hasAssertion sub:assertion ;
    np:hasProvenance sub:provenance ;
    np:hasPublicationInfo sub:pubinfo ;
    a np:Nanopublication .
}
sub:assertion {
  sub:spi a sp:SuperPatternInstance ;
    rdfs:label "When talking about biodiversity data, licenses with a non-commercial clause generally inhibit data reuse." ;
    sp:hasContextClass <http://www.wikidata.org/entity/Q28946370> ;
    sp:hasSubjectClass <http://purl.org/np/RA5Txa3acYP9_MUWEw7s7wenDTB1QXNMB7UehJW-2E-_8#license-with-non-commercial-clause> ;
    sp:hasQualifier> sp:generallyQualifier ;
    sp:hasRelation sp:inhibits ;
    sp:hasObjectClass <http://www.wikidata.org/entity/Q58023280> .
}
sub:provenance {
  sub:activity a sp:FormalizationActivity ;
    prov:used sub:quote , <https://doi.org/10.3897/zookeys.150.2189> ;
    prov:wasAssociatedWith orcid:0000-0001-9488-1870 , orcid:0000-0002-2186-5033 , orcid:0000-0002-8083-6048 .
  sub:assertion prov:wasGeneratedBy sub:activity .
```

```
  sub:quote prov:value "Unfortunately, in science and education, knowledge and data are often intermingled with copyrighted
expressions of the same. Many publishers establish barriers to knowledge sharing by asserting copyright on non-copyrightable
plain or formal expressions of that knowledge. In the area of biodiversity, often dealing with textually expressed data or data
expressed in images, this is a major obstacle." ;
    prov:wasQuotedFrom <https://doi.org/10.3897/zookeys.150.2189> .
}
sub:pubinfo {
  sub:sig npx:hasAlgorithm "RSA" ;
    npx:hasPublicKey
"MIGfMA0GCSqGSIb3DQEBAQUAA4GNADCBiQKBgQCwi1YMnpV5z/0oMXyFYDKS5cirQQWTMZ/jbglmVY1gl3YAgrkqsQUmgaxA/ho+VV8jsfREIL8PT2czbmCjGjMJoNaK
VqJq1GoIUV9+Kj9yXxNySyDAOTDsIM0zdj1BEu5uNbtfzwQPGuhVpgLR05Uk/meCyYLPs3BxC5zRbtulmwIDAQAB" ;
    npx:hasSignature
"pqWZhyfzNdZhmUc6gaCbmq2GWJb4R/pWwk4rMvlOn35Nx9y44fsz9zGWDyEEVg0frXq6EOsd5Umyq5YY1BIHFJBjiOKUaVmtxU+MuyCuTCbLp3s1k+hYl7ocAl40F5SO
MfcY+Dqxte1rNQM5rmVQJa9wJ+mmKejz7Cs+Bm9xsD8=" ;
    npx:hasSignatureTarget this: .
  this: dct:created "2021-11-30T21:54:51.835+02:00"^^xsd:dateTime ;
    dct:creator orcid:0000-0002-8083-6048 ;
    npx:introduces sub:spi ;
    npx:supersedes <http://purl.org/np/RA2hde9hXKfi0juhaF7yWd1aKsyZknPYj2Od-rLlAKmSM> ;
    <https://w3id.org/linkflows/reviews/isUpdateOf> <http://purl.org/np/RAjk3YoNydLhbuD4BV9FD21ecELazvZibHHM7IXtJd-4A> ;
    nt:wasCreatedFromProvenanceTemplate <http://purl.org/np/RAE1wniOy0yO39PlK9QkQ-wqbC3q-R2nXraP5huu8W39k> ;
    nt:wasCreatedFromPubinfoTemplate <http://purl.org/np/RA2vCBXZf-icEcVRGhulJXugTGxpsV5yVr9yqCI1bQh4A> ,
<http://purl.org/np/RAA2MfqdBCzmz9yVWjKLXNbyfBNcwsMmOqcNUxkk1maIM> ,
<http://purl.org/np/RAjpBMlw3owYhJUBo3DtsuDlXsNAJ8cnGeWAutDVjuAuI> ;
    nt:wasCreatedFromTemplate <http://purl.org/np/RAv68imZrEjfcp2rnEg1hzoBqEVc0cQMtp9_1Za0BxNM4> .
}
```

The following nanopublications introduce the newly minted classes in TriG format.

This is the class definition of “license with non-commercial clause”:

```
@prefix this: <http://purl.org/np/RA5Txa3acYP9_MUWEw7s7wenDTB1QXNMB7UehJW-2E-_8> .
@prefix sub: <http://purl.org/np/RA5Txa3acYP9_MUWEw7s7wenDTB1QXNMB7UehJW-2E-_8#> .
@prefix np: <http://www.nanopub.org/nschema#> .
@prefix dct: <http://purl.org/dc/terms/> .
@prefix nt: <https://w3id.org/np/o/ntemplate/> .
@prefix npx: <http://purl.org/nanopub/x/> .
@prefix xsd: <http://www.w3.org/2001/XMLSchema#> .
@prefix rdfs: <http://www.w3.org/2000/01/rdf-schema#> .
@prefix orcid: <https://orcid.org/> .
@prefix prov: <http://www.w3.org/ns/prov#> .
@prefix skos: <http://www.w3.org/2004/02/skos/core#> .

sub:Head {
  this: np:hasAssertion sub:assertion ;
    np:hasProvenance sub:provenance ;
    np:hasPublicationInfo sub:pubinfo ;
    a np:Nanopublication .
}
sub:assertion {
  sub:license-with-non-commercial-clause a <http://www.w3.org/2002/07/owl#Class> ;
    rdfs:label "license with a non-commercial clause" ;
    rdfs:subClassOf <http://www.wikidata.org/entity/Q79719> ;
    skos:definition "a type of license which contain a clause which prohibits use for commercial purposes" ;
    skos:relatedMatch <http://www.wikidata.org/entity/Q65071627> .
}
sub:provenance {
  sub:assertion prov:wasAttributedTo orcid:0000-0002-8083-6048 .
}
sub:pubinfo {
  sub:sig npx:hasAlgorithm "RSA" ;
    npx:hasPublicKey
"MIGfMA0GCSqGSIb3DQEBAQUAA4GNADCBiQKBgQCwi1YMnpV5z/0oMXyFYDKS5cirQQWTMZ/jbglmVY1gl3YAgrkqsQUmgaxA/ho+VV8jsfREIL8PT2czbmCjGjMJoNaK
VqJq1GoIUV9+Kj9yXxNySyDAOTDsIM0zdj1BEu5uNbtfzwQPGuhVpgLR05Uk/meCyYLPs3BxC5zRbtulmwIDAQAB" ;
    npx:hasSignature
"lv9gGKSM989WhbvU+7dDcM+xIFW6jJnN0Lc91RN3tagMFc4utpd3/DBLQg8eDWPL+H9zfUa7SQ0grqCKggz86CyRJYctYHP/baTyhgemrPO3gyDTob5CqKpNm6hJ3xhC
8ud3KH1LyzM+9UZW29OKeIe+zBx0rSs4PgS4z+cURsg=" ;
    npx:hasSignatureTarget this: .
  this: dct:created "2021-10-28T10:20:26.973+03:00"^^xsd:dateTime ;
    dct:creator orcid:0000-0002-8083-6048 ;
    npx:introduces sub:license-with-non-commercial-clause ;
    npx:supersedes <http://purl.org/np/RAlLxbLmjwDt_cTFqr44HHD0xfGJJbhuYLJn0GeGGa3lI> ;
    nt:wasCreatedFromProvenanceTemplate <http://purl.org/np/RANwQa4ICWS5SOjw7gp99nBpXBasapwtZF1fIM3H2gYTM> ;
    nt:wasCreatedFromPubinfoTemplate <http://purl.org/np/RAA2MfqdBCzmz9yVWjKLXNbyfBNcwsMmOqcNUxkk1maIM> ,
<http://purl.org/np/RAjpBMlw3owYhJUBo3DtsuDlXsNAJ8cnGeWAutDVjuAuI> ;
    nt:wasCreatedFromTemplate <http://purl.org/np/RAdpgRpigXtt8iPV9uOPf3wIT3qzOI8Sg2Q72CNV8g-Yo> .
}
```

References

[1] Hagedorn, G., Mietchen, D., Agosti, D., Penev, L., Berendsohn, W., Hobern, D. Creative Commons licenses and the non-commercial condition: Implications for the re-use of biodiversity information.In: ZooKeys 150 (2011): 127-149. doi: 10.3897/zookeys.150.2189.
[2] Bucur, C.I., Kuhn, T., Ceolin, D., Ossenbruggen, J. van. Expressing high-level scientific claims with formal semantics. In: Proceedings of the 11th Knowledge Capture Conference 2021. doi: 10.1145/3460210.3493561.
