# Supplementary material for: Nanopublication-based semantic publishing and reviewing: a field study with formalization papers
Source: PeerJ Comput Sci. 2023 Feb 21;9:e1159. doi: 10.7717/peerj-cs.1159 (PMC10280262; doi:10.7717/peerj-cs.1159)
Supplement: Supplemental Information 2 [file peerj-cs-09-1159-s002.zip › formalization_papers_supplemental-main/accepted_submissions/s8_Mariya_Dimitrova.docx]

**Title:** A formalization of one of the main claims of “OpenBiodiv: A Knowledge Graph for Literature-Extracted Linked Open Data in Biodiversity Science” by Penev et al. 2019

**Authors:** Mariya Dimitrova, ORCID: 0000-0002-8083-6048

**Affiliations:** Bulgarian Academy of Sciences, Bulgaria and Pensoft Publishers, Bulgaria. E-mail: [m.dimitrova@pensoft.net](mailto:m.dimitrova@pensoft.net)

**Keywords:** “release of OpenBiodiv knowledge graph”, “triple in OpenBiodiv knowledge graph”, “semantic triple extracted from biodiversity literature”

**Article Type:** Formalization Paper

**As RDF/nanopublication:** <http://purl.org/np/RAMgThXW6xx8QiPmW9VhVuxWCN2ZWe-pmxDcFfdx_A7z0>

**Editor:** Cristina-Iulia Bucur, ORCID: 0000-0002-7114-6459

**Review comments from:**

- Tobias Kuhn, ORCID: 0000-0002-1267-0234
- Ricardo Usbeck, ORCID: 0000-0002-0191-7211
- Cristina-Iulia Bucur, ORCID: 0000-0002-7114-6459

**Received:** 2021-07-07

**Accepted:** 2021-12-03

**Abstract:**

Penev et al. claimed in previous work that triples in the OpenBiodiv knowledge graph releases are generally semantic triples extracted from biodiversity literature. We present here a formalization of that claim, stating that all things of class “triple in OpenBiodiv knowledge graph” that are in the context of a thing of class “release of OpenBiodiv knowledge graph” generally have a relation of type “is same as” to a thing of class “semantic triple extracted from biodiversity literature” in the same context.

1. **Introduction**

Penev et al. [1] state that “OpenBiodiv encompasses data extracted from more than 5000 scholarly articles published by Pensoft and many more taxonomic treatments extracted by Plazi from journals of other publishers. The data from both sources are converted to Resource Description Framework (RDF) and integrated in a graph database using the OpenBiodiv-O ontology and an RDF version of the Global Biodiversity Information Facility (GBIF) taxonomic backbone.”. We present here a formalization of the main scientific claim from this quote by using a semantic template called the super-pattern [2].

1. **Formalization**

Our formalization looks as follows:

| CONTEXT-CLASS (“in the context of all ..."): | [release of OpenBiodiv knowledge graph](http://purl.org/np/RAlm6vh2zpFLg189qrDYPtppkL790Pqaw-q2KUhyfJtRY#release-of-openbiodiv-knowledge-graph) |
| --- | --- |
| SUBJECT-CLASS (“things of type ..."): | [triple in OpenBiodiv knowledge graph](http://purl.org/np/RAaEkIiJLmBJP5kK3JdYjseCRqwutYbdnI8Q3VbzrK9VA#triple-in-openbiodiv-knowledge-graph) |
| QUALIFIER: | [generally](https://w3id.org/linkflows/superpattern/terms/generallyQualifier) |
| RELATION-TYPE (“have a relation of type...”): | [is same as](https://w3id.org/linkflows/superpattern/terms/isSameAs) |
| OBJECT-CLASS (“to things of type...”): | [semantic triple extracted from biodiversity literature](http://purl.org/np/RAEpHUXRKtaLE3Z24sgIUdaxwTBsK2bjshyq9yF00145Y#semantic-triples-extracted-from-biodiversity-literature) |

In the context class we use a new minted class “release of OpenBiodiv knowledge graph” that is a subclass of “software release” (Q20631656) from Wikidata and has a related class a new minted class “[OpenBiodiv knowledge graph](http://purl.org/np/RAY2Gg01ffDVLprAakuMqpizAjksasRtTal_UsCf94LL8#openbiodiv-knowledge-graph)” that is a subclass of the “knowledge graph” (Q33002955) class from Wikidata. In the subject class, we use a new minted class “triple in OpenBiodiv knowledge graph” that is related to a new minted class “[OpenBiodiv knowledge graph](http://purl.org/np/RAY2Gg01ffDVLprAakuMqpizAjksasRtTal_UsCf94LL8#openbiodiv-knowledge-graph)” that is a subclass of the “knowledge graph” (Q33002955) class from Wikidata. In the object class we minted a new class “semantic triple extracted from biodiversity literature” that is related to a new minted class “[OpenBiodiv knowledge graph](http://purl.org/np/RAY2Gg01ffDVLprAakuMqpizAjksasRtTal_UsCf94LL8#openbiodiv-knowledge-graph)” that is a subclass of the “knowledge graph” (Q33002955) class from Wikidata.

1. **RDF Code**

This is our formalization as a nanopublication in TriG format:

@prefix this: <http://purl.org/np/RAMgThXW6xx8QiPmW9VhVuxWCN2ZWe-pmxDcFfdx_A7z0> .

@prefix sub: <http://purl.org/np/RAMgThXW6xx8QiPmW9VhVuxWCN2ZWe-pmxDcFfdx_A7z0#> .

@prefix np: <http://www.nanopub.org/nschema#> .

@prefix dct: <http://purl.org/dc/terms/> .

@prefix nt: <https://w3id.org/np/o/ntemplate/> .

@prefix npx: <http://purl.org/nanopub/x/> .

@prefix xsd: <http://www.w3.org/2001/XMLSchema#> .

@prefix rdfs: <http://www.w3.org/2000/01/rdf-schema#> .

@prefix orcid: <https://orcid.org/> .

@prefix prov: <http://www.w3.org/ns/prov#> .

@prefix sp: <https://w3id.org/linkflows/superpattern/terms/> .

sub:Head {

this: np:hasAssertion sub:assertion ;

np:hasProvenance sub:provenance ;

np:hasPublicationInfo sub:pubinfo ;

a np:Nanopublication .

}

sub:assertion {

sub:spi a sp:SuperPatternInstance ;

rdfs:label "Triples in the OpenBiodiv knowledge graph releases are generally semantic triples extracted from biodiversity literature." ;

sp:hasContextClass <http://purl.org/np/RAlm6vh2zpFLg189qrDYPtppkL790Pqaw-q2KUhyfJtRY#release-of-openbiodiv-knowledge-graph> ;

sp:hasSubjectClass <http://purl.org/np/RAaEkIiJLmBJP5kK3JdYjseCRqwutYbdnI8Q3VbzrK9VA#triple-in-openbiodiv-knowledge-graph> ;

sp:hasQualifier sp:generallyQualifier ;

sp:hasRelation sp:isSameAs ;

sp:hasObjectClass <http://purl.org/np/RAEpHUXRKtaLE3Z24sgIUdaxwTBsK2bjshyq9yF00145Y#semantic-triples-extracted-from-biodiversity-literature> .

}

sub:provenance {

sub:activity a sp:FormalizationActivity ;

prov:used sub:quote , <https://doi.org/10.3390/publications7020038> ;

prov:wasAssociatedWith orcid:0000-0002-8083-6048 .

sub:assertion prov:wasGeneratedBy sub:activity .

sub:quote prov:value "OpenBiodiv encompasses data extracted from more than 5000 scholarly articles published by Pensoft and many more taxonomic treatments extracted by Plazi from journals of other publishers. The data from both sources are converted to Resource Description Framework (RDF) and integrated in a graph database using the OpenBiodiv-O ontology and an RDF version of the Global Biodiversity Information Facility (GBIF) taxonomic backbone." ;

prov:wasQuotedFrom <https://doi.org/10.3390/publications7020038> .

}

sub:pubinfo {

sub:sig npx:hasAlgorithm "RSA" ;

npx:hasPublicKey "MIGfMA0GCSqGSIb3DQEBAQUAA4GNADCBiQKBgQCwi1YMnpV5z/0oMXyFYDKS5cirQQWTMZ/jbglmVY1gl3YAgrkqsQUmgaxA/ho+VV8jsfREIL8PT2czbmCjGjMJoNaKVqJq1GoIUV9+Kj9yXxNySyDAOTDsIM0zdj1BEu5uNbtfzwQPGuhVpgLR05Uk/meCyYLPs3BxC5zRbtulmwIDAQAB" ;

npx:hasSignature "ZbscFpNaJ3Wi/kWcKUWM3P5FbINnIPh3Z/BHMuCI95sAZjbD6XQD4AVUf7XUfFcMZtK8QKRE6S88zdLayXPklZC2x2GWcrh0j7Rk5UisHVcyZrukL0jH0D7oxXw2xi+W1FhX+6X8cgilEcums1MTLZ9bx3BdA1Goii6pR32gOLQ=" ;

npx:hasSignatureTarget this: .

this: dct:created "2021-11-30T22:13:32.063+02:00"^^xsd:dateTime ;

dct:creator orcid:0000-0002-8083-6048 ;

npx:introduces sub:spi ;

npx:supersedes <http://purl.org/np/RAN_chNfWR9hbEkfjwOfMpq1piwOgAj6q2sJ-qbDzaLOk> ;

<https://w3id.org/linkflows/reviews/isUpdateOf> <http://purl.org/np/RAWcrMY8mnsI26BfokKid75MO1c2uVenyW9tmxj0VhbnY> ;

nt:wasCreatedFromProvenanceTemplate <http://purl.org/np/RAE1wniOy0yO39PlK9QkQ-wqbC3q-R2nXraP5huu8W39k> ;

nt:wasCreatedFromPubinfoTemplate <http://purl.org/np/RA2vCBXZf-icEcVRGhulJXugTGxpsV5yVr9yqCI1bQh4A> , <http://purl.org/np/RAA2MfqdBCzmz9yVWjKLXNbyfBNcwsMmOqcNUxkk1maIM> , <http://purl.org/np/RAjpBMlw3owYhJUBo3DtsuDlXsNAJ8cnGeWAutDVjuAuI> ;

nt:wasCreatedFromTemplate <http://purl.org/np/RAv68imZrEjfcp2rnEg1hzoBqEVc0cQMtp9_1Za0BxNM4> .

}

The following nanopublications introduce the newly minted classes in TriG format.

This is the class definition of “release of OpenBiodiv knowledge graph”:

@prefix this: <http://purl.org/np/RAlm6vh2zpFLg189qrDYPtppkL790Pqaw-q2KUhyfJtRY> .

@prefix sub: <http://purl.org/np/RAlm6vh2zpFLg189qrDYPtppkL790Pqaw-q2KUhyfJtRY#> .

@prefix np: <http://www.nanopub.org/nschema#> .

@prefix dct: <http://purl.org/dc/terms/> .

@prefix nt: <https://w3id.org/np/o/ntemplate/> .

@prefix npx: <http://purl.org/nanopub/x/> .

@prefix xsd: <http://www.w3.org/2001/XMLSchema#> .

@prefix rdfs: <http://www.w3.org/2000/01/rdf-schema#> .

@prefix orcid: <https://orcid.org/> .

@prefix prov: <http://www.w3.org/ns/prov#> .

@prefix skos: <http://www.w3.org/2004/02/skos/core#> .

sub:Head {

this: np:hasAssertion sub:assertion ;

np:hasProvenance sub:provenance ;

np:hasPublicationInfo sub:pubinfo ;

a np:Nanopublication .

}

sub:assertion {

sub:release-of-openbiodiv-knowledge-graph a <http://www.w3.org/2002/07/owl#Class> ;

rdfs:label "A release of the OpenBiodiv knowledge graph" ;

rdfs:subClassOf <http://www.wikidata.org/entity/Q20631656> ;

skos:definition "A release of the OpenBiodiv knowledge graph stored in a GraphDB repository at http://graph.openbiodiv.net/" ;

skos:relatedMatch <http://purl.org/np/RAY2Gg01ffDVLprAakuMqpizAjksasRtTal_UsCf94LL8#openbiodiv-knowledge-graph> .

}

sub:provenance {

sub:assertion prov:wasAttributedTo orcid:0000-0002-8083-6048 .

}

sub:pubinfo {

sub:sig npx:hasAlgorithm "RSA" ;

npx:hasPublicKey "MIGfMA0GCSqGSIb3DQEBAQUAA4GNADCBiQKBgQCwi1YMnpV5z/0oMXyFYDKS5cirQQWTMZ/jbglmVY1gl3YAgrkqsQUmgaxA/ho+VV8jsfREIL8PT2czbmCjGjMJoNaKVqJq1GoIUV9+Kj9yXxNySyDAOTDsIM0zdj1BEu5uNbtfzwQPGuhVpgLR05Uk/meCyYLPs3BxC5zRbtulmwIDAQAB" ;

npx:hasSignature "FXDBQ2xJ6NWoC7ai62Yd02KL1I45X1XQxAN1skRMXtENVvO0ZdzjKOjU8TBOyw23h1prP/Ei/k+BT2JgOVHS+LyJXoW0ncHMg9a9AFrhB/BX+S6SMLVCEh0h8uzD7GbnBv6Vet2ajQ7nWCSb4ZkTJa0iByrFU/bpQMBZ4JKvkRs=" ;

npx:hasSignatureTarget this: .

this: dct:created "2021-11-30T22:05:24.772+02:00"^^xsd:dateTime ;

dct:creator orcid:0000-0002-8083-6048 ;

npx:introduces sub:release-of-openbiodiv-knowledge-graph ;

npx:supersedes <http://purl.org/np/RAySzPQKOPqboKwpMIeTtYhCwmLZTdlmKhiMM9W_fmZKw> ;

nt:wasCreatedFromProvenanceTemplate <http://purl.org/np/RANwQa4ICWS5SOjw7gp99nBpXBasapwtZF1fIM3H2gYTM> ;

nt:wasCreatedFromPubinfoTemplate <http://purl.org/np/RAA2MfqdBCzmz9yVWjKLXNbyfBNcwsMmOqcNUxkk1maIM> , <http://purl.org/np/RAjpBMlw3owYhJUBo3DtsuDlXsNAJ8cnGeWAutDVjuAuI> ;

nt:wasCreatedFromTemplate <http://purl.org/np/RAdpgRpigXtt8iPV9uOPf3wIT3qzOI8Sg2Q72CNV8g-Yo> .

}

This is the class definition of “triple in OpenBiodiv knowledge graph”:

@prefix this: <http://purl.org/np/RAaEkIiJLmBJP5kK3JdYjseCRqwutYbdnI8Q3VbzrK9VA> .

@prefix sub: <http://purl.org/np/RAaEkIiJLmBJP5kK3JdYjseCRqwutYbdnI8Q3VbzrK9VA#> .

@prefix np: <http://www.nanopub.org/nschema#> .

@prefix dct: <http://purl.org/dc/terms/> .

@prefix nt: <https://w3id.org/np/o/ntemplate/> .

@prefix npx: <http://purl.org/nanopub/x/> .

@prefix xsd: <http://www.w3.org/2001/XMLSchema#> .

@prefix rdfs: <http://www.w3.org/2000/01/rdf-schema#> .

@prefix orcid: <https://orcid.org/> .

@prefix prov: <http://www.w3.org/ns/prov#> .

@prefix skos: <http://www.w3.org/2004/02/skos/core#> .

sub:Head {

this: np:hasAssertion sub:assertion ;

np:hasProvenance sub:provenance ;

np:hasPublicationInfo sub:pubinfo ;

a np:Nanopublication .

}

sub:assertion {

sub:triple-in-openbiodiv-knowledge-graph a <http://www.w3.org/2002/07/owl#Class> ;

rdfs:label "Triple in OpenBiodiv knowledge graph" ;

skos:definition "A semantic triple in the OpenBiodiv knowledge graph" ;

skos:relatedMatch <http://purl.org/np/RAY2Gg01ffDVLprAakuMqpizAjksasRtTal_UsCf94LL8#openbiodiv-knowledge-graph> .

}

sub:provenance {

sub:assertion prov:wasAttributedTo orcid:0000-0002-8083-6048 .

}

sub:pubinfo {

sub:sig npx:hasAlgorithm "RSA" ;

npx:hasPublicKey "MIGfMA0GCSqGSIb3DQEBAQUAA4GNADCBiQKBgQCwi1YMnpV5z/0oMXyFYDKS5cirQQWTMZ/jbglmVY1gl3YAgrkqsQUmgaxA/ho+VV8jsfREIL8PT2czbmCjGjMJoNaKVqJq1GoIUV9+Kj9yXxNySyDAOTDsIM0zdj1BEu5uNbtfzwQPGuhVpgLR05Uk/meCyYLPs3BxC5zRbtulmwIDAQAB" ;

npx:hasSignature "dPpPA1yEeyfIBRllzfX4pmiOoCUzGAxPLEf8+1/1CWBAecvBCtqsS+rUAWUnHEqHytgrvWlxZhEmw7oC3ilPPIl3Pkb8NwiJ1BcRgLOPbe2R5+1ws75TDzmaZKjUoHoMbB4oBIfT78o4YcULeUOiZww19rVWvddCv/JlXc2nSk8=" ;

npx:hasSignatureTarget this: .

this: dct:created "2021-11-30T22:09:26.986+02:00"^^xsd:dateTime ;

dct:creator orcid:0000-0002-8083-6048 ;

npx:introduces sub:triple-in-openbiodiv-knowledge-graph ;

nt:wasCreatedFromProvenanceTemplate <http://purl.org/np/RANwQa4ICWS5SOjw7gp99nBpXBasapwtZF1fIM3H2gYTM> ;

nt:wasCreatedFromPubinfoTemplate <http://purl.org/np/RAA2MfqdBCzmz9yVWjKLXNbyfBNcwsMmOqcNUxkk1maIM> ;

nt:wasCreatedFromTemplate <http://purl.org/np/RAdpgRpigXtt8iPV9uOPf3wIT3qzOI8Sg2Q72CNV8g-Yo> .

}

This is the class definition of “semantic triple extracted from biodiversity literature”:

@prefix this: <http://purl.org/np/RAEpHUXRKtaLE3Z24sgIUdaxwTBsK2bjshyq9yF00145Y> .

@prefix sub: <http://purl.org/np/RAEpHUXRKtaLE3Z24sgIUdaxwTBsK2bjshyq9yF00145Y#> .

@prefix np: <http://www.nanopub.org/nschema#> .

@prefix dct: <http://purl.org/dc/terms/> .

@prefix nt: <https://w3id.org/np/o/ntemplate/> .

@prefix npx: <http://purl.org/nanopub/x/> .

@prefix xsd: <http://www.w3.org/2001/XMLSchema#> .

@prefix rdfs: <http://www.w3.org/2000/01/rdf-schema#> .

@prefix orcid: <https://orcid.org/> .

@prefix prov: <http://www.w3.org/ns/prov#> .

@prefix skos: <http://www.w3.org/2004/02/skos/core#> .

sub:Head {

this: np:hasAssertion sub:assertion ;

np:hasProvenance sub:provenance ;

np:hasPublicationInfo sub:pubinfo ;

a np:Nanopublication .

}

sub:assertion {

sub:semantic-triples-extracted-from-biodiversity-literature a <http://www.w3.org/2002/07/owl#Class> ;

rdfs:label "semantic triple extracted from biodiversity literature" ;

rdfs:subClassOf <http://www.wikidata.org/entity/Q3539534> ;

skos:definition "semantic triple generated after information was extracted from biodiversity literature" ;

skos:relatedMatch <http://purl.org/np/RAMZHs5RfS70gZEtxxJjQtvsXZ03BBINhDPowhiqQ5WnU#semantic-triples-generated-from-XML-files> .

}

sub:provenance {

sub:assertion prov:wasAttributedTo orcid:0000-0002-8083-6048 .

}

sub:pubinfo {

sub:sig npx:hasAlgorithm "RSA" ;

npx:hasPublicKey "MIGfMA0GCSqGSIb3DQEBAQUAA4GNADCBiQKBgQCwi1YMnpV5z/0oMXyFYDKS5cirQQWTMZ/jbglmVY1gl3YAgrkqsQUmgaxA/ho+VV8jsfREIL8PT2czbmCjGjMJoNaKVqJq1GoIUV9+Kj9yXxNySyDAOTDsIM0zdj1BEu5uNbtfzwQPGuhVpgLR05Uk/meCyYLPs3BxC5zRbtulmwIDAQAB" ;

npx:hasSignature "MAKvDuDjhzerLDcmY46TxIDqqtyyUtkGAfkKhR107rtMwn7BwyIRLijiyIGjrjzQDeOkMQhPw0N8TlDXuMfp1N6C0QuSY6nbWnvMDM6tFVgxD3n7AHjPutj5N9RumjNlPzYffkzGyIA9BryrgzjAc6dEBn3VTJI1phJi7toCGjk=" ;

npx:hasSignatureTarget this: .

this: dct:created "2021-07-07T10:58:43.199+03:00"^^xsd:dateTime ;

dct:creator orcid:0000-0002-8083-6048 ;

npx:introduces sub:semantic-triples-extracted-from-biodiversity-literature ;

nt:wasCreatedFromProvenanceTemplate <http://purl.org/np/RANwQa4ICWS5SOjw7gp99nBpXBasapwtZF1fIM3H2gYTM> ;

nt:wasCreatedFromPubinfoTemplate <http://purl.org/np/RAA2MfqdBCzmz9yVWjKLXNbyfBNcwsMmOqcNUxkk1maIM> ;

nt:wasCreatedFromTemplate <http://purl.org/np/RAdpgRpigXtt8iPV9uOPf3wIT3qzOI8Sg2Q72CNV8g-Yo> .

}

This is the class definition of “OpenBiodiv knowledge graph”:

@prefix this: <http://purl.org/np/RAY2Gg01ffDVLprAakuMqpizAjksasRtTal_UsCf94LL8> .

@prefix sub: <http://purl.org/np/RAY2Gg01ffDVLprAakuMqpizAjksasRtTal_UsCf94LL8#> .

@prefix np: <http://www.nanopub.org/nschema#> .

@prefix dct: <http://purl.org/dc/terms/> .

@prefix nt: <https://w3id.org/np/o/ntemplate/> .

@prefix npx: <http://purl.org/nanopub/x/> .

@prefix xsd: <http://www.w3.org/2001/XMLSchema#> .

@prefix rdfs: <http://www.w3.org/2000/01/rdf-schema#> .

@prefix orcid: <https://orcid.org/> .

@prefix prov: <http://www.w3.org/ns/prov#> .

@prefx skos: <http://www.w3.org/2004/02/skos/core#> .

sub:Head {

this: np:hasAssertion sub:assertion ;

np:hasProvenance sub:provenance ;

np:hasPublicationInfo sub:pubinfo ;

a np:Nanopublication .

}

sub:assertion {

sub:openbiodiv-knowledge-graph a <http://www.w3.org/2002/07/owl#Class> ;

rdfs:label "OpenBiodiv knowledge graph" ;

rdfs:subClassOf <http://www.wikidata.org/entity/Q33002955> ;

skos:definition "a knowledge graph containing statements extracted from biodiversity literature" .

}

sub:provenance {

sub:assertion prov:wasAttributedTo orcid:0000-0002-8083-6048 .

}

sub:pubinfo {

sub:sig npx:hasAlgorithm "RSA" ;

npx:hasPublicKey "MIGfMA0GCSqGSIb3DQEBAQUAA4GNADCBiQKBgQCwi1YMnpV5z/0oMXyFYDKS5cirQQWTMZ/jbglmVY1gl3YAgrkqsQUmgaxA/ho+VV8jsfREIL8PT2czbmCjGjMJoNaKVqJq1GoIUV9+Kj9yXxNySyDAOTDsIM0zdj1BEu5uNbtfzwQPGuhVpgLR05Uk/meCyYLPs3BxC5zRbtulmwIDAQAB" ;

npx:hasSignature "WDSCKwvQnBIPTel8btfovXycebML+Pg+GVVREG15t1f5BTDrldaJGO8FN3K7rsCHp3Sel25pJyV3aySvg/lcEUFMHi46Xtxtyu1isZF7FQ0BVHM7zx+nacgxm4UW8lQMG0e9l9OUypkyQtVHphyXzgcE8L6qK+PonrSFp5twfy0=" ;

npx:hasSignatureTarget this: .

this: dct:created "2021-07-07T09:30:53.628+03:00"^^xsd:dateTime ;

dct:creator orcid:0000-0002-8083-6048 ;

npx:introduces sub:openbiodiv-knowledge-graph ;

nt:wasCreatedFromProvenanceTemplate <http://purl.org/np/RANwQa4ICWS5SOjw7gp99nBpXBasapwtZF1fIM3H2gYTM> ;

nt:wasCreatedFromPubinfoTemplate <http://purl.org/np/RAA2MfqdBCzmz9yVWjKLXNbyfBNcwsMmOqcNUxkk1maIM> ;

nt:wasCreatedFromTemplate <http://purl.org/np/RAdpgRpigXtt8iPV9uOPf3wIT3qzOI8Sg2Q72CNV8g-Yo> .

}

**Funding:** This research received funding from the European Union’s Horizon 2020 research and innovation programme under the Marie Sklodowska-Curie grant agreement IGNITE (No 764840).

**References**

[1] Penev, L.; Dimitrova, M.; Senderov, V.; Zhelezov, G.; Georgiev, T.; Stoev, P.; Simov, K. OpenBiodiv: A Knowledge Graph for Literature-Extracted Linked Open Data in Biodiversity Science. Publications 2019, 7, 38. doi: 10.3390/publications7020038.

[2] Bucur, C.I., Kuhn, T., Ceolin, D., Ossenbruggen, J. van. Expressing high-level scientific claims with formal semantics. In: Proceedings of the 11th Knowledge Capture Conference 2021. doi: 10.1145/3460210.3493561.
