# Supplementary material for: Nanopublication-based semantic publishing and reviewing: a field study with formalization papers
Source: PeerJ Comput Sci. 2023 Feb 21;9:e1159. doi: 10.7717/peerj-cs.1159 (PMC10280262; doi:10.7717/peerj-cs.1159)
Supplement: Supplemental Information 2 [file peerj-cs-09-1159-s002.zip › formalization_papers_supplemental-main/accepted_submissions/s9_Matthew_Brauer.pdf]

**Title:** A formalization of one of the main claims of “TDP-43 represses cryptic exon inclusion in FTD/ALS gene UNC13A” by Rosa Ma et al. 2021

**Authors:** Matthew Brauer, ORCID: 0000-0003-2310-3013

**Affiliations:** Maze Therapeutics, USA. E-mail: mbrauer@mazetx.com

**Keywords:** “UNC13A”, “TAR DNA binding protein”, “inclusion of cryptic exon”

**Article Type:** Formalization Paper

**As RDF/nanopublication:**
http://purl.org/np/RAXkuXJ4IK10Ai9F39_tOFDy6ewi7znau6OQhUEXP4nPc

**Editor:** Cristina-Iulia Bucur, ORCID: 0000-0002-7114-6459

**Review comments from:**
- Tobias Kuhn, ORCID: 0000-0002-1267-0234
- Cristina-Iulia Bucur, ORCID: 0000-0002-7114-6459

**Received:** 2021-05-24

**Accepted:** 2021-11-17

**Abstract:**
Rosa Ma et al. claimed in previous work that the protein TDP-43 represses cryptic exon inclusion in the gene UNC13A.. We present here a formalization of that claim, stating that all things of class “TAR DNA binding protein” that are in the context of a thing of class “UNC13A” generally have a relation of type “inhibits” to a thing of class “inclusion of cryptic exon” in the same context.

## 1. Introduction

Rosa Ma et al. [1] state that “Here we show that TDP-43 represses a cryptic exon splicing event in UNC13A.”. We present here a formalization of the main scientific claim from this quote by using a semantic template called the super-pattern [2].

## 2. Formalization

Our formalization looks as follows:

CONTEXT-CLASS (“in the context of all ..."): UNC13A
SUBJECT-CLASS (“things of type ..."): TAR DNA binding protein

| | |
|---|---|
| QUALIFIER: | generally |
| RELATION-TYPE (“have a relation of type...”): | inhibits |
| OBJECT-CLASS (“to things of type...”): | inclusion of cryptic exon |

In the context class we use the “UNC13A” (Q18036664) class from Wikipedia. In subject class, we use the ”TAR DNA binding protein” (Q21133247) from Wikidata. In the object class we used the “inclusion of cryptic exon” (VariO_0504) class from OBO ontology.

### 3. RDF Code

This is our formalization as a nanopublication in TriG format:

```
@prefix this: <http://purl.org/np/RAXkuXJ4IK10Ai9F39_tOFDy6ewi7znau6OQhUEXP4nPc> .
@prefix sub: <http://purl.org/np/RAXkuXJ4IK10Ai9F39_tOFDy6ewi7znau6OQhUEXP4nPc#> .
@prefix np: <http://www.nanopub.org/nschema#> .
@prefix dct: <http://purl.org/dc/terms/> .
@prefix nt: <https://w3id.org/np/o/ntemplate/> .
@prefix npx: <http://purl.org/nanopub/x/> .
@prefix xsd: <http://www.w3.org/2001/XMLSchema#> .
@prefix rdfs: <http://www.w3.org/2000/01/rdf-schema#> .
@prefix orcid: <https://orcid.org/> .
@prefix prov: <http://www.w3.org/ns/prov#> .
@prefix sp: <https://w3id.org/linkflows/superpattern/terms/> .


sub:Head {
  this: np:hasAssertion sub:assertion ;
    np:hasProvenance sub:provenance ;
    np:hasPublicationInfo sub:pubinfo ;
    a np:Nanopublication .
}
sub:assertion {
  sub:spi a <https://w3id.org/linkflows/superpattern/terms/SuperPatternInstance> ;
    rdfs:label "The protein TDP-43 represses cryptic exon inclusion in the gene UNC13A." ;
    sp:hasContextClass <http://www.wikidata.org/entity/Q18036664> ;
    sp:hasSubjectClass <http://www.wikidata.org/entity/Q21133247> ;
    sp:hasQualifier sp:generallyQualifier ;
    sp:hasRelation sp:inhibits ;
    sp:hasObjectClass <http://purl.obolibrary.org/obo/VariO_0504> .
}
sub:provenance {
  sub:activity a sp:FormalizationActivity ;
    prov:used sub:quote , <https://www.biorxiv.org/content/10.1101/2021.04.02.438213v1> ;
    prov:wasAssociatedWith orcid:0000-0003-2310-3013 .
  sub:assertion prov:wasGeneratedBy sub:activity .
  sub:quote prov:value "Here we show that TDP-43 represses a cryptic exon splicing event in UNC13A." ;
    prov:wasQuotedFrom <https://www.biorxiv.org/content/10.1101/2021.04.02.438213v1> .
}
sub:pubinfo {
  sub:sig npx:hasAlgorithm "RSA" ;
    npx:hasPublicKey
"MIGfMA0GCSqGSIb3DQEBAQUAA4GNADCBiQKBgQCKr8mDKqduV4sU41GiCvB3R8Hrv3cdc+FxyCD0iONSayErC8oLDfaMBKZSLMkPBapyXeAjWbYmhuey7COxiakpTqSG
Kzy8AnMKNZ7tgd3KATKBPLQiawisIxx0BFpxw50yA2spZhv2bEpdni9wUOGa1MG+0sKC6bo2DnjxxxeA/wIDAQAB" ;
    npx:hasSignature
"PBzoLld6dCDC9ZgL1KNN0L2bEiH1Z7MpIHnpGlhKKV7YqsrlFL9pS6z6mtMTrdfZwSqupg1o2pwWluWqIzindCa8rTEVzcQAOrEvFP/Xg0yef/f7lDeVrvyVZDxhhgWU
LwH8hesWGqhWgY0cLlmGsXtS4ft5GVU7o60AO5HMCAM=" ;
    npx:hasSignatureTarget this: .
  this: dct:created "2021-10-26T13:03:28.286Z"^^xsd:dateTime ;
    dct:creator orcid:0000-0003-2310-3013 ;
    npx:introduces sub:spi ;
    <https://w3id.org/linkflows/reviews/isUpdateOf> <http://purl.org/np/RAIWvdnjqmOl2VP_eR2oFBxk97A_dw__FeusMzuRE9SIY> ;
    nt:wasCreatedFromProvenanceTemplate <http://purl.org/np/RAE1wniOy0yO39PlK9QkQ-wqbC3q-R2nXraP5huu8W39k> ;
    nt:wasCreatedFromPubinfoTemplate <http://purl.org/np/RAA2MfqdBCzmz9yVWjKLXNbyfBNcwsMmOqcNUxkk1maIM> ,
<http://purl.org/np/RAOGu9Lh0BD4tbIRB9RG6RGRA_ObDh75NTbIqaWgxxs8M> ;
    nt:wasCreatedFromTemplate <http://purl.org/np/RAv68imZrEjfcp2rnEg1hzoBqEVc0cQMtp9_1Za0BxNM4> .
}
```

**References**

[1] X. Rosa Ma, M. Prudencio, Y. Koike, S. C. Vatsavayai, G. Kim, F.Harbinski, C. M. Rodriguez, H. B. Schmidt, B. B. Cummings, D. W. Wyatt, K. Kurylo, G. Miller, S. Mekhoubad, N. Sallee, K. Jansen-West, C.N. Cook, S. Pickles, B. Oskarsson, N.R. Graff-Radford, B. F. Boeve, D. S. Knopman, R. C. Petersen, D. W. Dickson, E. M. Green, W. W. Seeley, L. Petrucelli, A. D. Gitler. TDP-43 represses cryptic exon inclusion in FTD/ALS gene UNC13A. biorxiv. doi: 10.1101/2021.04.02.438213

[2] Bucur, C.I., Kuhn, T., Ceolin, D., Ossenbruggen, J. van. Expressing high-level scientific claims with formal semantics. In: Proceedings of the 11th Knowledge Capture Conference 2021. doi: 10.1145/3460210.3493561.
