# Supplementary material for: Nanopublication-based semantic publishing and reviewing: a field study with formalization papers
Source: PeerJ Comput Sci. 2023 Feb 21;9:e1159. doi: 10.7717/peerj-cs.1159 (PMC10280262; doi:10.7717/peerj-cs.1159)
Supplement: Supplemental Information 2 [file peerj-cs-09-1159-s002.zip › formalization_papers_supplemental-main/call_for_papers/call_for_papers_DS_special_issue.pdf]

Special Issue with Formalization Papers at Data Science Journal

We, Cristina-Iulia Bucur, Tobias Kuhn, Davide Ceolin and Jacco van Ossenbruggen, are preparing a novel kind of special issue at our Data Science journal (by IOS Press). Each paper in this special issue will consist of RDF statements expressing a single scientific claim.

Such papers written in RDF can be shorter but also much more powerful than classical papers. Text mining, for example, is currently very difficult and shallow, whereas it will be a trivial task for such RDF papers, which are written with deep formal semantics from the start.

With this special issue, we want to formally publish the first such RDF papers. For a start, we focus on what we call “formalization papers”, which consist of an RDF representation of a claim from an already existing publication. The novel contribution of such a paper is therefore the formalization of the claim, but not the claim itself.

Do you want to be among the very first authors to publish such futuristic papers? If so, we’d be very happy to welcome you on board and guide you through the process.

Some further points:

- Authors of a formalization paper can formalize their own previously published claim, or a claim from a paper published by others (in the latter case, the formalization paper authors take credit for the formalization of the claim but not for the claim itself)
- Authors are expected to have some knowledge of RDF and knowledge representation
- All submissions will be peer-reviewed (also in RDF)
- Accepted submissions will be regularly published by IOS Press: they will get a DOI and will have the same bibliometric status as other papers (i.e. they will show up in Google Scholar, citation counts, etc.)
- Interested authors will receive close guidance on how to represent claims of their choosing in RDF (using Nanobench and based on our super-pattern ontology)
- Below you can find a mock-up of what these papers will finally look like, a screenshot of the tool to be used to create them, and the actual RDF representation (in the form of a nanopublication) to give you an idea
- First submissions are expected around June 2021. Decisions are expected around September 2021.

Get in touch with us (c.i.bucur@vu.nl), if you are interested in authoring such a formalization paper for this special issue. We can then give you further information about the process and answer any questions you might have.

This is a mock-up of what such formalization papers will eventually look like on the IOS Press website (we will add a bit of a narrative to make it compatible with current publishing practices):

A formalization of one of the main claims of "Error and attack tolerance of complex networks" by Réka Albert, Hawoong Jeong, and Albert-László Barabási.

Cite

Article type: Formalization Paper

Authors: Kuhn, Tobias[a; *]

Affiliations: [a] Department of Computer Science, Vrije Universiteit Amsterdam, The Netherlands.
E-mail: t.kuhn@vu.nl

Correspondence: [*] Corresponding author: Tobias Kuhn, Department of Computer Science, Vrije Universiteit Amsterdam, De Boelelaan 1081, 1081 HV Amsterdam, The Netherlands.
E-mail: t.kuhn@vu.nl.

DOI: 10.3233/DS-000000

Journal: Data Science, vol. 4, no. 1-2, 2021

Received 16 June 2021 | **Accepted** 31 June 2021 | **Published:** 14 July 2021

Nanopublication

Abstract

I present here a formalization of one of the main findings of the article "Error and attack tolerance of complex networks" by Réka Albert, Hawoong Jeong, and Albert-László Barabási, originally published in Nature in 2000.

1. Formalization

I will here briefly explain the formalization as specified in the nanopublication representation of this publication.

The main claim of [1] covered here can be informally expressed in the following sentence: *Many complex systems are vulnerable to attacks because of their structure as scale-free networks.*

...

References

[1] Albert, R., Jeong, H. & Barabási, AL. Error and attack tolerance of complex networks. Nature 406, 378–382 (2000). doi:10.1038/35019019.

This screenshot shows the interface (Nanobench) that will be used to create such formalizations (the same example as in the mock-up above is shown here):

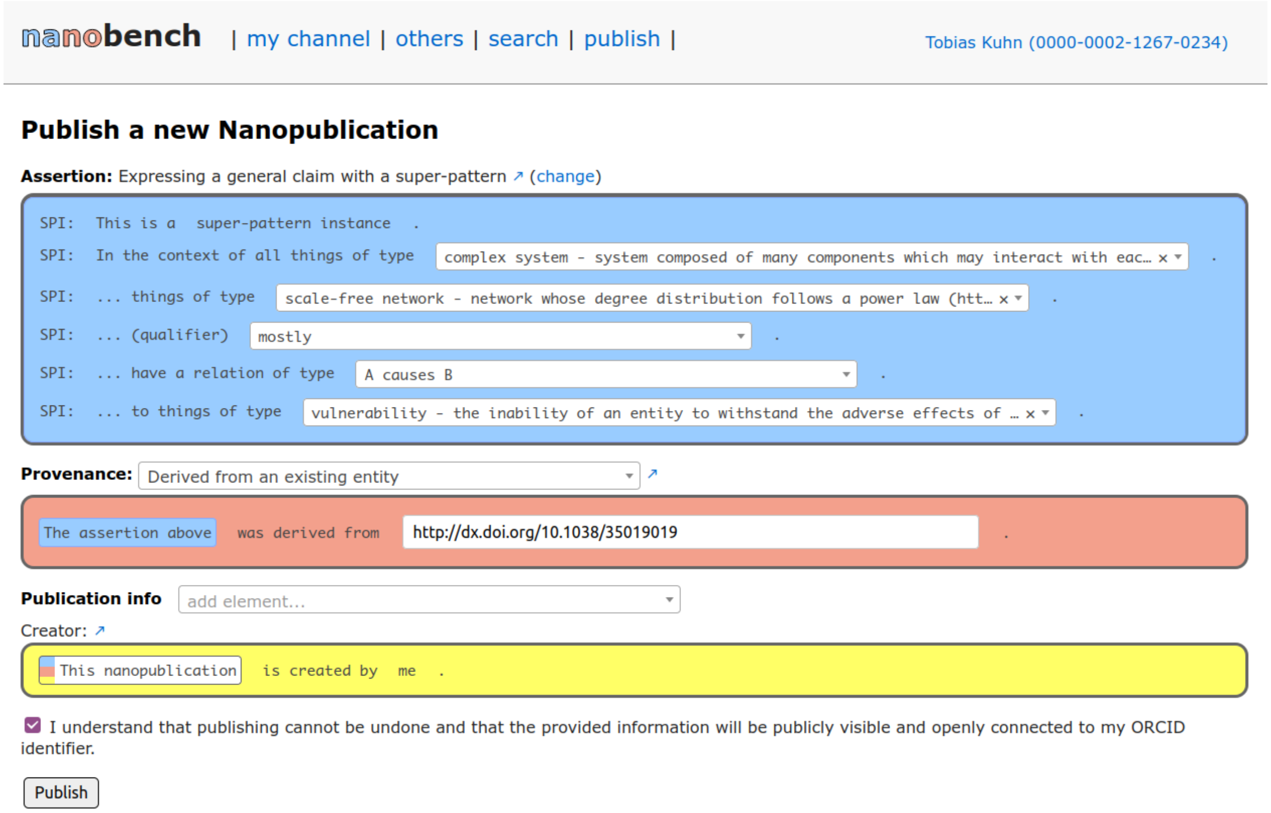

This is an example of a nanopublication containing a formalized RDF claim (this is the same example as above; [link to nanopublication]):

```
@prefix this: <http://purl.org/np/RAjU96IQai9rK7Ual5xKCoJVzSZAUfhgNE339IXHu9QD4> .
@prefix sub: <http://purl.org/np/RAjU96IQai9rK7Ual5xKCoJVzSZAUfhgNE339IXHu9QD4#> .
@prefix np: <http://www.nanopub.org/nschema#> .
@prefix dct: <http://purl.org/dc/terms/> .
@prefix nt: <https://w3id.org/np/o/ntemplate/> .
@prefix npx: <http://purl.org/nanopub/x/> .
@prefix xsd: <http://www.w3.org/2001/XMLSchema#> .
@prefix orcid: <https://orcid.org/> .
@prefix prov: <http://www.w3.org/ns/prov#> .
@prefix sp: <https://w3id.org/linkflows/superpattern/terms/> .

sub:Head {
  this: np:hasAssertion sub:assertion ;
    np:hasProvenance sub:provenance ;
    np:hasPublicationInfo sub:pubinfo ;
    a np:Nanopublication .
}

sub:assertion {
  sub:spi a sp:SuperPatternInstance ;
    sp:hasContextClass <http://www.wikidata.org/entity/Q854457> ;
    sp:hasObjectClass <http://www.wikidata.org/entity/Q1178984> ;
    sp:hasQualifier sp:mostlyQualifier ;
    sp:hasRelation sp:causes ;
    sp:hasSubjectClass <http://www.wikidata.org/entity/Q1071058> .
}

sub:provenance {
  sub:assertion prov:wasDerivedFrom <http://dx.doi.org/10.1038/35019019> .
}

sub:pubinfo {
  sub:sig npx:hasAlgorithm "RSA" ;
    npx:hasPublicKey
"MIGfMA0GCSqGSIb3DQEBAQUAA4GNADCBiQKBgQCwUtewGCpT5vIfXYE1bmf/Uqu1ojqnWdYxv+ySO80ul8Gu7m8KoyPAwuvaPj0lvPtHrg000qMmkxzKhYknEjq8v7EerxZNYp5B3
/3+5ZpuWOYAs78UnQVjbHSmDdmryr4D4VvvNIiUmd0yxci47dTFUj4DvfHnGd6hVe5+goqdcwIDAQAB" ;
    npx:hasSignature "nqQ+6MMugDt2LCA66osVBfK3F4ti0xHjN5n/VXnfiHCs4fjTpreTmfL9FKcANRMYAP21qykhsWQybrI6JGOoD4Z5E6cJH4ysbHt+70m4kb
/O4I2waVoAh+I36xN1zi38cCfSH89nyNKuakt21VOJJ47G2yXgebsSuX1x2Un8gls=" ;
    npx:hasSignatureTarget this: .
  this: dct:created "2021-02-05T17:09:44.025+01:00"^^xsd:dateTime ;
    dct:creator orcid:0000-0002-1267-0234 ;
    npx:introduces sub:spi ;
    nt:wasCreatedFromProvenanceTemplate <http://purl.org/np/RAcTpoh5Ra0ssqmcpOgWdaZ_YiPE6demO6cpw-2RvSNs8> ;
    nt:wasCreatedFromPubinfoTemplate <http://purl.org/np/RAA2MfqdBCzmz9yVWjKLXNbyfBNcwsMmOqcNUxkk1maIM> ;
    nt:wasCreatedFromTemplate <http://purl.org/np/RAxT_BF9NInSoGdP2lc3i0Xld367Xkl0Zhmjy8F9r5gGM> .
}
```
