# Supplementary material for: Nanopublication-based semantic publishing and reviewing: a field study with formalization papers
Source: PeerJ Comput Sci. 2023 Feb 21;9:e1159. doi: 10.7717/peerj-cs.1159 (PMC10280262; doi:10.7717/peerj-cs.1159)
Supplement: Supplemental Information 2 [file peerj-cs-09-1159-s002.zip › formalization_papers_supplemental-main/call_for_papers/initial_presentation_about_DS_special_issue.pdf]

DATA SCIENCE
Methods, Infrastructure, and Applications

Special Issue with Formalization Papers at Data Science Journal

Guidelines for Authors

Let’s publish scientific claims in formal logic!

Obesity when accompanied by metabolic abnormality is closely associated with knee osteoarthritis.

CONTEXT-CLASS (“in the context of all ..."): **Person**

SUBJECT-CLASS (“things of type ..."): **Obesity together with metabolic abnormality**

QUALIFIER: **generally***

RELATION-TYPE (“have a relation of type...”): **co-occurs with**

OBJECT-CLASS (“to things of type...”): **Knee osteoarthritis**

* this qualifier was changed to “frequently” in a new version

How can we represent high-level claims?

- Using the SuperPattern ontology

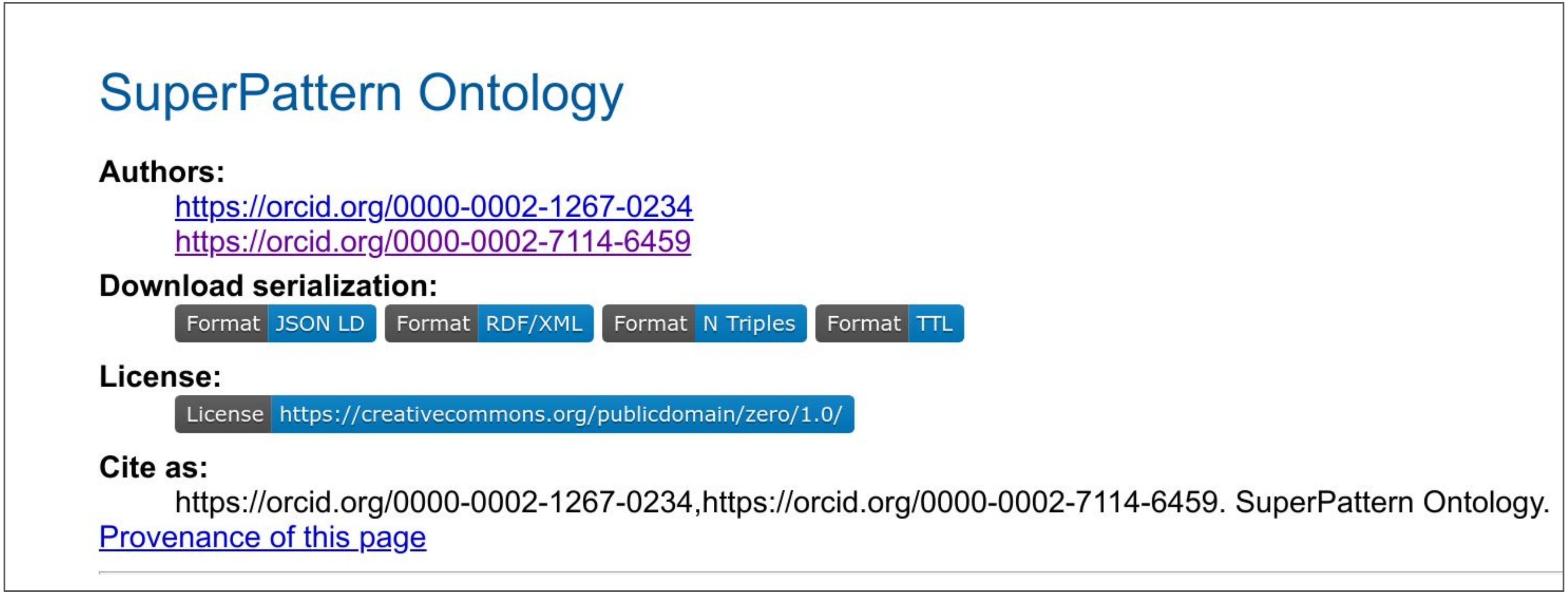

SuperPattern Ontology

Authors:

https://orcid.org/0000-0002-1267-0234
https://orcid.org/0000-0002-7114-6459

Download serialization:

Format JSON LD Format RDF/XML Format N Triples Format TTL

License:

License https://creativecommons.org/publicdomain/zero/1.0/

Cite as:

https://orcid.org/0000-0002-1267-0234,https://orcid.org/0000-0002-7114-6459. SuperPattern Ontology.

Provenance of this page

SuperPattern interpretation

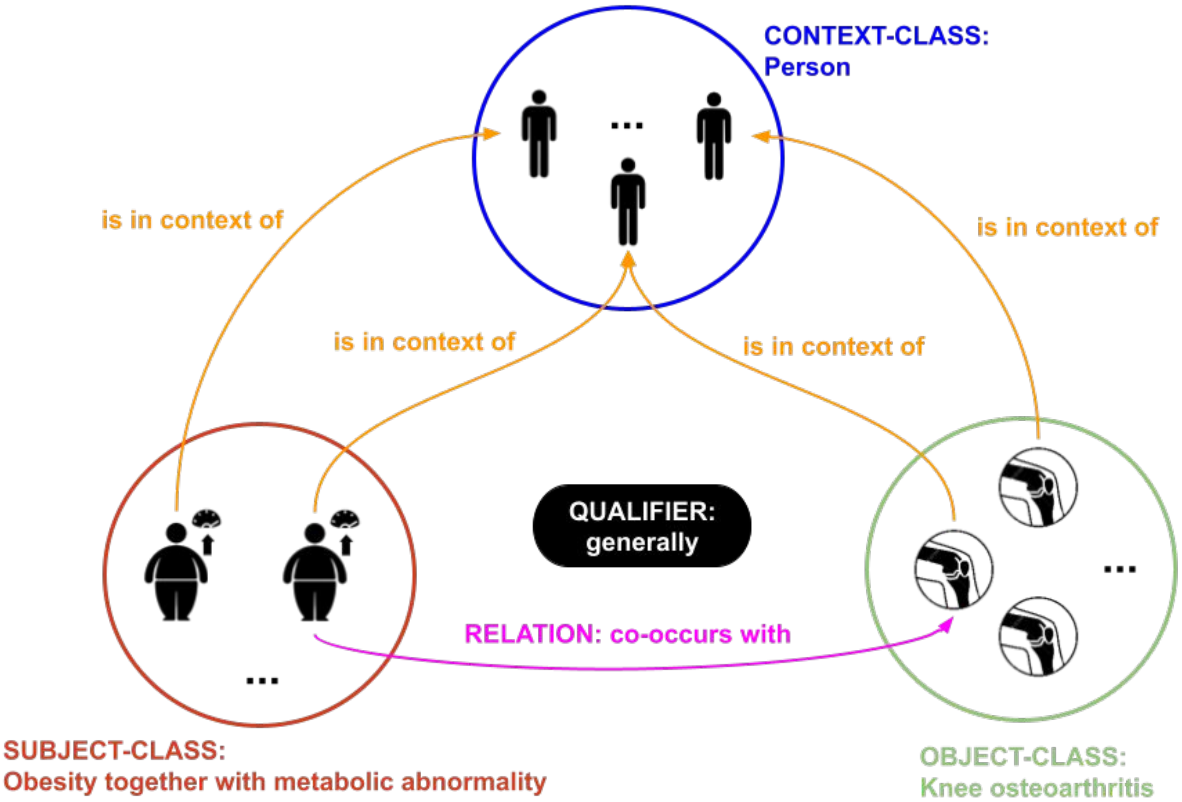

Informally:

Every thing of type **obesity together with metabolic abnormality** that is in the context of a thing of type **person** **generally*** (in at least 90% of the cases) has a relation of type "**co-occurs with**" to a thing of type **knee osteoarthritis** that is in the same context.

* this qualifier was changed to “frequently” (in at least 10% of the cases) in a new version

Publishing such claims as nanopublications

```
@prefix this: <http://purl.org/np/RAc5YYnYevWeUd1igtqMi_eKGVhxVj7IBZ9mTY8-aCNlI> .
@prefix sub: <http://purl.org/np/RAc5YYnYevWeUd1igtqMi_eKGVhxVj7IBZ9mTY8-aCNlI#> .
@prefix np: <http://www.nanopub.org/nschema#> .
@prefix dct: <http://purl.org/dc/terms/> .
@prefix nt: <https://w3id.org/np/o/ntemplate/> .
@prefix npx: <http://purl.org/nanopub/x/> .
@prefix xsd: <http://www.w3.org/2001/XMLSchema#> .
@prefix orcid: <https://orcid.org/> .
@prefix prov: <http://www.w3.org/ns/prov#> .
@prefix sp: <https://w3id.org/linkflows/superpattern/terms/> .

sub:Head {
  this: np:hasAssertion sub:assertion ;
    np:hasProvenance sub:provenance ;
    np:hasPublicationInfo sub:pubinfo ;
    a np:Nanopublication .
}

sub:assertion {
  sub:spi a sp:SuperPatternInstance ;
    sp:hasContextClass <http://www.wikidata.org/entity/Q5> ;
    sp:hasObjectClass <http://www.wikidata.org/entity/Q1777118> ;
    sp:hasQualifier sp:generallyQualifier ;
    sp:hasRelation sp:cooccursWith ;
    sp:hasSubjectClass <http://purl.org/np/RAVYczMihUbgryLu1x8xhoqR6LQe4fSoWmF3SCQHfR5xE#obesity-with-metabolic-abnormality> .
}

sub:provenance {
  sub:assertion prov:wasDerivedFrom <https://doi.org/10.3109/14397595.2014.939393> .
}

sub:pubinfo {
  sub:sig npx:hasAlgorithm "RSA" ;
    npx:hasPublicKey
"MIGfMA0GCSqGSIb3DQEBAQUAA4GNADCBiQKBgQCJlM78d80R+gFMoQB1IG3f7AbqqGOCIv4HmZd1cx1KgEWMUUpPsojFNvx84fC/TltcJ8F8JafnbhDXW2HM2MhdK4yC04ROEV1
;
    npx:hasSignature "YXzdgk7n75OhzaHpiJr7LW0T3PdUct5kBsEUDI+TiWEv8ZyH3bx406MRD6VxuGey0i2V+2/tSHmkc
/2P/MdbR9HqNxG8z7VXhsO7BjtYCF+MnNUFz1YlHj+gON9NyK9pFJwA2US1zkHl/CiTfKbO1hx+fwvN4eF5Yw4ykGyzEVE=" ;
    npx:hasSignatureTarget this: .
  this: dct:created "2021-05-07T15:35:00.337+03:00"^^xsd:dateTime ;
    dct:creator orcid:0000-0002-7114-6459 ;
    npx:introduces sub:spi ;
    npx:supersedes <http://purl.org/np/RA6Lpsa8P2nPzCY7ukUtnrYRlRtyFD1G6QVu8fgHJLxD0> ;
    nt:wasCreatedFromProvenanceTemplate <http://purl.org/np/RAcTpoh5Ra0ssqmcpOgWdaZ_YiPE6demO6cpw-2RvSNs8> ;
    nt:wasCreatedFromPubinfoTemplate <http://purl.org/np/RAA2MfqdBCzmz9yVWjKLXNbyfBNcwsMmOqcNUxkk1maIM> , <http://purl.org
/np/RAjpBMlw3owYhJUBo3DtsuDlXsNAJ8cnGeWAutDVjuAuI> ;
    nt:wasCreatedFromTemplate <http://purl.org/np/RAiL1AYhAZIPmCs9YXC8QaQl7rcMcnyeedEAPTi_BZR9Y> .
}
```

Publishing such claims as nanopublications

```
@prefix this: <http://purl.org/np/RAc5YYnYevWeUd1igtqMi_eKGVhxVj7IBZ9mTY8-aCNlI> .
@prefix sub: <http://purl.org/np/RAc5YYnYevWeUd1igtqMi_eKGVhxVj7IBZ9mTY8-aCNlI#> .
@prefix np: <http://www.nanopub.org/nschema#> .
@prefix dct: <http://purl.org/dc/terms/> .
@prefix nt: <https://w3id.org/np/o/ntemplate/> .
@prefix npx: <http://purl.org/nanopub/x/> .
@prefix xsd: <http://www.w3.org/2001/XMLSchema#> .
@prefix orcid: <https://orcid.org/> .
@prefix prov: <http://www.w3.org/ns/prov#> .
@prefix sp: <https://w3id.org/linkflows/superpattern/terms/> .

sub:Head {
  this: np:hasAssertion sub:assertion ;
    np:hasProvenance sub:provenance ;
    np:hasPublicationInfo sub:pubinfo ;
    a np:Nanopublication .
}

sub:assertion {
  sub:spi a sp:SuperPatternInstance ;
    sp:hasContextClass <http://www.wikidata.org/entity/Q5> ;
    sp:hasObjectClass <http://www.wikidata.org/entity/Q1777118> ;
    sp:hasQualifier sp:generallyQualifier ;
    sp:hasRelation sp:cooccursWith ;
    sp:hasSubjectClass <http://purl.org/np/RAVYczMihUbgryLu1x8xhoqR6LQe4fSoWmF3SCQHfR5xE#obesity-with-metabolic-abnormality> .
}

sub:provenance {
  sub:assertion prov:wasDerivedFrom <https://doi.org/10.3109/14397595.2014.939393> .
}

sub:pubinfo {
  sub:sig npx:hasAlgorithm "RSA" ;
    npx:hasPublicKey
"MIGfMA0GCSqGSIb3DQEBAQUAA4GNADCBiQKBgQCJlM78d80R+gFMoQB1IG3f7AbqqGOCIv4HmZd1cx1KgEWMUUpPsojFNvx84fC/TltcJ8F8JafnbhDXW2HM2MhdK4yC04ROEV1[illegible]
;
    npx:hasSignature "YXzdgk7n75OhzaHpiJr7LW0T3PdUct5kBsEUDI+TiWEv8ZyH3bx4O6MRD6VxuGey0i2V+2/tSHmkc
/2P/MdbR9HqNxG8z7VXhsO7BjtYCF+MnNUFz1YlHj+gON9NyK9pFJwA2US1zkHl/CiTfKbO1hx+fwvN4eF5Yw4ykGyzEVE=" ;
    npx:hasSignatureTarget this: .
  this: dct:created "2021-05-07T15:35:00.337+03:00"^^xsd:dateTime ;
    dct:creator orcid:0000-0002-7114-6459 ;
    npx:introduces sub:spi ;
    npx:supersedes <http://purl.org/np/RA6Lpsa8P2nPzCY7ukUtnrYRlRtyFD1G6QVu8fgHJLxD0> ;
    nt:wasCreatedFromProvenanceTemplate <http://purl.org/np/RAcTpoh5Ra0ssqmcpOgWdaZ_YiPE6demO6cpw-2RvSNs8> ;
    nt:wasCreatedFromPubinfoTemplate <http://purl.org/np/RAA2MfqdBCzmz9yVWjKLXNbyfBNcwsMmOqcNUxkk1maIM> , <http://purl.org
/np/RAjpBMlw3owYhJUBo3DtsuDlXsNAJ8cnGeWAutDVjuAuI> ;
    nt:wasCreatedFromTemplate <http://purl.org/np/RAiL1AYhAZIPmCs9YXC8QaQl7rcMcnyeedEAPTi_BZR9Y> .
}
```

Publishing such claims as nanopublications

```
sub:assertion {
  sub:spi a sp:SuperPatternInstance ;
    sp:hasContextClass <http://www.wikidata.org/entity/Q5> ;
    sp:hasObjectClass <http://www.wikidata.org/entity/Q1777118> ;
    sp:hasQualifier sp:generallyQualifier ;
    sp:hasRelation sp:cooccursWith ;
    sp:hasSubjectClass <http://purl.org/np/RAVYczMihUbgryLu1x8xhoqR6LQe4fSoWmF3SCQHfR5xE#obesity-with-metabolic-abnormality> .
}
```

CONTEXT-CLASS (“in the context of all ..."):	**Person**
SUBJECT-CLASS (“things of type ..."):	**Obesity together with metabolic abnormality**
QUALIFIER:	**generally***
RELATION-TYPE (“have a relation of type...”):	**co-occurs with**
OBJECT-CLASS (“to things of type...”):	**Knee osteoarthritis**

* this qualifier was changed to “frequently” in a new version

Nanobench: user interface for publishing nanopublications

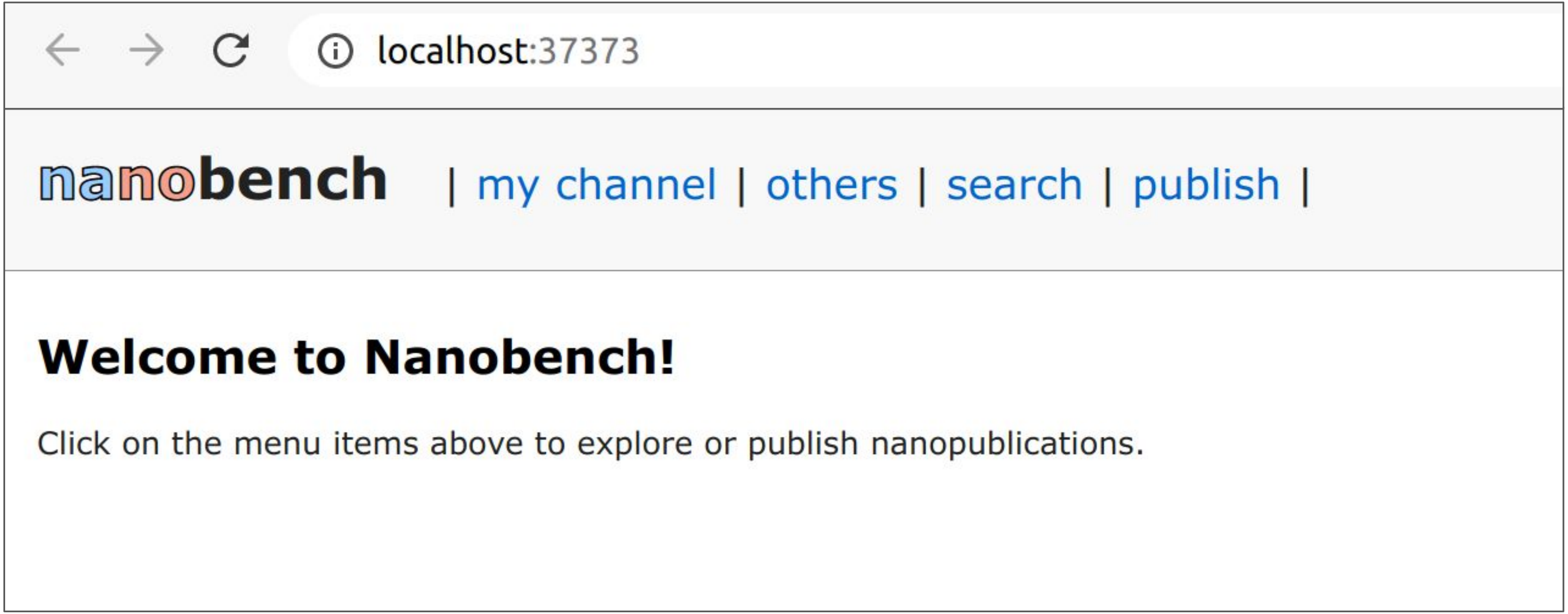

Task 1: Set up Nanobench (*now*)

- Nanobench installation instructions

- Nanobench installation instructions with Docker

Formalization paper

- A paper consisting of RDF statements that express a single scientific claim
- The scientific claim is from an existing publication
- Written with deep formal semantics from the start
- Contains a formalization of the scientific claim
- Peer-reviewed in RDF
- Same bibliometric status as a classic scientific paper (with DOIs, etc.)

Super-pattern template in Nanobench

Nanobench → Publish → Expressing a general claim with a super-pattern:

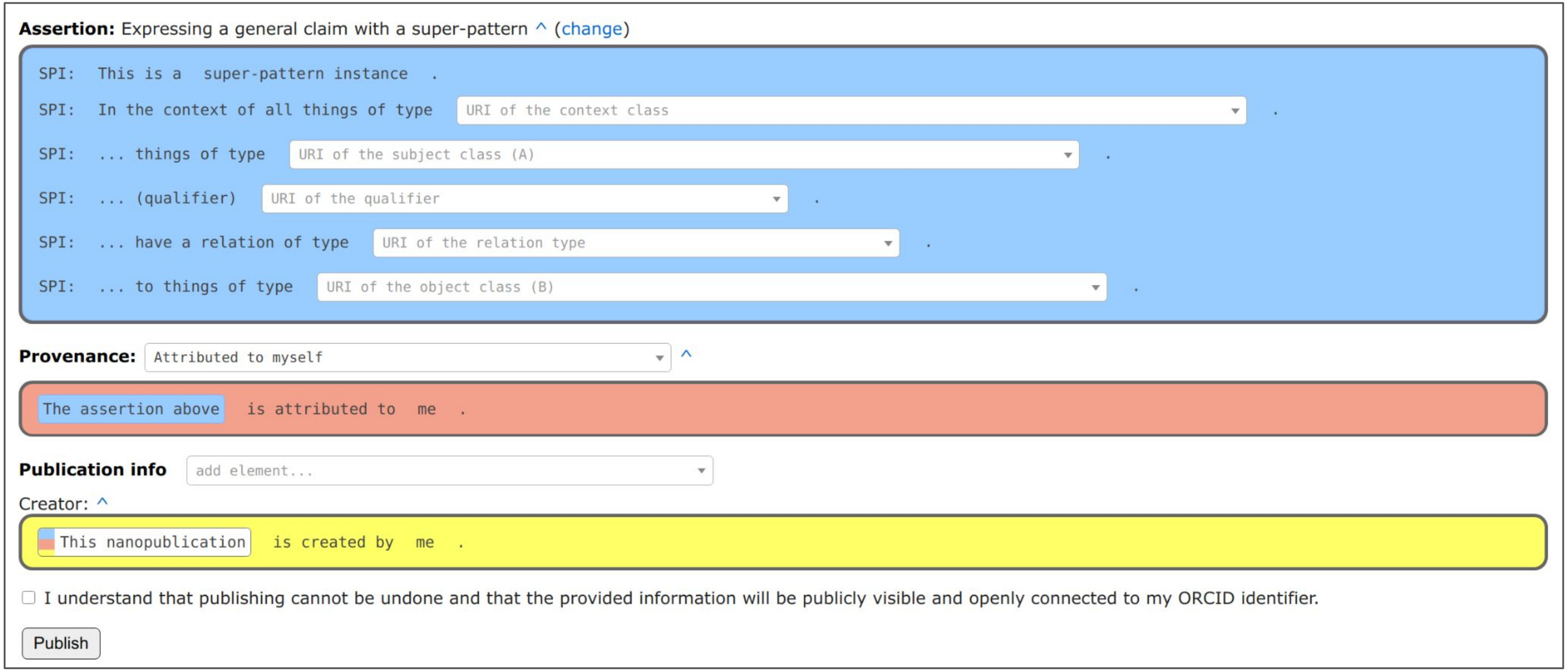

More examples of super-pattern statements

- **[Symbiosis and pathogenesis of Sinorhizobium meliloti share common molecular mechanisms.]**

```
CONTEXT-CLASS ("in the context of all ..."):    sinorhizobium-meliloti
SUBJECT-CLASS ("things of type ..."):           molecular-mechanism-symbiosis
QUALIFIER:                                      sometimes
RELATION-TYPE ("have a relation of type ..."): [subj] isSameAs [obj]
OBJECT-CLASS ("to things of type ..."):         molecular-mechanism-pathogenesis
```

More examples of super-pattern statements

- Symbiosis and pathogenesis of Sinorhizobium meliloti share common molecular mechanisms.
- **Lifelong education in the electro-mechanical industry requires extensive international knowledge of their workers.**

```
CONTEXT-CLASS (“in the context of all ..."):    worker-in-electromechanical-industry
SUBJECT-CLASS (“things of type ..."):           lifelong-education
QUALIFIER:                                      generally
RELATION-TYPE (“have a relation of type ..."):  [subj] requires [obj]
OBJECT-CLASS (“to things of type ..."):         extensive-international-knowledge
```

More examples of super-pattern statements

- Symbiosis and pathogenesis of Sinorhizobium meliloti share common molecular mechanisms.
- Lifelong education in the electro-mechanical industry requires extensive international knowledge of their workers.
- **Human ovarian and endometrial cancer cell lines are frequently misidentified.**

```
CONTEXT-CLASS (“in the context of all ..."):     NONE
SUBJECT-CLASS (“things of type ..."):            human-ovarian-cancer-cell-line OR
                                                 human-endometrial-cancer-cell-line
QUALIFIER:                                       frequently
RELATION-TYPE (“have a relation of type ..."):   [subj] includes [obj]
OBJECT-CLASS (“to things of type ..."):          misidentification
```

More examples of super-pattern statements

- Symbiosis and pathogenesis of Sinorhizobium meliloti share common molecular mechanisms.
- Lifelong education in the electro-mechanical industry requires extensive international knowledge of their workers.
- Human ovarian and endometrial cancer cell lines are frequently misidentified.
- **Inexpensive photocells for the Arduino-Visual Basic interface can be used for recording and controlling events in operant research.**

```
CONTEXT-CLASS (“in the context of all ..."):     operant-research
SUBJECT-CLASS (“things of type ..."):            inexpensive-photocell-for-arduino-vb-interface
QUALIFIER:                                       can generally
RELATION-TYPE (“have a relation of type ..."):   [subj] contributesTo [obj]
OBJECT-CLASS (“to things of type ..."):          recording-controlling-events-activity
```

More examples of super-pattern statements

- Symbiosis and pathogenesis of Sinorhizobium meliloti share common molecular mechanisms.
- Lifelong education in the electro-mechanical industry requires extensive international knowledge of their workers.
- Human ovarian and endometrial cancer cell lines are frequently misidentified.
- Inexpensive photocells for the Arduino-Visual Basic interface can be used for recording and controlling events in operant research.
- **Solid and cancellous autogenous bone grafts are used in the treatment of acute bone loss and nonunion.**

```
CONTEXT-CLASS ("in the context of all ..."):   acute-bone-loss OR acute-bone-nonunion
SUBJECT-CLASS ("things of type ..."):          solid-autogenous-bone-graft OR
                                               cancellous-autogenous-bone-graft
QUALIFIER:                                     can generally
RELATION-TYPE ("have a relation of type ..."): [subj] contributesTo [obj]
OBJECT-CLASS ("to things of type ..."):        treatment
```

More examples of super-pattern statements

- Symbiosis and pathogenesis of Sinorhizobium meliloti share common molecular mechanisms.
- Lifelong education in the electro-mechanical industry requires extensive international knowledge of their workers.
- Human ovarian and endometrial cancer cell lines are frequently misidentified.
- Inexpensive photocells for the Arduino-Visual Basic interface can be used for recording and controlling events in operant research.
- Solid and cancellous autogenous bone grafts are used in the treatment of acute bone loss and nonunion.

Helper! You will be provided with a helper document that can be used to create your scientific claim and its basic formalization.

Task 2: Pick paper with claim to formalize (*May*)

- Find a scientific paper (yours or somebody else's) from which to formalize one of its high-level scientific claims
- As an intermediate step, you may want to express your claim as an AIDA sentence
  - expressing claims in a general way
  - AIDA sentences: English sentences that are Atomic, Independent, Declarative, and Absolute
  - absoluteness property: excluding information about how the claim was derived and how certain we can be about its truth

Task 2: Pick paper with claim to formalize (*May*)

- Lee S. et al. Obesity, metabolic abnormality, and knee osteoarthritis: a cross-sectional study in Korean women. Mod Rheumatol. 2015 Mar;25(2):292-7. doi: 10.3109/14397595.2014.939393.

- Scientific claim expressed as an AIDA sentence:

Obesity when accompanied by metabolic abnormality is closely associated with knee osteoarthritis.

Task 3: Create abstract version of instantiated super-pattern (*May*)

CONTEXT-CLASS (“in the context of all ..."):	**Person**
SUBJECT-CLASS (“things of type ..."):	**Obesity together with metabolic abnormality**
QUALIFIER:	**generally***
RELATION-TYPE (“have a relation of type...”):	**co-occurs with**
OBJECT-CLASS (“to things of type...”):	**Knee osteoarthritis**

- Use the SuperPatter ontology to find the qualifier and the relation type that is the closest to the desired representation

* this qualifier was changed to “frequently” in a new version

Task 3: Create abstract version of instantiated super-pattern (*May*)

CONTEXT-CLASS ("in the context of all ..."):	**Person**
SUBJECT-CLASS ("things of type ..."):	**Obesity together with metabolic abnormality**
QUALIFIER:	**generally***
RELATION-TYPE ("have a relation of type..."):	**co-occurs with**
OBJECT-CLASS ("to things of type..."):	**Knee osteoarthritis**

- Link new classes to WikiData where possible
  - Person (~Human)
  - Knee osteoarthritis
- Nanobench connects to WikiData and provides autocomplete when filling in forms

* this qualifier was changed to "frequently" in a new version

Task 4: Define new classes where needed (*June*)

CONTEXT-CLASS (“in the context of all ..."): **Person**
SUBJECT-CLASS (“things of type ..."): **Obesity together with metabolic abnormality**
QUALIFIER: **generally***
RELATION-TYPE (“have a relation of type...”): **co-occurs with**
OBJECT-CLASS (“to things of type...”): **Knee osteoarthritis**

- *Nanobench → Publish → Defining a new class*
  (we’ll worry about the class definitions later; discuss it with us)

Assertion: Defining a new class ^ (change)

obesity-with-metabolic-abnormal is a class .
obesity-with-metabolic-abnormal is called " obesity with metabolic abnormality " .
obesity-with-metabolic-abnormal is defined as follows: " Obesity conditions together with metabolic abnormality. " .
obesity-with-metabolic-abnormal is a subclass of https://www.wikidata.org/Q12174 × ▾ . *(optional)*

* this qualifier was changed to “frequently” in a new version

Task 5: Publish the first version of your super-pattern instance with Nanobench (*June*)

Nanobench → Publish → Expressing a general claim with a super-pattern:

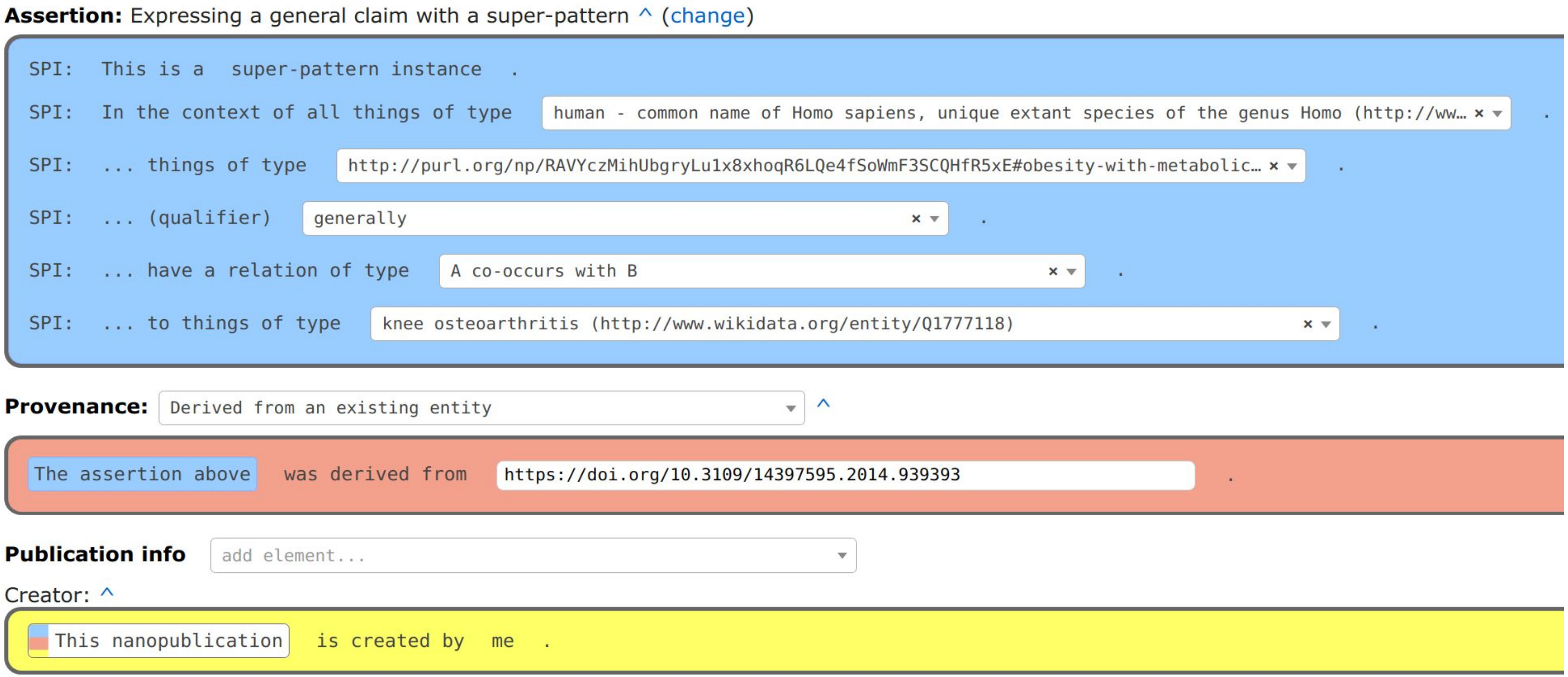

Task 6: Review some other contributions (*June-August*)

Nanobench → Publish → Making a review comment according to the LinkFlows model:

Publish a new Nanopublication

Assertion: Making a review comment according to the LinkFlows model ^ (change)

This comment is a review comment .
This comment refers to [the URL of the paper or text this review comment is about] .
This comment is a [comment about the content] .
This comment is a [negative comment] .
This comment is a [comment that declares that an action is needed] .
This comment has impact level "[integer value from 1 (low impact) to 5 (high impact)]" .
This comment has the comment text "[The content of this comment as free text]" .

Provenance: Attributed to myself ^

The assertion above is attributed to me .

Publication info add element...

Creator: ^

This nanopublication is created by me .

☐ I understand that publishing cannot be undone and that the provided information will be publicly visible and openly connected to my ORCID identifier.

Publish

Task 7: Improve your contributions based on reviews and publish new version (*July-August*)

Nanobench → my channel:

```
http://purl.org/np/RAc5YYnYevWeUd1igtqMi_eKGVhxVj7IBZ9mTY8-aCNII   | Actions: comment... retract... update...

spi   type   SuperPatternInstance   .
spi   hasContextClass   Q5   .
spi   hasObjectClass   Q1777118   .
spi   hasQualifier   generallyQualifier   .
spi   hasRelation   cooccursWith   .
spi   hasSubjectClass   obesity-with-metabolic-abnormality   .

this assertion   wasDerivedFrom   14397595.2014.939393   .

sig   hasAlgorithm   "RSA"   .
sig   hasPublicKey
"MIGfMA0GCSqGSIb3DQEBAQUAA4GNADCBiQKBgQCJlM78d80R+gFMoQB1IG3f7AbqqGOCIv4HmZd1cx1KgEWMUUpPsojFNvx84fC/TltcJ8F8
mmCEY7mUoeEW10mWZqjk+S9TnmiAQbFGcpExP8aosr2aTR7CSQIDAQAB"   .
sig   hasSignature
"YXzdgk7n75OhzaHpiJr7LW0T3PdUct5kBsEUDI+TiWEv8ZyH3bx4O6MRD6VxuGey0i2V+2/tSHmkc/2P/MdbR9HqNxG8z7VXhsO7BjtYCF+M
Yw4ykGyzEVE="   .
sig   hasSignatureTarget   this   .
this   created   "2021-05-07T15:35:00.337+03:00"   .
this   creator   0000-0002-7114-6459   .
this   introduces   spi   .
this   supersedes   RA6Lpsa8P2   .
this   wasCreatedFromProvenanceTemplate   RAcTpoh5Ra   .
this   wasCreatedFromPubinfoTemplate   RAA2MfqdBC   .
this   wasCreatedFromPubinfoTemplate   RAjpBMlw3o   .
this   wasCreatedFromTemplate   RAiL1AYhAZ   .
```

Task 8: If accepted: Make official final version (*September*)

Publish a new Nanopublication

Assertion: Expressing a general claim with a super-pattern ^ (change)

SPI: This is a super-pattern instance .

SPI: In the context of all things of type human - common name of Homo sapiens, unique extant species of the genus Homo (http://ww… .

SPI: ... things of type http://purl.org/np/RAVYczMihUbgryLu1x8xhoqR6LQe4fSoWmF3SCQHfR5xE#obesity-with-metabolic… .

SPI: ... (qualifier) generally .

SPI: ... have a relation of type A co-occurs with B .

SPI: ... to things of type knee osteoarthritis (http://www.wikidata.org/entity/Q1777118) .

Provenance: Derived from an existing entity ^

The assertion above was derived from https://doi.org/10.3109/14397595.2014.939393 .

Publication info add element...

Creator: ^

This nanopublication is created by me .

This is what it will look like eventually (roughly)

A formalization of one of the main claims of "Obesity, metabolic abnormality, and knee osteoarthritis: a cross-sectional study in Korean women" by Lee S. et al.

Cite

Article type: Formalization Paper

Authors: Kuhn, Tobias[a; *]

Affiliations: [a] Department of Computer Science, Vrije Universiteit Amsterdam, The Netherlands.
E-mail: t.kuhn@vu.nl

Correspondence: [*] Corresponding author: Tobias Kuhn, Department of Computer Science, Vrije Universiteit Amsterdam, De Boelelaan 1081, 1081 HV Amsterdam, The Netherlands.
E-mail: t.kuhn@vu.nl.

DOI: 10.3233/DS-000000

Journal: Data Science, vol. 4, no. 1-2, 2021

Received 16 June 2021 | **Accepted** 31 June 2021 | **Published:** 14 July 2021

Nanopublication

Abstract
I present here a formalization of one of the main findings of the article "Obesity, metabolic abnormality, and knee osteoarthritis: a cross-sectional study in Korean women" by Lee S. et al., originally published in Modern Rheumatology in 2015.

1. Formalization

I will here briefly explain the formalization as specified in the nanopublication representation of this publication.

The main claim of [1] covered here can be informally expressed in the following sentence: *Obesity when accompanied by metabolic abnormality is closely associated with knee osteoarthritis.*

...

References

[1] Lee S. et al. Obesity, metabolic abnormality, and knee osteoarthritis: a cross-sectional study in Korean women. Mod Rheumatol. 2015 Mar;25(2):292-7. doi: 10.3109/14397595.2014.939393.

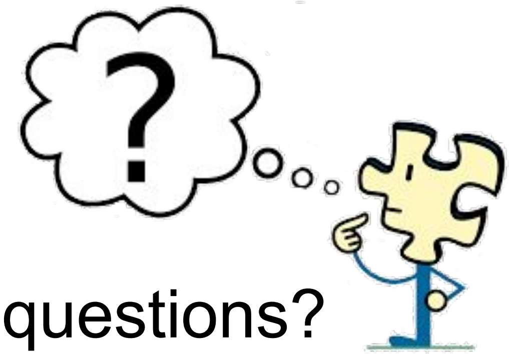

Any questions?

Feel free to reach out to us:

Cristina Bucur: c.i.bucur@vu.nl
Tobias Kuhn: t.kuhn@vu.nl
