# Supplementary material for: Nanopublication-based semantic publishing and reviewing: a field study with formalization papers
Source: PeerJ Comput Sci. 2023 Feb 21;9:e1159. doi: 10.7717/peerj-cs.1159 (PMC10280262; doi:10.7717/peerj-cs.1159)
Supplement: Supplemental Information 2 [file peerj-cs-09-1159-s002.zip › formalization_papers_supplemental-main/questionnaire/questionnaire_form.pdf]

Questionnaire Formalization Papers

Please let us know about your experience regarding formalization papers.

* Required

Part 1: Conceptual Aspects

1. How difficult or easy was it for you to CONCEPTUALLY understand ... *

Mark only one oval per row.

	1: very difficult	2	3	4	5: very easy
... what a formalization paper is?	⬭	⬭	⬭	⬭	⬭
... the purpose of the super-pattern?	⬭	⬭	⬭	⬭	⬭
... the role of the context class?	⬭	⬭	⬭	⬭	⬭
... the role of the subject class?	⬭	⬭	⬭	⬭	⬭
... the super-pattern qualifiers ("generally", etc.)?	⬭	⬭	⬭	⬭	⬭
... the super-pattern relations ("causes", etc.)?	⬭	⬭	⬭	⬭	⬭
... the role of the object class?	⬭	⬭	⬭	⬭	⬭
... the overall interpretation of the super-pattern?	⬭	⬭	⬭	⬭	⬭

2. How difficult or easy was it ... *

Mark only one oval per row.

	1: very difficult	2	3	4	5: very easy
... to find an article with a claim to formalize?					
... to understand what the claim exactly meant?					
... to decide on the context class?					
... to decide on the subject class?					
... to decide on the super-pattern qualifier?					
... to decide on the super-pattern relation?					
... to decide on the object class?					
... to conceptually represent the claim with the super-pattern overall?					

Part 2: Technical Aspects

You had to use two tools: the Nanobench application and the Tapas interface. The screenshots below can remind you which was which. If you did not take part in the technical step of creating the formalization in the case of joint authorship, you can skip this part.

Nanobench

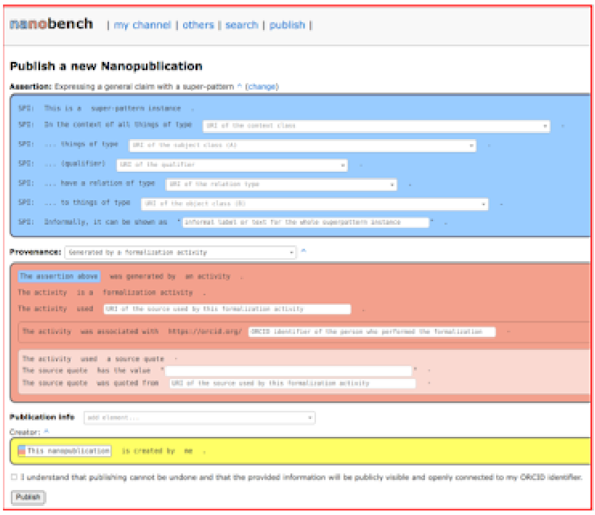

Tapas

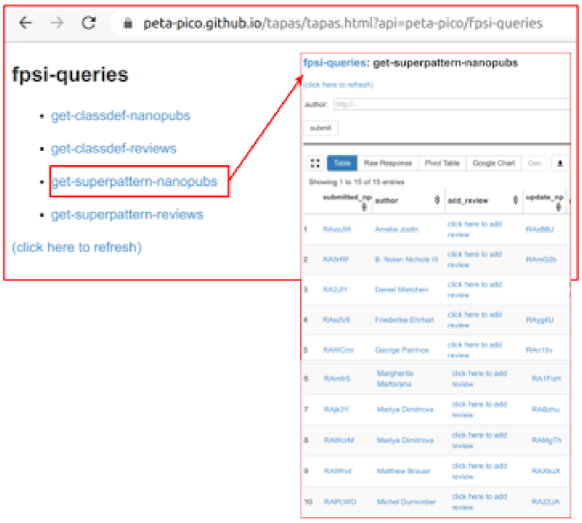

3. How difficult or easy was it for you to setup Nanobench?

Mark only one oval.

	1	2	3	4	5	
very difficult						very easy

4. How difficult or easy was it for you to use the given tools?

Mark only one oval per row.

	1: very difficult	2	3	4	5: very easy
Nanobench					
Tapas					

5. At the SUBMISSION stage, how difficult or easy was it for you with the given tools (Nanobench and Tapas) to ...

Mark only one oval per row.

	1: very difficult	2	3	4	5: very easy
... define new classes (if you did)?					
... select the right qualifier, relation and classes for the formalization?					
... fill in the provenance part (in red)?					
... publish your formalization?					
... submit your formalization to the special issue?					

6. At the REVIEWING stage, how difficult or easy was it for you with the given tools (Nanobench and Tapas) to ...

Mark only one oval per row.

	1: very difficult	2	3	4	5: very easy
... publish review comments for others (if you did)?	⬭	⬭	⬭	⬭	⬭
... view received review comments?	⬭	⬭	⬭	⬭	⬭

7. At the FINAL REVISION stage, how difficult or easy was it for you with the given tools (Nanobench and Tapas) to ...

Mark only one oval per row.

	1: very difficult	2	3	4	5: very easy
... update your classes in response to received review comments? (if you did)	⬭	⬭	⬭	⬭	⬭
... update your formalization in response to received review comments?	⬭	⬭	⬭	⬭	⬭
... respond to review comments?	⬭	⬭	⬭	⬭	⬭

Part 3: General Aspects

8. How confident are you, as an author, in the quality of your formalization? *

Mark only one oval.

	1	2	3	4	5	
not confident at all	⬭	⬭	⬭	⬭	⬭	very confident

9. How important do you think was it that you created the final formalization yourself with the Nanobench tool, instead of the editor or publisher doing the final touches on your behalf (similar to copy-editing)? *

Mark only one oval.

	1	2	3	4	5	
not important at all	⬭	⬭	⬭	⬭	⬭	very important

10. In the future, if given the opportunity, how interested would you be in publishing such formalizations along with the publication of your articles? *

Mark only one oval.

	1	2	3	4	5	
not interested at all	⬭	⬭	⬭	⬭	⬭	very interested

11. For these two possible views of formalization papers, how important do you think it is that they are made available by the publisher for (human) website visitors? *

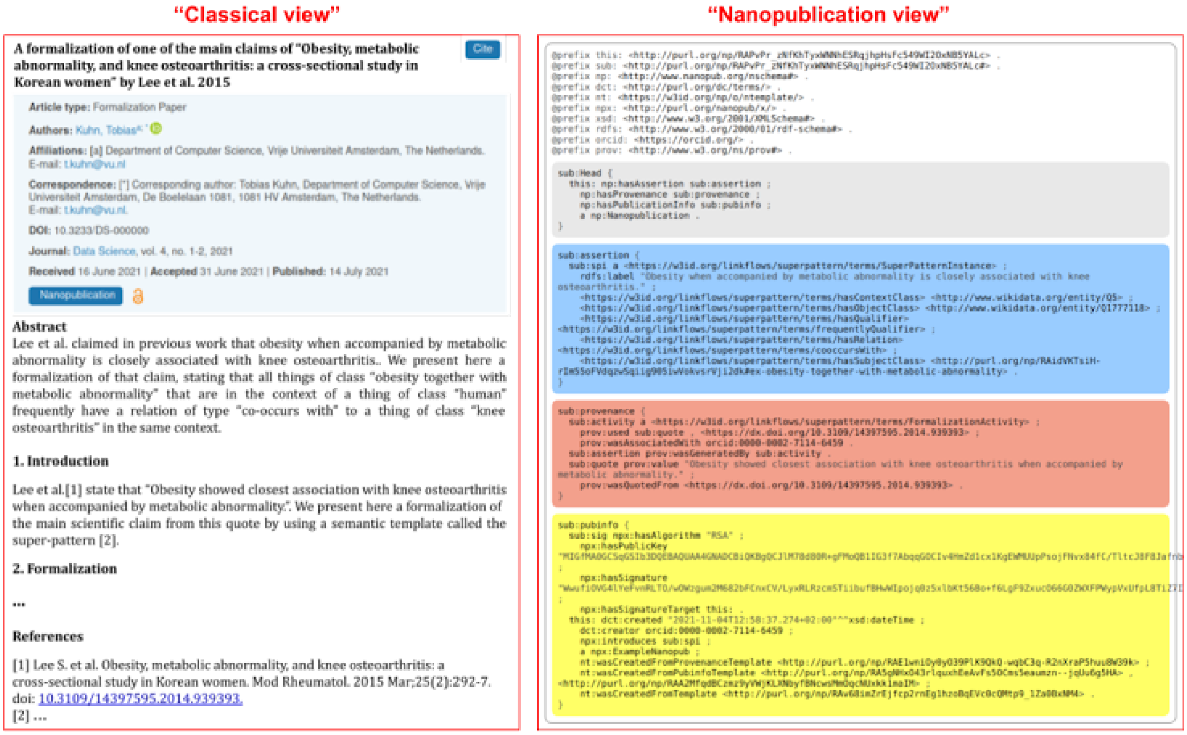

Mark only one oval per row.

	1: not important at all	2	3	4	5: very important
"classical view"	◯	◯	◯	◯	◯
"nanopublication view"	◯	◯	◯	◯	◯

Part 4: Your Background

12. How would you rate your knowledge with respect to the following topics? *

Mark only one oval per row.

	1: none	2	3	4	5: expert
Knowledge representation					
Knowledge graphs/Linked Data					
Ontologies/vocabularies					
Nanopublications					
Formal logic					
Programming languages					

13. You can write any further comments here (optional).
