# Supplementary material for: Nanopublication-based semantic publishing and reviewing: a field study with formalization papers
Source: PeerJ Comput Sci. 2023 Feb 21;9:e1159. doi: 10.7717/peerj-cs.1159 (PMC10280262; doi:10.7717/peerj-cs.1159)
Supplement: Supplemental Information 2 [file peerj-cs-09-1159-s002.zip › formalization_papers_supplemental-main/questionnaire/questionnaire_responses.pdf]

How difficult or easy was it for you to CONCEPTUALLY understand ... [... what a formalization paper is?]	How difficult or easy was it for you to CONCEPTUALLY understand ... [... the purpose of the super-pattern?]	How difficult or easy was it for you to CONCEPTUALLY understand ... [... the role of the context class?]	How difficult or easy was it for you to CONCEPTUALLY understand ... [... the role of the subject class?]	How difficult or easy was it for you to CONCEPTUALLY understand ... [... the super-pattern qualifiers ("generally", etc.)?]	How difficult or easy was it for you to CONCEPTUALLY understand ... [... the super-pattern relations ("causes", etc.)?]
4	3	3	4	4	4
5: very easy	3	3	3	3	3
5: very easy	4	3	3	3	4
3	2	1: very difficult	4	2	3
5: very easy	4	4	4	3	3
4	5: very easy	5: very easy	5: very easy	5: very easy	5: very easy
4	4	4	5: very easy	5: very easy	5: very easy
4	5: very easy	4	4	3	4
3	4	3	2	4	3
5: very easy	5: very easy	4	5: very easy	5: very easy	5: very easy
5: very easy	4	4	4	4	4
4	5: very easy	5: very easy	5: very easy	3	4
5: very easy	4	4	5: very easy	4	4
4	3	3	3	4	4
5: very easy	3	3	3	3	3
5: very easy	4	3	3	4	4
4	4	4	4	4	4
3	3	4	4	3	4
5: very easy	5: very easy	4	4	4	4

How difficult or easy was it for you to CONCEPTUALLY understand ... [... the role of the object class?]	How difficult or easy was it for you to CONCEPTUALLY understand ... [... the overall interpretation of the super-pattern?]	How difficult or easy was it ... [... to find an article with a claim to formalize?]	How difficult or easy was it ... [... to understand what the claim exactly meant?]	How difficult or easy was it ... [... to decide on the context class?]	How difficult or easy was it ... [... to decide on the subject class?]
4	4	3	3	2	3
3	3	5: very easy	5: very easy	5: very easy	4
3	3	4	3	2	2
4	2	5: very easy	2	1: very difficult	2
4	5: very easy	2	4	4	4
5: very easy	5: very easy	5: very easy	4	2	2
5: very easy	4	4	4	1: very difficult	1: very difficult
4	4	5: very easy	5: very easy	4	4
3	3	4	2	4	1: very difficult
5: very easy	5: very easy	3	4	3	5: very easy
4	4	3	4	2	3
5: very easy	5: very easy	4	4	5: very easy	4
5: very easy	4	3	4	2	2
3	2	2	4	3	3
3	3	2	3	3	3
3	3	4	3	2	2
4	4	5: very easy	5: very easy	4	4
4	4	3	3	3	2
4	4	5: very easy	5: very easy	3	2

How difficult or easy was it ... [... to decide on the super-pattern qualifier?]	How difficult or easy was it ... [... to decide on the super-pattern relation?]	How difficult or easy was it ... [... to decide on the object class?]	How difficult or easy was it ... [... to conceptually represent the claim with the super-pattern overall?]	How difficult or easy was it for you to setup Nanobench?	How difficult or easy was it for you to use the given tools? [Nanobench]
2	3	3	3	4	4
4	4	4	4	3	3
2	2	2	2	3	2
3	3	2	1: very difficult	4	4
3	3	4	4	4	4
4	3	4	1: very difficult	3	1: very difficult
4	4	3	2	5	5: very easy
3	3	4	4	5	4
4	3	2	3	4	3
5: very easy	5: very easy	4	4	5	5: very easy
3	3	3	2	5	4
4	4	4	3	4	4
3	2	3	3	3	3
3	3	3	2	5	4
3	3	3	3	1	2
1: very difficult	1: very difficult	1: very difficult	1: very difficult	3	2
4	4	4	4	3	3
2	3	4	4	5	3
3	3	3	3	4	3

How difficult or easy was it for you to use the given tools? [Tapas]	At the SUBMISSION stage, how difficult or easy was it for you with the given tools (Nanobench and Tapas) to ... [... define new classes (if you did)?]	At the SUBMISSION stage, how difficult or easy was it for you with the given tools (Nanobench and Tapas) to ... [... select the right qualifier, relation and classes for the formalization?]	At the SUBMISSION stage, how difficult or easy was it for you with the given tools (Nanobench and Tapas) to ... [... fill in the provenance part (in red)?]	At the SUBMISSION stage, how difficult or easy was it for you with the given tools (Nanobench and Tapas) to ... [... publish your formalization?]	At the SUBMISSION stage, how difficult or easy was it for you with the given tools (Nanobench and Tapas) to ... [... submit your formalization to the special issue?]
	4	4	4	4	4
3	4	4	4	4	4
2	1: very difficult	1: very difficult	2	3	3
2	3	3	2	4	3
5: very easy	5: very easy	3	5: very easy	5: very easy	5: very easy
1: very difficult	1: very difficult	1: very difficult	1: very difficult	4	1: very difficult
3	4	4	4	5: very easy	5: very easy
4	4	3	4	4	4
3	3	4	4	5: very easy	4
3	4	5: very easy	4	4	4
3	3	3	3	4	4
3	4	5: very easy	5: very easy	5: very easy	5: very easy
3	3	4	3	5: very easy	4
	2	4	3	2	2
2	2	2	3	3	3
2	2	2	2	3	3
3	4	4	4	4	4
2	3	3	3	3	3
3	3	3	4	4	4

At the REVIEWING stage, how difficult or easy was it for you with the given tools (Nanobench and Tapas) to ... [... publish review comments for others (if you did)?]	At the REVIEWING stage, how difficult or easy was it for you with the given tools (Nanobench and Tapas) to ... [... view received review comments?]	At the FINAL REVISION stage, how difficult or easy was it for you with the given tools (Nanobench and Tapas) to ... [... update your classes in response to received review comments? (if you did)]	At the FINAL REVISION stage, how difficult or easy was it for you with the given tools (Nanobench and Tapas) to ... [... update your formalization in response to received review comments?]	At the FINAL REVISION stage, how difficult or easy was it for you with the given tools (Nanobench and Tapas) to ... [... respond to review comments?]	How confident are you, as an author, in the quality of your formalization?
3	3	4	4	4	3
2	2	3	3	2	4
2	2	2	2	3	4
2	2	2	2	2	4
5: very easy	5: very easy	4	4	5: very easy	4
1: very difficult	1: very difficult	1: very difficult	1: very difficult	1: very difficult	5
4	5: very easy	3	2	4	5
4	4	4	4	4	4
3	3	3	3	4	4
3	5: very easy	3	4	4	5
3	3	3	4	3	3
4	4	3	3	3	3
3	4	3	1: very difficult	2	4
	3	3	3	4	4
3	3	3	3	3	4
2	2	2	2	2	4
4	4	4	4	4	4
	2	3	3	2	4
4	4	3	4	4	4

How important do you think was it that you created the final formalization yourself with the Nanobench tool, instead of the editor or publisher doing the final touches on your behalf (similar to copy-editing)?	In the future, if given the opportunity, how interested would you be in publishing such formalizations along with the publication of your articles?	For these two possible views of formalization papers, how important do you think it is that they are made available by the publisher for (human) website visitors? ["classical view"]	For these two possible views of formalization papers, how important do you think it is that they are made available by the publisher for (human) website visitors? ["nanopublication view"]	How would you rate your knowledge with respect to the following topics? [Knowledge representation]	How would you rate your knowledge with respect to the following topics? [Knowledge graphs/Linked Data]
3	3	4	2	4	3
4	4	3	3	5: expert	5: expert
5	5	5: very important	5: very important	2	3
2	5	5: very important	2	4	4
4	3	5: very important	3	5: expert	5: expert
2	5	4	1: not important at all	3	3
3	5	5: very important	2	5: expert	5: expert
5	5	5: very important	5: very important	4	4
3	4	5: very important	3	4	5: expert
5	5	5: very important	3	4	5: expert
4	2	5: very important	4	4	4
4	4	5: very important	3	3	4
4	5	4	3	5: expert	4
2	4	5: very important	2	3	2
4	4	4	5: very important	4	4
4	2	5: very important	5: very important	2	2
4	4	5: very important	5: very important	4	4
4	3	5: very important	3	5: expert	5: expert
4	5	5: very important	4	4	4

How would you rate your knowledge with respect to the following topics? [Ontologies/vocabularies]	How would you rate your knowledge with respect to the following topics? [Nanopublications]	How would you rate your knowledge with respect to the following topics? [Formal logic]	How would you rate your knowledge with respect to the following topics? [Programming languages]
4	3	3	5: expert
5: expert	5: expert	4	2
3	4	2	1: none
4	2	3	4
5: expert	3	4	5: expert
3	3	3	3
5: expert	5: expert	5: expert	5: expert
4	3	2	4
5: expert	4	4	4
5: expert	5: expert	4	4
5: expert	3	2	2
3	3	1: none	4
4	3	3	3
3	2	2	3
3	5: expert	4	2
2	3	2	1: none
4	4	4	4
5: expert	3	3	5: expert
4	3	3	5: expert
